# Organic Room‐Temperature near‐IR Phosphorescence Harvested by Intramolecular Through‐Space Sensitization in Composite Molecules

**DOI:** 10.1002/anie.202503327

**Published:** 2025-03-23

**Authors:** Iida Partanen, Chao‐Hsien Hsu, Emily Hsue‐Chi Shi, Iván Maisuls, Toni Eskelinen, Antti J. Karttunen, Jarkko J. Saarinen, Cristian A. Strassert, Andrey Belyaev, Pi‐Tai Chou, Igor O. Koshevoy

**Affiliations:** ^1^ Department of Chemistry and Sustainable Technology University of Eastern Finland Yliopistokatu 7 Joensuu 80101 Finland; ^2^ Department of Chemistry National Taiwan University Taipei Taiwan 10617 China; ^3^ Institut für Anorganische und Analytische Chemie Universität Münster, CiMIC, SoN, CeNTech Heisenbergstraße 11 Münster 48149 Germany; ^4^ Department of Chemistry and Materials Science Aalto University Aalto FI‐00076 Finland

**Keywords:** Energy transfer, NIR luminescence, Organic phosphorescence, Sensitization, Through‐space interactions

## Abstract

A family of coordination compounds with short intramolecular spatial separation between an organic chromophore and a metal center was studied. The specific geometry was realized by means of anthracene‐functionalized tertiary aryl phosphanes. Their silver and gold complexes (**1**, **2**) operate as conventional fluorophores, with photophysical behavior defined by anthracene‐localized allowed transitions. In contrast, bichromophoric species, containing phenyl bipyridine‐ (**3**, **5**, **6**, **8**) or terpyridine‐ (**4**, **7**) derived platinum(II) fragments, demonstrate fast intersystem crossing to the triplet state associated with the pincer metal component. Theoretical results corroborated that the short intramolecular distance between the platinum constituent and the adjacent anthracene facilitates subsequent through‐space triplet (*T*_2_, pincer fragment)→triplet (*T*_1_, anthracene) energy transfer. This process occurs at a rate of ∼10^11^ s^−1^, surpassing the rates of *T*_2_→S_0_ relaxation. This prevents visible phosphorescence from the platinum(II) motifs but enables near‐IR organic phosphorescence in the solid state, including dyes with very inefficient intersystem‐crossing (ISC). Thus, the composite molecules **3**–**8** illustrate a feasible approach to the tunable sensitization of organic dyes and the design of low‐energy triplet emitters.

## Graphical abstract

Intramolecular energy transfer in bichromophore molecules, regulated by the spatial separation between organic and metal‐containing components, allows to attain room temperature near‐IR phosphorescence of polyaromatic dyes, including those with very low intersystem crossing rates, by using conventional organometallic fragments as sensitizers.

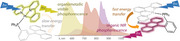

## Introduction

The development of near‐infrared (NIR) emitters with luminescence wavelengths beyond 700 nm is driven by their applications in several areas, including electroluminescent devices, therapy, diagnosis and healthcare techniques, bioimaging, and sensing.^[^ ^1^, ^2^, ^3^, ^4^, ^5^, ^6^ ^]^ Construction of organic NIR dyes primarily relies on extending the π‐conjugated system and its decoration with electron‐donating and accepting groups. This molecular architecture favors intramolecular charge transfer (ICT), which reduces the optical gap and lowers the energies of absorption and fluorescence. Combining organic chromophores with transition metal ions offers another pathway to tune their electronic properties.^[^ ^5^, ^7^, ^8^, ^9^, ^10^ ^]^ The presence of a heavy element can enhance spin‐orbit coupling (SOC) between the singlet and triplet excited states, leading to fast ISC and efficient population of the lower‐lying triplet state (denoted as *T*_1_). With the help of a heavy atom inducing substantial SOC, the triplet wavefunction acquires a fraction of the singlet character, allowing radiative relaxation from *T*_1_ to the ground state S_0_ via partially spin‐allowed phosphorescence. Such luminescence typically exhibits lifetimes falling within the microsecond range.^[^ ^8^, ^11^, ^12^ ^]^

Manipulations with metal ions and organic parts diversify possible electronic transitions responsible for optical properties. The excited‐state dynamics of metal compounds can involve metal‐to‐ligand (ML), ligand‐to‐metal (LM), ligand‐to‐ligand (LL’), and intraligand (IL) charge transfer (CT) states (MLCT, LMCT, LL'CT, and ILCT), which potentially offer new photophysical features compared to those of organic fluorophores, including efficient ISC and NIR triplet photoluminescence.^[^ ^5^, ^13^ ^]^ For example, the coordination of a diimine fused with perylene bisimide to the ruthenium(II) center produces a metal complex showing phosphorescence with a peak maximum at 780 nm and remarkable quantum yield (*Φ*_em_) of 0.11 in solution.^[^ ^14^ ^]^ The analogous complex of iridium(III) exhibited NIR emission (*λ*_max_ ≈ 750 nm) in the solid state and concentration‐dependent dual fluorescence‐phosphorescence in solution.^[^ ^15^ ^]^ The emission from triplet states possessing mixed (ML + IL)CT nature maximized at 824 nm (*Φ*_em_ = 0.05 in polymer film) was reported for a homoleptic Ir(III) complex containing metalated (thiophenyl)‐benzophthalazine ligands with extended π‐conjugation.^[^ ^16^ ^]^ Double cycloplatination of di(isoquinolinyl)pyrene^[^ ^17^ ^]^ and bis(dithienyl)‐pyrimidine^[^ ^18^ ^]^ ligands afforded bimetallic compounds displaying predominantly intraligand triplet luminescence at 704 nm (*Φ*_em_ = 0.17 in CH_2_Cl_2_) and 725 nm (*Φ*_em_ = 0.25 in toluene), respectively.

**Figure 1:**
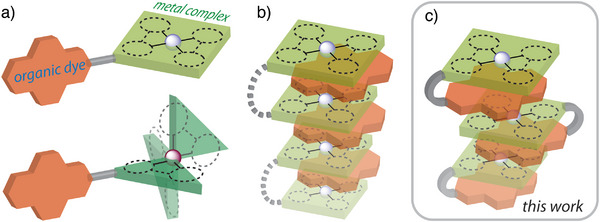
Schematic representation for the combination of organic dyes and metal complexes: a) spatially separated covalently bound dyads;^[^ ^10^, ^19^, ^20^, ^21^, ^22^ ^]^ b) noncovalently assembled systems with through‐space interaction;^[^ ^23^, ^24^, ^25^ ^]^ c) covalently assembled systems with through‐space interaction (this work).

Incorporation of a transition metal directly into a dye structure is often a nontrivial synthetic task. In the case of a metal complex bearing a pendant organic dye (Figure 1a), the distance as well as the connectivity between the chromophore core and the metal center play a crucial role in the population and relaxation of the lowest energy triplet state. Increased separation between the heavy metal and organic chromophore induces alterations in photophysical properties, potentially leading to phenomena such as intraligand fluorescence, dual emission, or negligible luminescence attributed to the nonradiative relaxation of the long‐lived ^3^IL state.^[^ ^8^, ^20^, ^21^, ^22^ ^]^ The latter feature finds applications in upconversion, photodynamic, and photothermal therapy.^[^ ^26^, ^27^, ^28^, ^29^, ^30^, ^31^ ^]^ In particular, the combination of mercaptopyrene and boron‐dipyrromethene (bodipy) ligands coordinated to Pt(II) led to charge‐separated ^3^(pyrene→bodipy) CT state and intense phosphorescence (*λ* = 724 nm, *Φ*_em_ = 0.15).^[^ ^32^ ^] 3^IL luminescence of the bodipy dye in the NIR region (*λ* = 770 nm) mixed with ^3^MLCT emission (*λ* = 660 nm, total *Φ*_em_ = 0.035) was realized in its diplatinum derivative, where two cyclometalated fragments are attached to the dye core through acetylene linkers.^[^ ^19^ ^]^ Appending the bodipy motif to the bipyridine ligand of a Ru(II) complex generated either weak bodipy‐based phosphorescence at 741 nm, or residual fluorescence of this dye, depending on the connection mode.^[^ ^33^ ^]^ An opened form of an acetylene functionalized rhodamine‐type ligand attached to a platinum(II) moiety exhibited room‐temperature NIR phosphorescence (*λ* = 740 nm) and delayed fluorescence (*λ* = 620 nm) originating from the organic component.^[^ ^34^ ^]^ Furthermore, the Pt(bipyridine) unit induced only faint phosphorescence in acetylene–naphthalene bisimide (*λ* = 784 nm, *Φ*_em_ = 0.002 in toluene),^[^ ^35^ ^]^ whereas an acetylene–perylene bisimide bound to a gold(III) cyclometalated unit remains fluorescent.^[^ ^36^ ^]^

An interesting feature of square planar platinum(II) complexes is their tendency for intermolecular self‐assembly via π–π stacking and metallophilic Pt^…^Pt interactions. The luminescence in these aggregates originates from metal‐to‐metal‐to‐ligand charge transfer (^3^MMLCT) states, and is considerably lower in energy than that of discrete constituents, shifting the wavelength maximum up to 1300 nm.^[^ ^37^ ^]^ Notably, planar Pt(II)‐containing fragments can interact noncovalently not only with each other but also with π‐conjugated molecules (Figure 1b).^[^ ^23^, ^24^, ^38^ ^]^ Once they are in close proximity, electronic coupling might impact the optical properties of these systems. Indeed, it has been shown recently that cocrystals of Pt(acetylacetonate)_2_ and naphthalene diimides demonstrated successful sensitization of organic phosphorescence by means of intermolecular metal‐to‐dye charge transfer.^[^ ^25^ ^]^ However, the photophysical characteristics of such intermolecular associates should depend on crystal packing, which is difficult to predict and control.

**Scheme 1:**
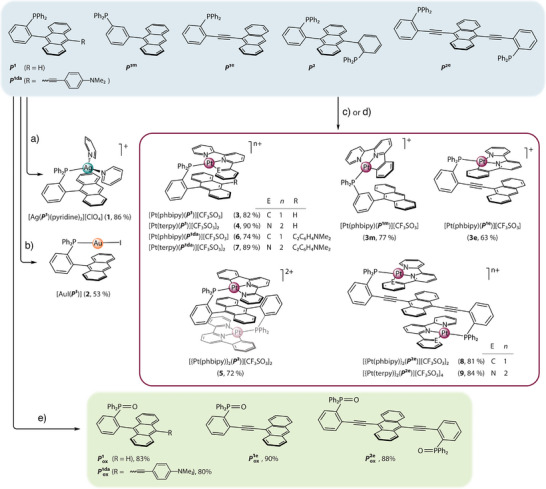
Synthesis of complexes **1**–**9**, **3 m** , **3e** (all reactions were carried out at room temperature under a nitrogen atmosphere) and phosphane oxides, ***P* ^1^_ox_**, ***P* ^1da^_ox_**, ***P* ^1e^_ox_**, ***P* ^2^_ox_**: a) AgClO_4_, pyridine, CH_2_Cl_2_; b) AuI, CH_2_Cl_2_; c) for **3**, **3 m** , **3e**, **5**, **6**, **8** [Pt(phbipy)Cl], AgCF_3_SO_3_, CH_2_Cl_2_/acetonitrile/methanol; d) for **4**, **7**, **9** [Pt(terpy)(acetonitrile)](CF_3_SO_3_)_2_, CH_2_Cl_2_; e) aqueous H_2_O_2_, CH_2_Cl_2_.

In this work, we probed the possibility of intramolecular through‐space triplet energy transfer within a covalently bound molecular entity (Figure 1c) to achieve a prominent NIR emission. For the proof of concept, we utilized anthracene‐containing phosphane ligands, the configuration of which facilitates short contacts between the coordinated metal center and the organic chromophore (Scheme 1). Additionally, the chosen stereochemistry favors embedding the PAH fragment between two metal units, tailoring the electron donor functionality and the length of the conjugated system to reduce the optical bandgap, while the coordinating phosphane groups can bind a range of transition metal complexes. As a result, providing a short through‐space separation between the metal center and a fluorescent dye can induce efficient triplet (metal‐centered moiety)‐triplet (organic motif) energy transfer, followed by the radiative relaxation of an organic unit‐localized state. With this approach, we successfully demonstrate organic NIR phosphorescence by using conventional organometallic fragments.

## Results and Discussion

### Synthesis and Characterization

*Ortho*‐phenyl substituted tertiary phosphanes, (2‐(anthracen‐9‐yl)phenyl)diphenylphosphane (***P* ^1^**), 4‐((10‐(2‐(diphenylphosphanyl)phenyl)anthracen‐9‐yl)ethynyl)‐N,N‐dimethylaniline (***P* ^1da^**), 9,10‐bis(2‐(diphenylphosphanyl)phenyl)anthracene (***P* ^2^**) (Scheme 1) were prepared as described earlier.^[^ ^39^, ^40^ ^]^ The syntheses of (3‐(anthracen‐9‐yl)phenyl)diphenylphosphane (***P* ^1m^**), (2‐(anthracen‐9‐ylethynyl)phenyl)diphenylphosphane (***P* ^1e^**), and 9,10‐bis((2‐(diphenylphosphanyl)phenyl)ethynyl)anthracene (***P* ^2e^**) were carried out analogously and details are given in the Supporting Information (ESI). The titled phosphane ligands were reacted with AgClO_4_, AuI, [Pt(phbipy)(NCMe)][CF_3_SO_3_] (Hphbipy = 6‐phenyl‐2,2′‐bipyridine), and [Pt(terpy)(NCMe)][CF_3_SO_3_]_2_ (terpy = 2,2′;6′,2″‐terpyridine) precursors to afford complexes [Ag(***P* ^1^**)(pyridine)_2_][ClO_4_] (**1**), AuI(***P* ^1^**) (**2**), [Pt(phbipy)(***P* ^1^**/***P* ^1m^**/***P* ^1e^**/***P* ^1da^**)][CF_3_SO_3_] (**3**/**3 m**/**3e**/**6**), [Pt(terpy)(***P* ^1^**/***P* ^1da^**)][CF_3_SO_3_]_2_ (**4**/**7**), [{Pt(phbipy)}_2_(***P* ^2^**/***P* ^2e^**)][CF_3_SO_3_]_2_ (**5**/**8**), and [{Pt(terpy)}_2_(***P* ^2e^**)][CF_3_SO_3_]_4_ (**9**) in moderate to good yields as crystalline solids (Scheme 1, see the ESI for preparation protocols). The phosphanes with *meta*‐phenylene (***P* ^1m^**) and *ortho*‐phenylene‐ethynyl (***P* ^1e^**/***P* ^2e^**) spacers were employed to compare the effect of intramolecular interfragment separations in the corresponding metal complexes on their photophysical properties. The phosphane oxides ***P* ^1^_ox_**, ***P* ^1da^_ox_**, ***P* ^1e^_ox_**, ***P* ^2^_ox_**,^[^ ^40^ ^]^ and ***P* ^2e^_ox_** as reference fluorophores were obtained by treating the parent ligands with aqueous hydrogen peroxide (Scheme 1).

**Figure 2:**
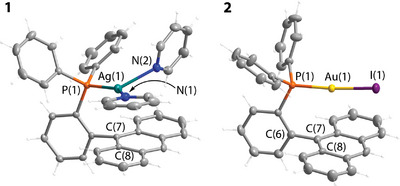
Molecular structures of complexes **1** and **2** (two independent molecules are found in the unit cell of **2**; displacement ellipsoids are shown at the 50% probability level; counterion for **1** is omitted for clarity).

**Figure 3:**
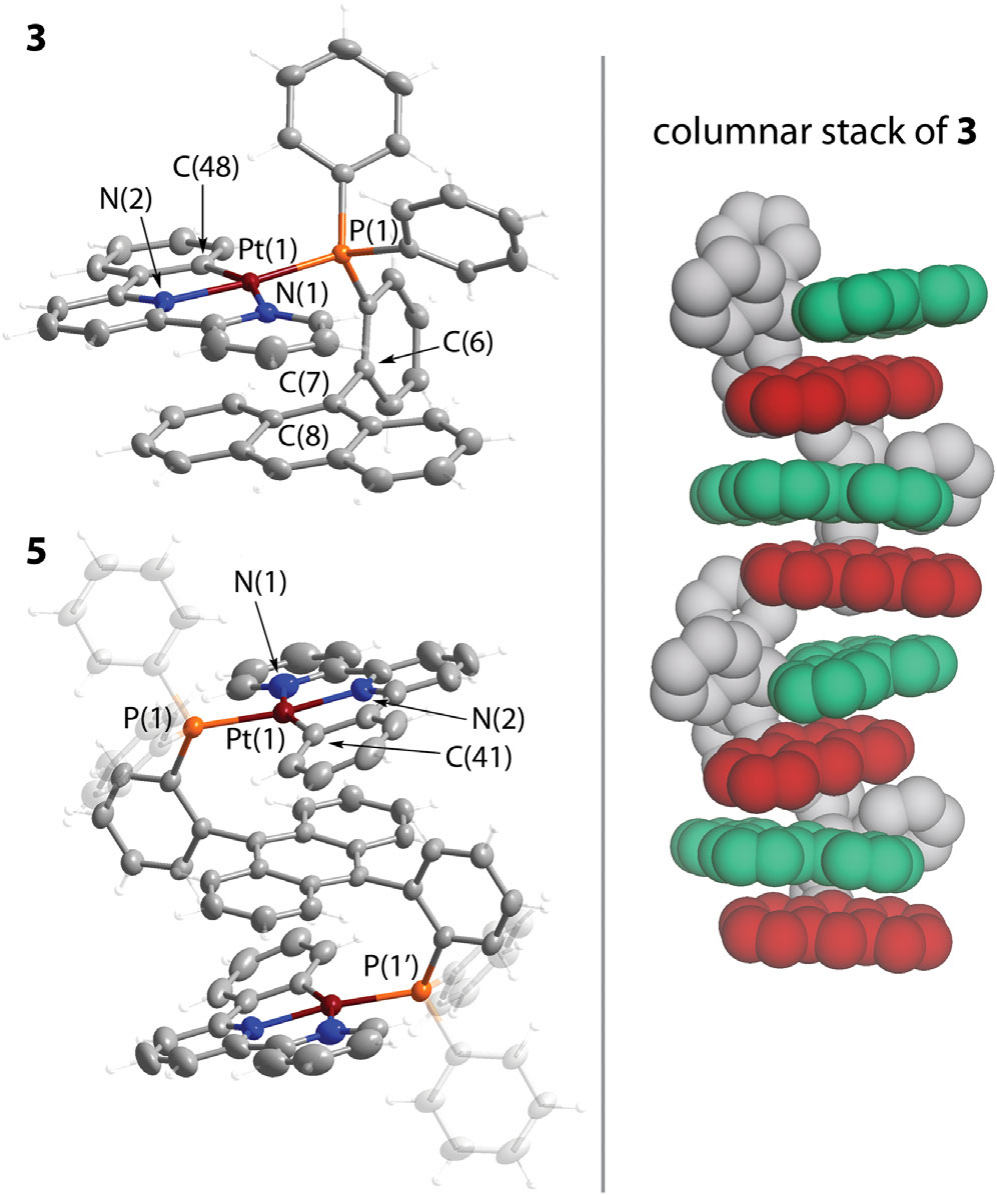
Molecular structures of complexes **3** and **5** (left) and the intermolecular stack of **3** (right, anthracene and platinum pincer fragments are shown in red and green, respectively; two independent molecules are found in the unit cell of **5**; displacement ellipsoids are shown at the 50% probability level; counterions are omitted for clarity).

**Figure 4:**
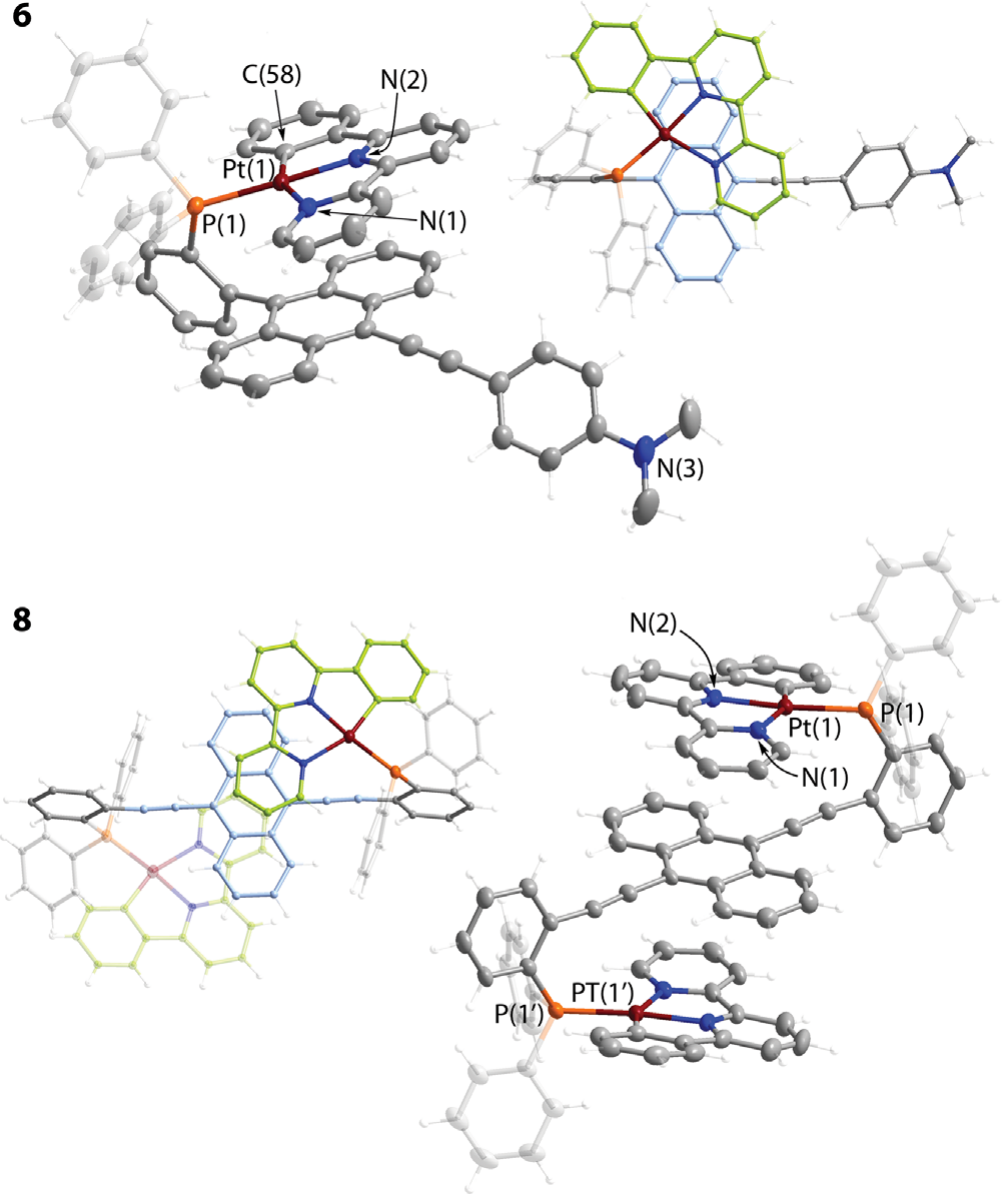
Molecular structures of complexes **6** and **8** (two independent molecules are found in the unit cell of **6**; displacement ellipsoids are shown at the 50% probability level; counterions are omitted for clarity).

The molecular structures of the ligand ***P* ^1m^**, the oxide ***P* ^1e^_ox,_** and all complexes (**1**–**9**, **3 m** , **3e**) were determined by single crystal X‐ray diffraction analysis^[^ ^41^ ^]^ and are depicted in Figures 2, 3, 4 and Figures S1–S5, crystal data and selected parameters are listed in Tables S1–S6. In compounds **1**–**7** having *ortho*‐phenylene substituted phosphanes and **3e**, **8**, **9** with phenylene‐ethynyl spacers, the metal fragments are placed over the anthracene and acetylene systems, respectively, to give relatively short M^…^π contacts. For **1** and **2**, the distances Ag^…^C_anth_(7) (3.102(2) Å) and Au^…^C_anth_(7) (3.149(5)/3.098(5) Å, two values are for independent molecules found in the unit cell) are substantially shorter than the corresponding sum of van der Waals radii (3.42 Å for Ag─C and 3.36 Å for Au─C pairs).^[^ ^42^ ^]^ The silver ion is found in a distorted trigonal environment completed by phosphorus and nitrogen donors. The Ag(1)─N(2) bond length (2.391(2) Å) is longer than Ag(1)─N(1) (2.218(2) Å), which might account for weaker binding of the former pyridine ligand, compensated by the Ag^…^C═C interaction. The P─Au─I fragment in **2** is nearly linear (175.24(3)°/176.28(3)°) and is located symmetrically over the central ring of the anthracene moiety; the torsional angle Au(1)‐P(1)‐C(6)‐C(7) is less than 2° (Figure 2). These configurations of **1** and **2** are similar to those of structurally analyzed silver(I)^[^ ^43^ ^]^ and gold(I)^[^ ^44^, ^45^, ^46^, ^47^ ^]^ complexes bearing R_2_P(biaryl) phosphanes.

The conformations of platinum derivatives **3**–**7** in the solid state depend on both the phosphane and pincer ligands. ***P* ^1^**, ***P* ^1da^**, and ***P* ^2^**‐based complexes **3**, **5,** and **6** reveal considerable twisting of the planar {Pt(phbipy)} motif relative to the anthracene system with torsional angles Pt(1)‐P(1)‐C(6)‐C(7) of 36.3(1)° for **3**, 35.9(2)° for **5** (Figure 3), and 39.3(1)/40.8(1)° for **6** (two independent molecules, Figure 4).

This molecular arrangement, where the bipyridine fragment is preferably placed over the polyaromatic moiety, is probably driven by a less favorable interaction of the electron‐rich metalated phenylene ring with the π‐system of anthracene. For terpyridine compounds **4** and **7**, no such distortion is observed, the Pt(1)‐P(1)‐C(6)‐C(7) angles are 4.7(1)°/7.5(1)° for **4** (two independent molecules) and 1.3(1)° for **7** (Figures S1 and S3), implying a more even interaction of the electronic clouds of {Pt(terpy)} and anthracene units. On the other hand, the ***P* ^1e^** and ***P* ^2e^**‐derived compounds **8** and **9** (Figure 4 and Figure S5) reveal no difference between pincer ligands and adopt distorted configurations with torsional angles Pt(1)‐P(1)‐C(6)‐C(7), similar to those in **3** and **6** (see above). The structural features of the platinum(II) complexes were computationally well reproduced, as illustrated by the comparison of experimental and optimized ground‐state geometries (Figures S6 and S7). The shortest intramolecular noncovalent Pt^…^C_anth_ contacts for **3**–**7** and Pt^…^C_ethynyl_ for **3e**, **8**, **9** fall in narrow ranges 3.162(3)–3.268(3) and 3.179(3)–3.243(3) Å, respectively (cf. the sum of van der Waals radii is 3.45 Å), which are somewhat below the values predicted theoretically (3.246–3.489 and 3.306–3.349 Å). Complex **3 m** crystallized in two modifications, minor CH_2_Cl_2_ solvate (*P* $\bar{1}$ space group) and major disordered ill‐defined solvate (*C*2/*c* space group, Scheme 1 and Figure S2). In both cases, the metal center and anthracene motif are expectedly more separated than those in **3**–**7**. The shortest Pt^…^C_anth_ distance is increased to 5.239(2) and 5.477(6) Å for *P* $\bar{1}$ and *C*2/*c* solvatomorphs, respectively. The crystal packing in **3 m** (*P* $\bar{1}$) provides a shorter intermolecular distance Pt(1)^…^C_anth_(14) of 3.743(2) Å, which is not found in **3 m** (*C*2/*c*), Figure S2.

The ^31^P{^1^H} and ^1^H NMR spectra of **1**–**9**, **3 m** and **3e** confirm the coordination of phosphanes to the metal centers and retaining of molecular structures in solution. The phosphorus signals of complexes, found in the range of *δ* 6.8–31.9 ppm, demonstrate characteristic low field shifts with respect to the parent ligands (*δ* = −14.7 to −4.5 ppm for employed phosphanes), along with characteristic coupling to ^107/109^Ag (**1**: *J*_109AgP_ = 752 Hz, *J*_107AgP_ = 651 Hz) and ^195^Pt nuclei (*J*_PPt_ = 3642–4112 Hz, Figures S8–S18). The observed patterns of proton resonances also correspond to the presence of molecular species. Visible broadening of some ^1^H signals in the case of complex **1** can be attributed to the lability of pyridine ligands, while for Pt(phbipy) complexes (**3**, **3e**, **5**–**8**), it plausibly originates from asymmetric interaction of the metal fragment with the anthracene motif (Figures S6 and S7), which could lead to scissor‐like intramolecular dynamics. No appreciable concentration‐dependent aggregation, which is often encountered for square‐planar platinum(II)‐based coordination compounds,^[^ ^48^ ^]^ was found for **3**–**9**, **3 m,** and **3e**, due to the bulky tertiary phosphane ligands.

### Photophysical Properties

**Figure 5:**
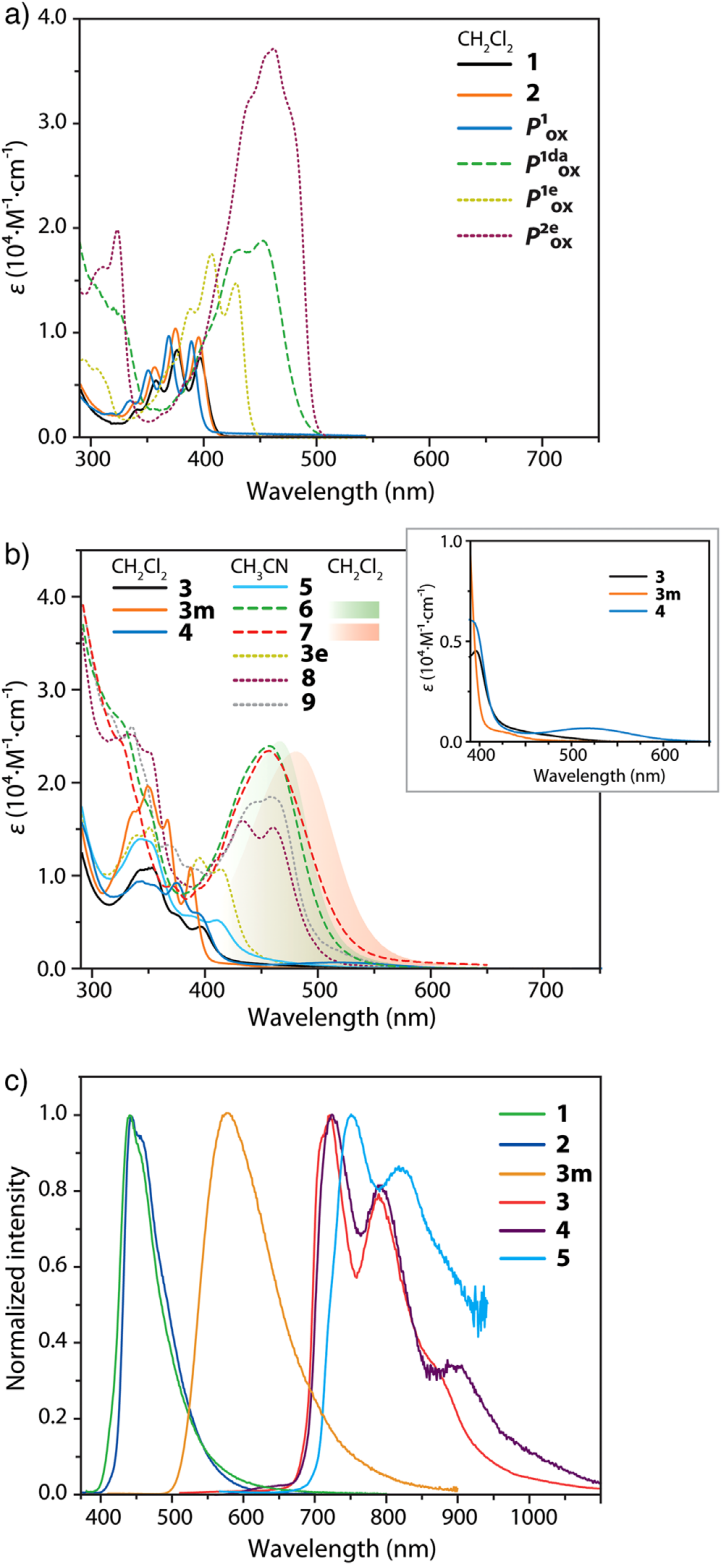
UV–vis absorption spectra of a) **1**, **2**, ***P* ^1^_ox_**, ***P* ^1da^_ox_**, ***P* ^1e^_ox_**, and ***P* ^2e^_ox_** in CH_2_Cl_2_, b) **3**, **4**, **3 m** in CH_2_Cl_2_ and **5**–**9**, **3e** in acetonitrile (gradient filled profiles show the low energy bands for **6** and **7** in CH_2_Cl_2_, inset zooms spectra of **3**, **4**, **3 m** in the region 390–600 nm), and c) normalized emission spectra of crystalline complexes **1**–**5** and **3m**(*C*2/*c*) at 298 K (*λ*_ex_ = 380 nm for **1**–**5** and *λ*_ex_ = 360 nm for **3 m** ).

Figure 5 shows the absorption spectra of complexes **1**–**9**, **3 m**, and **3e**, as well as of ***P* ^1^_ox_**, ***P* ^1da^_ox_**, ***P* ^1e^_ox_**, and ***P* ^2e^_ox_** in solution. Pertinent photophysical data for the titled species are listed in Table 1 and Tables S7, and S8. For all compounds, the spectra show strong absorption bands below 300 nm (not shown here), dominated by the phosphane ligands. The comparable intensity around 350 nm for **3**–**9**, **3 m** , and **3e** is assigned to transitions into excited phbipy‐ and terpy‐centered intraligand π–π* (^1^IL) configurations, analogous to related platinum complexes.^[^ ^49^, ^50^, ^51^ ^]^ The vibronic absorption bands between 370‐400 nm for compounds **1**–**5** and **3 m** (***P* ^1^**, ***P* ^1m^**, ***P* ^2^** derivatives), which extend to ca. 470 nm for **3e**, **8**, **9** (complexes of ***P* ^1e^**, ***P* ^2e^**), closely resemble those found in the corresponding ligand oxides ***P* ^1^_ox_**/***P* ^2^_ox_** ^[^ ^40^ ^]^ and ***P* ^1^_ox_**/***P* ^2e^_ox_**, respectively.

**Table 1:** UV–vis absorption (solution) and photoluminescence (solid‐state) properties of complexes **1**–**9**, **3 m** , **3e** at room temperature.

	*λ*_abs_, nm (*ε*, 10^‒3^ M^‒1^ cm^‒1^)	*λ*_em_, nm^c)^	*τ*_av_, µs^c)^, ^d)^
**1**	257 (83.7), 358 (5.2), 377 (8.1), 397 (7.4)^a)^	441	(2.05 ± 0.01).10^−3^
**2**	258 (108.5), 340 (3.1), 357 (6.4), 375 (10.2), 396 (9.2)^a)^	442	(0.69 ± 0.01) .10^−3^
**3**	255 (76.6), 353 (13.2), 396 (5.4)^a)^	724, 802, ca. 900	35.50 ± 0.04
**3m**	257 (141.0), 333 (16.3), 350 (19.7), 367 (16.7), 387 (11.3)^a)^	576	1.27 ± 0.02
**4**	246 (80.4), 341 (9.5), 374 (8.8), 391 (6.0), 518 (0.5)^a)^	723, 796, ca. 900	13.80 ± 0.05
**5**	256 (50.0), 343 (14.0), 387 (5.7), 411 (5.2)^b)^	750	8.4 ± 0.1
**6**	255 (71.4), 328 sh (26.7), 352 sh (16.9), 457 (24.1)^b)^	640, 843	2.74 ± 0.03
**7**	252 (73.2), 326 sh (24.2), 372 (8.9), 455 (23.4)^b)^	588, ca. 890	n.d.
**3e**	259 (94.5), 338 (14.3), 352 (15.1), 396 (11.9), 415 (10.7)^b)^	753	n.d.
**8**	252 (103.0), 335 (25.2), 352 (23.3), 411 (11.8), 433 (15.9), 460 (15.2)^b)^	638, 842	5.0 ± 0.3
**9**	267 (84.0), 318 (27.0), 336 (26.0), 372 (13.1), 444 (17.9), 462 (18.4)^b)^	n.d.	n.d.

^a)^ CH_2_Cl_2_

^b)^ Acetonitrile

^c)^ Solid state

^d)^ Amplitude‐weighted average lifetimes determined by the equation *τ*_av_ = Σ*A_i_τ_i_*, *A_i_* = weight of the *i*th component.

These bands originate from electronic transitions into an excited π–π* state involving the anthracene and (di)ethynyl‐anthracene chromophores. Distinct from phosphane oxides, weak new absorption bands appear, extending to 600 nm for **3**–**5**, **3 m** , and **3e**, **8**, **9**. These transitions are mainly attributed to metal(Pt(II))‐to‐ligand (phbipy or terpy) charge transfer (MLCT) states, together with a close interplay between the Pt(phbipy/terpy) and anthracene motifs. This assignment is supported by the observation that **3 m** exhibits notably weaker absorption in the range 420–600 nm than that of **3**. Moreover, a broad band around 518 nm found for **4** (Figure 5b) is not observed for other congeners [Pt(terpy)(phosphane)]^2+^, which do not have a polyaromatic moiety.^[^ ^52^ ^]^ Both observations indicate that the new, low‐energy absorption bands should arise, in part, from the through‐space interaction between Pt‐(phbipy/terpy) and (ethynyl)‐anthracene, which are absent when the Pt^…^anthracene distance in **3 m** is elongated (cf. **3** and **3e**).

The intensely colored compounds **6** and **7** exhibit low‐energy absorption bands peaking at 455–457 nm in acetonitrile (Figure 5b). Analogous to the corresponding oxide ***P* ^1da^_ox_**, these bands are attributed to intraligand charge transfer (ILCT) states within 4‐(anthracen‐9‐ylethynyl)‐N,N‐dimethylaniline (see Scheme 1), which is likely further enhanced by metal(Pt)–π interactions. These viewpoints are further supported by the computational approaches (vide infra).

**Figure 6:**
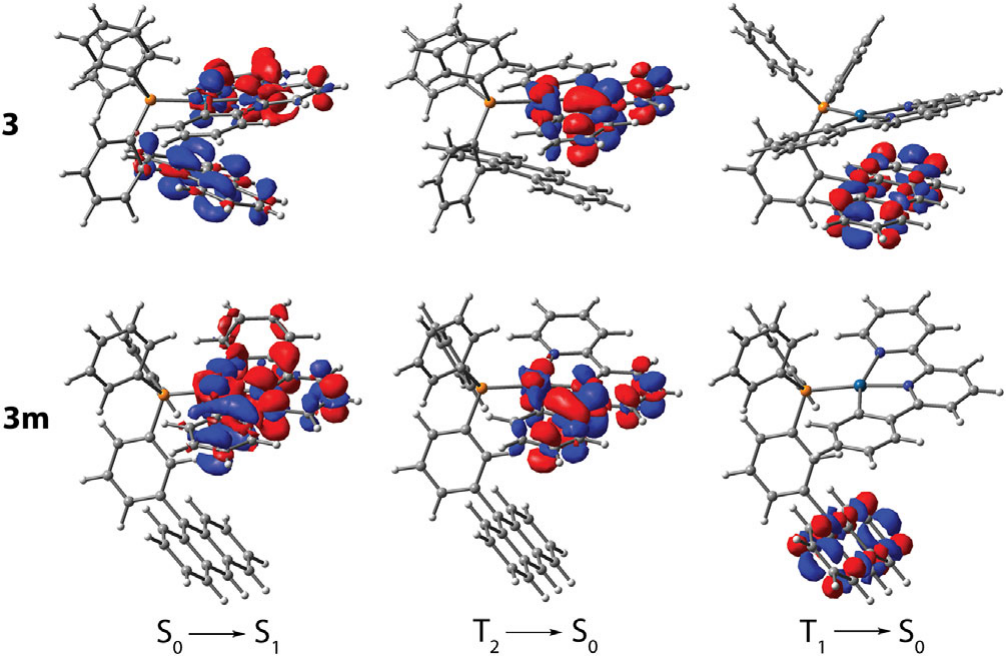
Electron density difference plots for complexes **3** (top) and **3 m** (bottom) (isovalue 0.001 a.u.). During the electronic transition, the electron density decreases in the blue areas and increases in the red areas. Note the calculated energy level is in the order of *S*_1_ > *T*_2_ > *T_1_* (Table S10).

**Figure 7:**
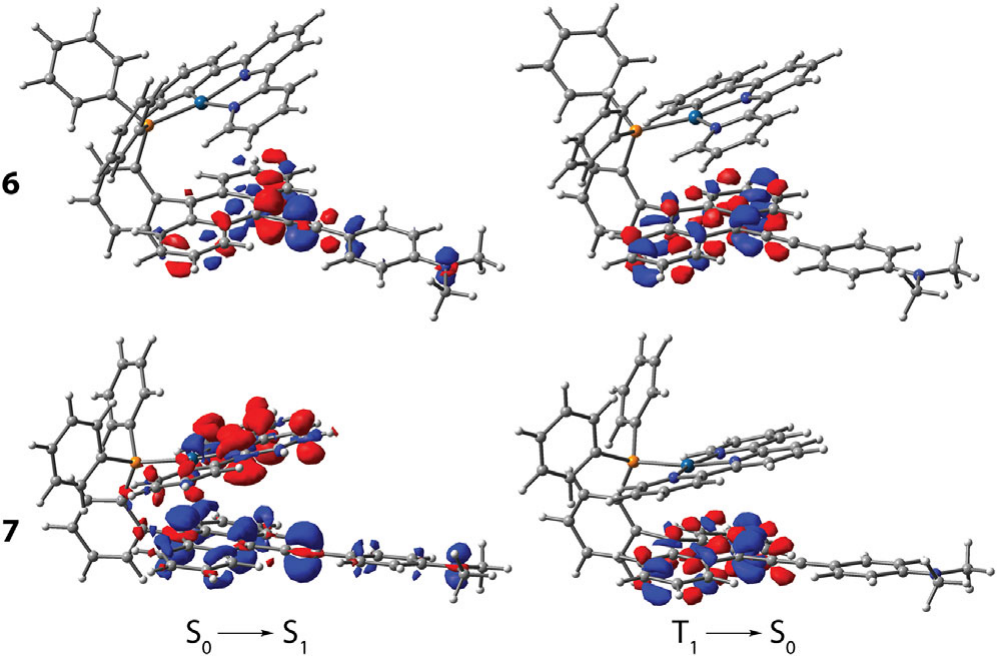
Electron density difference plots for complexes **6** (top) and **7** (bottom) (isovalue 0.001 a.u.). During the electronic transition, the electron density decreases in the blue areas and increases in the red areas.

Time‐dependent density functional theory (TD‐DFT) analysis confirms the π–π*(anthracene) character of the *S*_1_ state in **1** and **2** (Table S9, Figure S19). For **3**–**5**, the computed *S*_1_ state involves Pt→phbipy/terpy charge‐transfer excitations, i.e., MLCT mixed with π–π* and through‐space ligand‐to‐ligand charge transfer (LL'CT, anthracene→bi/terpy, Table S9) character for **3**–**5** (Figure 6 and Figure S20). In contrast, the *S*_1_ state of **3 m** is dominated by the Pt→phbipy excitation (MLCT character, Figure 6). The absence of LL'CT states engaging anthracene indicates the lack of interaction between Pt(phbipy) and anthracene, consistent with the much weaker 420–600 nm absorption in **3 m** (cf. **3**–**5**) (Figure 5b). For **6**, the *S*_1_ state is primarily attributed to the intraligand charge transfer (ILCT) configuration, implying a donor–acceptor in the ***P* ^1da^_ox_** moiety (Figure 7). As for **7**, in addition to the same ILCT signature observed in **6**, there is also a significant contribution of the LL'CT state (anthracene→terpy, Table S9). This difference is due to the increase of electron deficiency caused by the additional pyridine group in **7**, which enhances the electron‐withdrawing nature of the pincer ligand.

**Figure 8:**
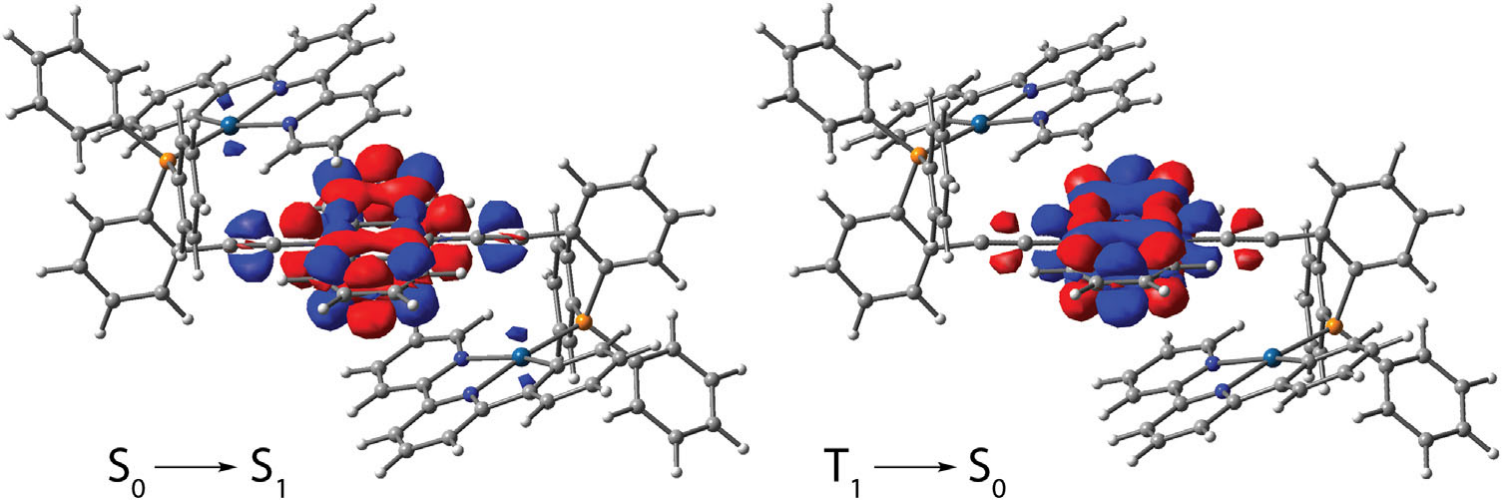
Electron density difference plots for complex **8** (isovalue 0.001 a.u.). During the electronic transition, the electron density decreases in the blue areas and increases in the red areas.

As a result, the electronic structure of complex **7** becomes more delocalized compared to **6**, which, in turn, lowers the lowest‐lying absorption energy. This rationalizes the intense absorption band around 455–480 nm for **6** and **7** (*ε* > 2 × 10^4^ m ^−1^ cm^−1^, Figure 5b), where **1**–**5** exhibit a much weaker absorption in the same region (*ε* < 10^3^ m ^−1^ cm^−1^). The lowest energy excitations in complexes **3e**, **8**, and **9** occur primarily within (di)ethynyl‐anthracene motifs with some charge transfer from Pt(II) centers (Figure 8 and Figure S21). Decoration of the PAH with acetylenic C_2_ groups extends conjugation and gradually redshifts the experimental absorption wavelengths (e.g., *λ*_abs_ **3** < **3e** < **8**), which is well confirmed computationally (Table S9). It is worth noting that the calculated *S*_0_–*S*_1_ gaps are underestimated compared to experimental values. This discrepancy may stem from the neglected interactions between cationic molecules and their counter anions, as well as the oversimplified treatment of through‐space charge transfer.

Complexes **3**–**9**, **3 m**, and **3e** are very weakly emissive in solution at room temperature even under deoxygenated conditions. This is plausibly due to the formation of an anthracene‐localized triplet state (vide infra), which primarily undergoes nonradiative relaxation in the liquid medium. Additionally, **3**–**9** demonstrate limited photostability at low concentrations in solution, particularly in the presence of oxygen (Figure S22), which accelerates the disappearance of the low energy band. We tentatively attribute this behavior to the degradation of polyaromatic phosphane ligands presumably due to the photoreactivity of anthracene.^[^ ^53^ ^]^ This might include dimerization,^[^ ^54^ ^]^ endoperoxide formation,^[^ ^55^ ^]^ or dissociation and subsequent oxidation.^[^ ^56^ ^]^

Different from their behavior in solution, complexes, **1**–**9**, **3 m**, and **3e** are stable in the solid state, showing no decomposition during measurements and storage. Therefore, the ensuing discussion of **1**–**9**, **3 m** , and **3e** is focused on their solid‐state photoemission properties. Crystalline complexes **1** and **2** are moderately intense blue fluorophores under ambient conditions (see Table 1), with emission maxima around 440 nm (Figure 5c). The observed lifetimes of 2.05 ns (**1**) and 0.69 ns (**2**) suggest prompt anthracene‐centered fluorescence, which is almost unaffected at 77 K (Figure S23 and Table S7). The given values correlate with the characteristics of phosphane oxide ***P* ^1^_ox_** (Figure S24 and Table S8), which shows featureless emission at 446 nm (*τ*_obs_ = 6.26 ns) in the solid state at 298 K. These results are also consistent with the predicted radiative transitions *S*_1_→*S*_0_ for **1** (424 nm) and **2** (409 nm, see Figure S19 and Table S11).

In stark contrast to the predominant blue fluorescence observed for silver and gold compounds **1** and **2** both at 298 and 77 K, the platinum(II) complexes **3** and **4** exhibit prominent NIR emission in the solid state with maxima at 724 and 723 nm, respectively, and the vibronic progressions extending beyond 900 nm (Figure 5c and Table 1). Placing anthracene in between two cyclometalated units in **5** shifts the maximum to 750 nm. The emission lifetimes of 35.5 µs (**3**), 13.8 µs (**4**), and 8.4 µs (**5**), elongated to 14.2–50.2 µs at 77 K (Table S7), suggest the involvement of a triplet state, closely resembling the spectral profile and vibronic peaks characteristic of anthracene, thereby confirming their assignment to the phosphorescence of the polyaromatic motif. Cooling **3**–**5** to 77 K narrows the bands and slightly blue shifts the maxima for **3** and **4**, whereas it has a minimal influence on the spectroscopic profile of **5** (Figure S25).

Further validation of this assignment comes from the TD‐DFT calculations, which attribute the *S*_1_ state to a combination of MLCT (Pt→phbipy/terpy) and anthracene→bi/terpy (LL'CT) or π–π*(anthracene) character for (**3**, **4**) and **5**, respectively (see Figure 6, Figure S20, and Table S9). Despite the participation of the metal fragment to the frontier orbitals in the description of the *S*_1_ state, the lowest triplet state *T*_1_ is exclusively associated with excitations involving the anthracene moiety for **3**–**5** (Figure 6 and Figure S20), supporting their phosphorescent nature.

On the contrary, the steady‐state emission spectrum of crystalline **3 m** (*C*2/*c*) is markedly different (Figure 5c) and displays a broad and structureless band centered around 576 nm, which is accompanied by a barely distinguishable shoulder around 700 nm, somewhat enhanced under cryogenic conditions (Figure S25) and resembling the phosphorescence of anthracene (cf. **3**–**5** in Figure 5c). The emission lifetime monitored at 575 nm was 1.27 µs (Figure S26). This behavior is reminiscent of its congener, the [Pt(phbipy)(PPh_3_)][ClO_4_] complex,^[^ ^49^, ^50^ ^]^ indicating a predominant ^3^MLCT character.

Monitoring the emission at 780 nm of **3 m** reveals besides a lifetime component from the tail of the 575 nm emission, a long‐lived decay up to 82 µs (see Figure S27), which is reasonably assigned to the contribution of anthracene phosphorescence. TD‐DFT calculations indicate that the frontier orbitals of the *T*_1_ state for both **3 m** and **3** involve local excitation of anthracene, while *T*_2_ is ascribed to ^3^MLCT character associated with the Pt(phbipy) complex (Figure 6). Moreover, *T*_1_ and *T*_2_ are calculated to be lower in energy than the *S*_1_ state for both **3** and **3 m** (Table S10). Thus, we conclude that **3** exhibits *T*_1_ anthracene‐related phosphorescence, whereas **3 m** gives dual phosphorescence originating from *T*_2_ with ^3^MLCT character (major) and anthracene‐centered *T*_1_ state (minor). The distinction between **3** and **3 m** in the origin of their triplet emission is likely governed by the rate of *T*_2_→*T*_1_ energy transfer, which is of significant fundamental interest. Importantly, due to the spatial separation of electron densities between *T*_2_ and *T*_1_, the relaxation *T*_2_→*T*_1_ cannot be governed by strong exciton‐vibration coupling (internal conversion). Instead, the relaxation process in **3** and **3 m** as composite bichromophore molecules likely involves energy transfer such as a Dexter‐type intramolecular process involving an electron‐exchange mechanism.

The above viewpoint stimulated us to probe the relaxation dynamics of **3** and **3 m** regarding the *T*_2_→*T*_1_ energy transfer based on the femto‐picosecond emission up‐conversion technique (see si for detailed description). We then first probed the relaxation dynamics of the emission at 600 nm for **3** in the solid state, the origin of which, similar to that of **3 m** , should be from both the *S*_1_ state and the *T*_2_ state (^3^MLCT), but is obscured (Figure 5c) due to rapid *S*_1_→*T*_2_ ISC and fast *T*_2_→*T*_1_ energy transfer. Note that the onset of the anthracene *T*_1_ emission is at ∼680 nm (cf. the steady‐state emission spectra of complex **3**). This makes it unlikely that the 600 nm emission is attributed to the *T*_1_ state of anthracene.

**Figure 9:**
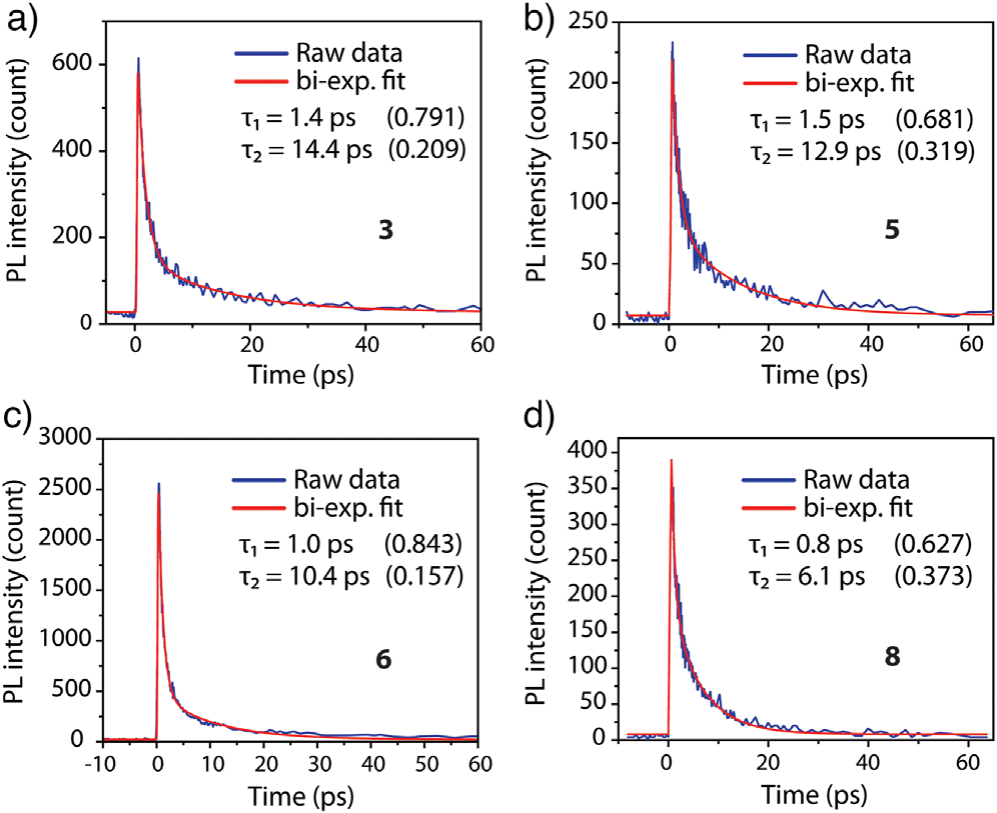
Early relaxation dynamics of crystalline complexes a) **3**, b) **5**, c) **6**, and d). **8** monitored at 600 nm emission (*λ*_exc_ = 410 nm). Relative amplitudes are shown in parentheses.

The results shown in Figure 9a and Table S12 clearly indicate that the early relaxation dynamics at 600 nm emission consist of two exponential decay components, which are fitted to be 1.4 and 14.4 ps. The undetectable rise lifetime monitored at 600 nm is due to the *S*_1_ state and the *T*_2_ state overlap, i.e., not from a single state in this region. The (1.4 ps)^−1^ decay can be attributed to the rate of *S*_1_→*T*_2_ ISC, which is ultrafast because of the heavy Pt atom‐enhanced SOC.^[^ ^57^, ^58^ ^]^ On the other hand, the (14.4 ps)^−1^ process can be assigned to the *T*_2_→*T*_1_ energy transfer. Such a fast energy transfer rate leads to ∼100% population at the *T*_1_ state for **3**, showing an anthracene‐like vibronically resolved phosphorescence maximized at 740 nm (Figure 5c). Note that here we cannot eliminate the possibility that the several picosecond timescales are in the same order as the rate of vibrational relaxation.

Nanosecond transient absorption (ns‐TA) spectroscopy applied to crystalline samples provided additional support for the *T*_2_→*T*_1_ energy transfer. The measurements in the sub‐µs timeframe reveal a prominent excited state absorption band around 420 nm for **3** (Figure S29), which is associated with the *T*_1_→*T*_n_ transition of the anthracene^[^ ^59^, ^60^ ^]^ to confirm the population of the *T*_1_ state. In comparison, the relaxation dynamics of **3 m** , monitored at 600 nm, gives a 3.9 ps fast *S*_1_→*T*_2_ decay component, accompanied by residual intensity that remains constant within 60 ps of the upconversion acquisition window (Figure S28).

The latter has been further resolved to give an average lifetime of 1.27 µs (Figure S26, vide supra). The results infer a much slower rate of *T*_2_→*T*_1_ energy transfer in **3 m**. Consequently, the *T*_2_→*S*_0_ phosphorescence for **3 m** becomes dominant, accompanied by a minor *T*_1_ phosphorescence (Figure 5c). The discrepancy between **3** and **3 m** can be rationalized by the structural variation, i.e., *ortho*‐ (**3**) versus *meta*‐ (**3 m**) configuration of phosphane ligands. In this view, the *T*_2_→*T*_1_ energy transfer rate for **3 m** is significantly reduced due to a longer separation between Pt(phbipy) and anthracene moieties of ∼5.3 Å (cf. ∼3.2 Å for **3**, vide supra) as well as their mutual orientation that is unfavorable for energy transfer. Further evidence of fast *T*_2_→*T*_1_ energy transfer is supported by **4** and **5**, for which the Pt(II) complex and anthracene are in the same parallel orientation as that of **3**. The early relaxation dynamics for **4** having terpy ligand, and for **5** with anthracene embedded between two Pt(phbipy) moieties exhibit decay times of *T*_2_ of 16.2 and 12.9 ps, respectively (Figure 9 and Figure S28); the fast *T*_2_→*T*_1_ energy transfer rationalizes the exclusive anthracene *T*_1_ phosphorescence observed for crystalline **3**–**5** (Figure 5c).

**Figure 10:**
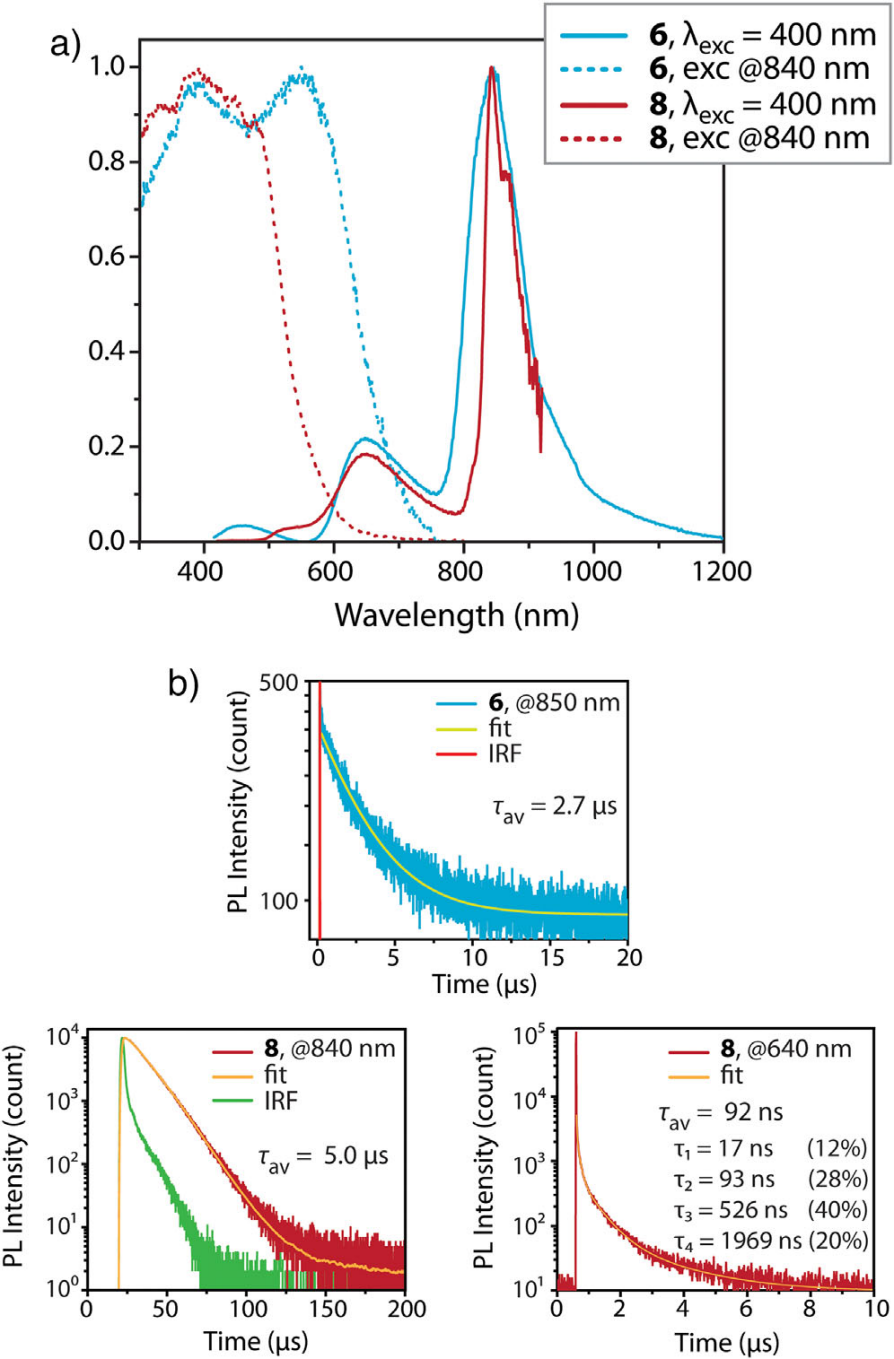
a) Normalized emission spectra for crystalline complexes **6** and **8**. b) Depicts the time‐resolved emission decays at 298 K with nano‐microsecond resolution based on time‐correlated single photon counting (TCSPC) for **6** and multichannel scaling (MCS) for **8**.

We then investigated two other groups of complexes with an aim of achieving lower energy photoluminescence. Compounds **6** and **7** were designed by tailoring ethynyl‐N,N‐dimethylaniline pendant to the energy acceptor anthracene, whereas **3e**, **8**, and **9** were derived by incorporating ethynyl spacer between the *ortho‐*substituted phenylene and the anthracene motif (Scheme 1). The crystalline forms of **6** and **7** exhibit weak NIR emission bands peaking at ca. 840 nm and ca. 890 nm, respectively (Figure 10a and Figure S25). The ethynyl‐anthracene complex **3e** weakly emits with the maximum at 753 nm (Table 1), while the luminescence of diethynyl‐anthracene derivative **8** is significantly more intense showing the main band at 843 nm under ambient conditions and 835 nm at 77 K (Figure 10 and Figure S25). Compound **9** is virtually nonemissive at room temperature but exhibits a structured main band peaking at 829 nm at 77 K. The photoluminescence quantum yield is below the current detection limit using an integrating sphere, which is estimated to be less than 2%. Upon monitoring at 840–880 nm, the lifetimes of the decays were fitted to be 2.7 µs for **6** and 5.0 µs for **8** (Figure 10b, Table 1), ensuring their phosphorescent nature. The luminescence of **7** was too weak for adequate determination of the lifetime. Noticeably, the phosphorescence of **6** and **8** is accompanied by a minor ∼640 nm emission band, while **7** reveals a minor band at 588 nm. Monitoring the lifetime of **8** at 640 nm reveals a multiexponentially‐fitted decay with main relative contributions of 17, 93, 526, and 1969 ns, Figure 10b), which likely suggests its phosphorescent origin. Residual intraphosphane fluorescence though cannot be excluded judged by the large *ε* value measured at the *S*_0_→*S*_1_ peak wavelength and pointing to an allowed character of *S*_1_ state for **6**–**9**. The exact origin of these signals, nevertheless, is ambiguous and could arise from packing effects.

**Scheme 2:**
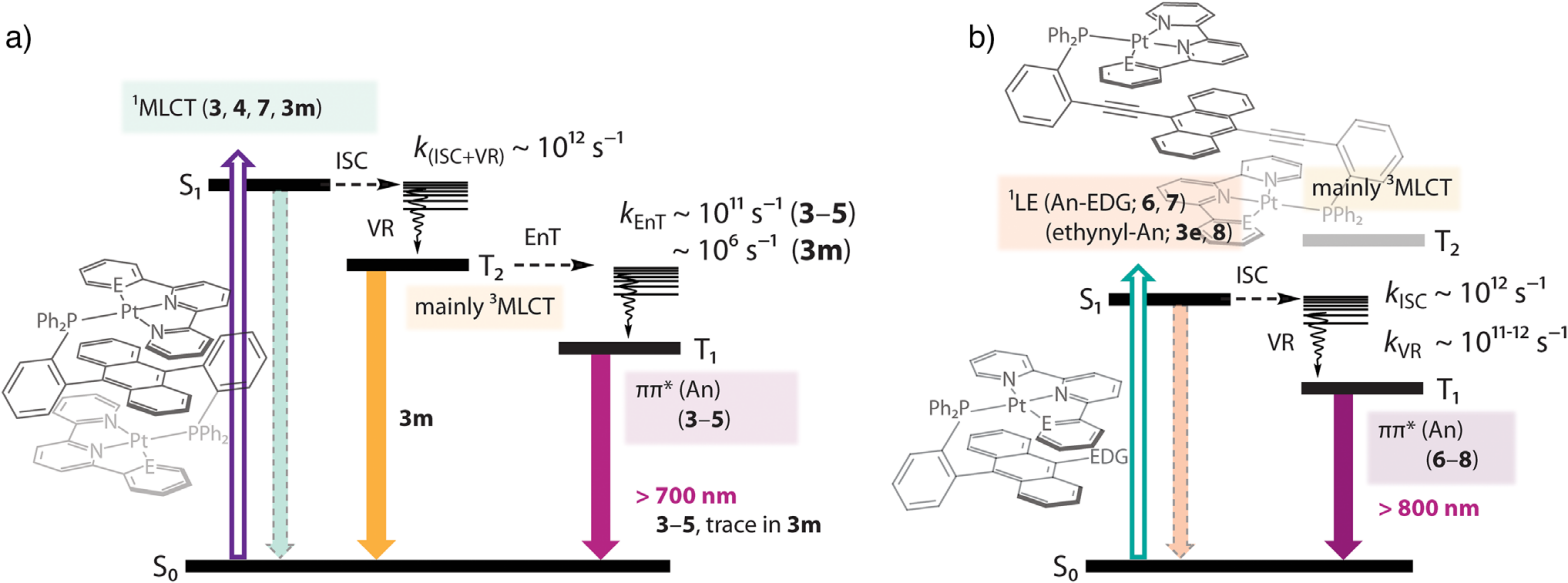
a) Relaxation pathways of the composite molecules **3**–**5**, and **3 m** and b) **6**–**8** and **3e** presented in this study. ISC: intersystem crossing, VR: vibrational relaxation, E_n_T: energy transfer.

As shown above, the lowest‐lying singlet excited singlet state (*S*_1_) for both groups **6**/**7** and **3e**/**8**/**9** has a large intraligand character associated with the 4‐(anthracen‐9‐ylethynyl)dimetylamino and (di)ethynyl‐anthracene moieties (Figures 7 and 8 and Figure S21), with the trend in energy levels *T*_2_ > *S*_1_ > *T*_1_ different from that for **3**–**5** (see Table S10 and Scheme 2). This results in a fast *S*_1_→*T*_1_ ISC (1.0, 1.4, and 0.8 ps for **6**, **7**, **8**, respectively) followed by vibrational relaxation (10.4, 15.6, and 6.1 ps for **6**, **7**, and **8**, respectively), Figure 9 and Figure S28. Despite the rapid relaxation dynamics, the ISC leading to the population of the PAH‐centred triplet states should be highly sensitive to the mutual disposition of the platinum complex entity and the anthracene π‐system of the phosphine ligand. A loss of crystallization solvent causing phase inhomogeneity and change of intramolecular conformations might decrease the interaction between spatially separated components. This can account for the observed dual emission in **6**–**8**.

It should be emphasized that phosphorescence of 9,10‐bis(phenylethynyl)anthracene (BPEA) dye, which is the chromophore in complex **8**, has not been previously reported due to the low rate of ISC attributed to the large energy difference Δ*E*(*S_1_–T*_1_).^[^ ^61^ ^]^ The triplet state of BPEA can be produced by using a Pd(II) porphyrin sensitizer or in metal‐organic frameworks,^[^ ^62^, ^63^ ^]^ but no radiative relaxation was detected. BPEA and related molecules often demonstrate very intense green fluorescence, which is also observed for BPEA‐derived ***P* ^2e^_ox_** (*Φ*_em_ = 0.95, *λ*_max_ = 492 nm, Table S8 and Figure S24). However, in **3e**/**8**/**9**, the predicted fluorescence of the *S*_1_ state is drastically red‐shifted (*λ*_max_ = 698 (**3e**) and 732 nm (**8**), Table S11), likely due to the interaction of the electronically depleted Pt center with the ─C≡C─ moiety (see the optimized geometries and Figure S30). This structural relaxation decreases the Δ*E*(*S*_1_ *–T*_1_) energy gap (Table S10) that is anticipated to facilitate the ISC. The formation of the ethynyl–anthracene *T*_1_ state is evidenced by the ns‐TA spectrum (Figure S29) showing the bands at around 500 nm, which are compatible with the *T*_1_ absorption spectrum of the BPEA (445−495 nm).^[^ ^62^ ^]^ Although the luminescence intensity is weak, it is comparable with the weak NIR (>800 nm) phosphorescence for a number of mentioned organic compounds (*Φ*_em_ ≪10^−2^, vide supra).

Summarizing the above results and discussion, the overall excited‐state relaxation dynamics of **3**–**8**, **3 m** , and **3e** are generalized in Scheme 2. Thus, complexes **3**–**5** undergo fast *S*_1_→*T*_2_ ISC (few picoseconds) and subsequent *T*_2_→*T*_1_ intramolecular energy transfer in a time scale of 10–20 ps, giving solely the anthracene‐like phosphorescence that is enhanced by SOC via the intermolecular heavy Pt atom effect. On the other hand, due to the unfavorable geometry in the distance and orientation between the associated chromophores, complex **3 m** with *meta*‐configured phosphane undergoes rather inefficient energy transfer in the crystalline solid. This is evidenced by the long decay time of *T*_2_ of 1.27 µs, resulting in the dominant *T*_2_‐related ^3^MLCT emission, accompanied by a trace of the *T*_1_‐based anthracene phosphorescence. **6**/**7** and **3e**/**8** undergo a similar relaxation pattern as **3**–**5**, associated with the PAH chromophore. The disparity lies in the energy levels, which are in the order of *T*_2_(^3^MLCT) > *S_1_* > *T*_1_(^3^LE) for **6**/**7** and **3e**/**8**. Therefore, the excited‐state relaxation involves fast *S*_1_→*T*_1_ ISC, followed by vibrational relaxation. Nevertheless, the possibility of triplet–triplet energy transfer cannot be eliminated as electronic transitions within the platinum fragment should occur at high energy excitation (*λ*_exc_ < 400 nm). The importance of platinum center in ethynyl–anthracene complexes **3e** and **8**, in part, lies in the significant stabilization of ligand‐centered *S*_1_ state that drastically accelerates ISC and even allows for radiative relaxation of the low‐lying *T*_1_ state. However, the possible deviation in intramolecular arrangement and metal fragment–PAH interaction results in dual emission (**6**–**8**).

## Conclusion

In this study, we have designed a series of transition metal complexes with *ortho*‐ and *meta‐*phenylene substituted phosphanes containing anthracene chromophores. The ligand configuration in these molecular composites provides short spatial separation between sterically unhindered metal fragments and the polyaromatic motif, allowing for effective through‐space triplet energy transfer. In particular, the metal center can have a dramatic impact on the optical gap, ISC, and the radiative decay of the anthracene‐localized triplet state. The silver(I)‐pyridine and gold(I) iodide units in complexes **1** and **2** have little contribution to the ^1^IL(anthracene‐centered) states, indicating that the heavy atom effect alone is insufficient to attain phosphorescence. On the contrary, the bichromophoric platinum(II) pincer complexes **3**–**5** and **3 m** exhibit through‐space energy transfer, resulting in NIR ^3^IL(anthracene)‐based luminescence. The efficiency of sensitization and the rate of radiative *T*_1_→*S*_0_ decay depend significantly on the intramolecular distance between the metal and organic moieties. This is illustrated by the *meta*‐phenyl substituted phosphane complex **3 m** , which exhibits much less efficient energy transfer and predominantly {Pt(phbipy)}‐based phosphorescence in the solid state. The donor‐functionalized phosphane in complexes **6** and **7** exhibits much weaker π–π* state‐based phosphorescence of the anthracen‐9‐yl‐ethynyl pendant, although shifted to longer wavelengths. Importantly, even for a dye with particularly inefficient ISC, the utilized molecular design allowed to realize room temperature phosphorescence, which is observed in **8** for the first time for BPEA derivatives.

In these composite molecules, we foresee that a more robust covalent connection instead of phosphane, which will preserve a short distance between the sensitizer and polyaromatic fluorophore, can suppress photodegradation and open a way to an extended range of low‐energy absorbers and NIR triplet emitters. The interplay of structure, transition metal, and intramolecular interactions, demonstrated in this work, paves the way for the targeted design of materials with tailored optical properties.

## Supporting Information

The authors have cited additional references within the Supporting Information.^[^ ^64^, ^65^, ^66^, ^67^, ^68^, ^69^, ^70^, ^71^, ^72^, ^73^, ^74^, ^75^, ^76^, ^77^, ^78^, ^79^, ^80^ ^]^

## Conflict of Interests

The authors declare no conflict of interest.

## Supporting information



Supporting Information

Supporting Information

## Data Availability

The data are available from Cambridge Crystallographic Data Centre (https://www.ccdc.cam.ac.uk/structures/), in the Supporting Information, and from the authors on request.
