# Supplementary material for: Organic Room‐Temperature near‐IR Phosphorescence Harvested by Intramolecular Through‐Space Sensitization in Composite Molecules
Source: Angew Chem Int Ed Engl. 2025 Mar 23;64(21):e202503327. doi: 10.1002/anie.202503327 (PMC12176076; doi:10.1002/anie.202503327)
Supplement: Supplementary file 1 — Supporting Information [file ANIE-64-e202503327-s002.docx]

***Supporting Information***

Organic Room-Temperature Near-IR Phosphorescence Harvested by Intramolecular Through-Space Sensitization in Composite Molecules

Iida Partanen,^[a]^ Chao-Hsien Hsu,^[b]^ Emily Hsue-Chi Shi,^[b]^ Iván Maisuls,^[c]^ Toni Eskelinen,^[d]^ Antti J. Karttunen,^[d]^ Jarkko J. Saarinen,^[a]^ Cristian A. Strassert,^*[c]^ Andrey Belyaev,^[a]^ Pi-Tai Chou,^*[b]^ Igor O. Koshevoy^*[a]^

^[a]^ Department of Chemistry and Sustainable Technology, University of Eastern Finland, Yliopistokatu 7, 80100 Joensuu, Finland.

^[b]^ Department of Chemistry, National Taiwan University, Taipei 10617, Taiwan, Republic of China

^[c]^ Institut für Anorganische und Analytische Chemie, Universität Münster, CiMIC, SoN, CeNTech Heisenbergstraße 11, 48149 Münster, Germany.

^[d]^ Department of Chemistry and Materials Science, Aalto University, FI-00076 Aalto, Finland.

E-mail: [ca.s@uni-muenster.de](mailto:cstra_01@uni-muenster.de), [chop@ntu.edu.tw](mailto:chop@ntu.edu.tw), [igor.koshevoy@uef.fi](mailto:igor.koshevoy@uef.fi)

**Contents**

| ***Experimental section*** | S3–S10 |
| --- | --- |
| ***Table S1***. Crystal data and structure refinement for **1**–**6**, **3m**. | S14-S19 |
| ***Tables S2–S6.*** Selected bond length and angles for **1**–**9**, **3m**, **3e**. | S20, S22 |
| ***Figure S1***. Molecular structure of complex **4**. | S23 |
| ***Figure S2***. Molecular views of ligand ***P*^1m^** and two modifications of complex **3m** | S23 |
| ***Figure S3***. Molecular structure of complex **7**. | S24 |
| ***Figure S4***. Molecular structures of phosphane oxide ***P*^1e^_ox_** and complex **3e**. | S24 |
| ***Figure S5***. Molecular structure of complex **9**. | S25 |
| ***Figures S6****,* ***S7***. Crystallographically determined and DFT-optimized ground state geometries of complexes **3**–**9** and **3e**. | S26, S27 |
| ***Figures S8–S18***. ^31^P{^1^H} and ^1^H NMR spectra of complexes **1**–**9**, **3m**, **3e**. | S28–S33 |
| ***Figure S19***. Electron density difference plots for complexes **1** and **2**. | S33 |
| ***Figure S20***. Electron density difference plots for complexes **4** and **5**. | S34 |
| ***Figure S21***. Electron density difference plots for complexes **3e** and **9**. | S35 |
| ***Figure S22*.** Stability test of complex **3**. | S35 |
| ***Figure S23***. Emission and excitation spectra of complexes **1** and **2** at 77 K. | S36 |
| ***Figure S24***. Excitation, emission, and absorption spectra of phosphane oxides ***P*^1^_ox_**, ***P*^1da^_ox_**, ***P*^1e^_ox_**, and ***P*^2e^_ox_**. |  |
| ***Figure S25***. Emission and excitation spectra of complexes **3**–**9**, **3m** and **3e** at 77 K. | S37 |
| ***Table S7***. Photophysical properties of complexes **1**–**9**, **3m**, and **3e** in the solid state at 77 K. | S38 |
| ***Table S8***. Photophysical properties of phosphane oxides in the solid state and solution at 298 K. | S38 |
| ***Table S9***. TD-DFT-calculated lowest-lying singlet-singlet vertical excitations for **1**–**9**, **3m**, **3e** in CH_2_Cl_2_. | S39 |
| ***Table S10***. DFT-calculated energy differences between S_1_, T_2_, and T_1_ states for **1**–**9**, **3m**, **3e** at S_1_ geometry. | S40 |
| ***Table S11***. DFT-predicted singlet (S_1_→S_0_) and triplet (T_1_→S_0_) luminescence, and spin-orbit coupling matrix elements for **1**–**9**, **3m**, **3e**. | S41 |
| ***Figures S26****,* ***S27***. Lifetime data obtained by MCS for crystalline **3m**. | S41, S42 |
| ***Figure S28***. Early relaxation dynamics of crystalline complexes **3m**, **4**, **7**, **3e**. | S42 |
| ***Table S12***. Pertinent lifetime data for crystalline complexes **3**–**8**, **3m**, and **3e** determined using the fluorescence up-conversion technique. | S43 |
| ***Figure S29***. Nanosecond-transient absorption data for crystalline complexes **3**–**9**, **3m** and **3e**. | S44 |
| ***Figure S30***. Overlay of optimized geometries of S_0_ and S_1_ states for complexes **3e** and **8** showing the excited state Pt^…^C_2_ interaction. | S45 |
| ***References*** | S46 |

**Experimental section**

**General comments**

9-(2-Bromophenyl)anthracene,^[1]^ 9-(3-bromophenyl)anthracene,^[2]^ 9-ethynylanthracene,^[3]^ 9,10-diethynylanthracene,^[4]^ (2-(anthracen-9-yl)phenyl)diphenylphosphane (***P*^1^**),^[1]^ 9,10-bis(2-(diphenylphosphaneyl)phenyl)anthracene (***P*^2^**),^[5]^ 4-[(9-(2-diphenylphosphanylphenyl)anthracen-10-yl)ethynyl]-N,N-dimethylaniline (***P*^5^**),^[1]^ [Pt(phbipy)Cl] (Hphbipy = 6-phenyl-2,2'-bipyridine),^[6]^ [Pt(terpy)(acetonitrile)](CF_3_SO_3_)_2_ ^[7]^ were synthesized according to published procedures. Tetrahydrofuran (THF), toluene and diethyl ether were distilled over Na-benzophenone ketyl under a nitrogen atmosphere prior to use. Other reagents and solvents were used as received. ^1^H, ^31^P{^1^H}, ^13^C{^1^H} and ^1^H–^1^H COSY NMR spectra in solution were recorded on a JEOL 500 MHz spectrometer. Mass spectra were measured on a Bruker maXis II ESI-QTOF instrument in the ESI^+^ mode. Microanalyses were carried out at the analytical laboratory of the University of Eastern Finland.

**Syntheses**

**(3-(Anthracen-9-yl)phenyl)diphenylphosphane (*P*^1m^)**. The synthesis was carried out under a nitrogen atmosphere. 9-(3-Bromophenyl)anthracene (1.7 g, 5.1 mmol) was dissolved in THF (50 cm^3^), then the solution was cooled to –70 ^o^C and a solution of *n*-BuLi (1.6 M in hexanes, 3.3 cm^3^, 5.3 mmol) was added dropwise within 10 min. to give a deep yellow-orange solution. It was stirred for 45 min. below –50 ^o^C and then neat PPh_2_Cl (1.2 g, 5.4 mmol) was added dropwise at –70 ^o^C. The reaction mixture was allowed to reach room temperature and was stirred overnight. Then it was quenched with methanol (5 cm^3^) and evaporated. The resulting yellow oily material was washed with methanol (3×15 cm^3^) to give a pale yellow solid. It was purified by column chromatography (Silica gel 70–230 mesh, ⌀2.5×10 cm column, eluent hexane:CH_2_Cl_2_ 5:1→2.5:1 v/v mixture). The collected solution was concentrated to ca. 15 cm^3^ to afford a yellow-greenish microcrystalline material. The suspension was cooled with ice, solvents were filtered off, the solid was additionally washed with cold hexanes (15 cm^3^) and dried (2.0 g, 89%). ^31^P{^1^H} NMR (202 MHz, CDCl_3_; 298 K; δ): –4.5 (s, PPh_2_). ^1^H NMR (500 MHz, CDCl_3_, 298 K; *δ*): 8.48 (s, 1H), 8.04 d, *J*_HH_ 8.4 Hz, 2H), 7.66 (dd, *J*_HH_ 8.8 and 0.8 Hz, 2H), 7.57 (td, *J*_HH_ 7.8 and 1.4 Hz, 1H), 7.50–7.42 (m, 9H), 7.39–7.33 (m, 8H). ^13^C{^1^H} (126 MHz, CDCl_3_, 298 K; *δ*): 139.0 (d, *J* 7.2 Hz), 137.4 (m br), 136.8 (m br), 136.6, 136.5, 136.4, 133.9 (d, *J* 19.4 Hz), 132.8 (d, *J* 18 Hz), 132.0, 131.4, 130.2, 129.0, 128.7, 128.6, 128.5, 126.8, 126.7, 125.5, 125.2. Anal. Calcd for C_32_H_23_P: C, 87.65; H, 5.29. Found: C, 87.32; H, 5.37.

**9-((2-bromophenyl)ethynyl)anthracene**. The synthesis was carried out under a nitrogen atmosphere. 9-Ethynylanthracene (1.0 g, 5.0 mmol), 1-bromo-2-iodobenzene (2.0 g, 7.1 mmol) were dissolved in a mixture of degassed tetrahydrofuran (30 cm^3^) and diisopropylamine (30 cm^3^), then Pd(PPh_3_)_2_Cl_2_ (150 mg, 0.21 mmol), PPh_3_ (112 mg, 0.43 mmol) and CuI (40 mg, 0.21 mmol) were added in a flow of nitrogen. The reaction mixture was stirred overnight at 50 ^o^C. Black suspension was cooled to room temperature, filtered through a Celite pad and solvents were evaporated to leave black residue. Dichloromethane (100 cm^3^) and water (100 cm^3^) were added, the organic layer was further washed with water (3 × 50 cm^3^) and dried over anhydrous sodium sulphate. The solvents were evaporated, and solid was purified by column chromatography (ø 4 × 30 cm, Silica gel 70–230 mesh, eluent hexane:CH_2_Cl_2_ 1:0→10:1 v/v mixture) to give 9-((2-bromophenyl)ethynyl)anthracene as yellow-greenish crystalline material (1.05 g, 59%).^1^H NMR (500 MHz, CDCl_3_, 298 K; *δ*): 8.78 (d, *J*_HH_ 8.6 Hz, –C_14_H_9_, 2H), 8.45 (s, –C_14_H_9_, 1H), 8.02 (d, *J*_HH_ 8.6 Hz, –C_14_H_9_, 2H), 7.82 (dd, *J*_HH_ 7.5, 1.7 Hz, –C_6_H_4_–, 1H), 7.71 (d, *J*_HH_ 8.1 Hz, 1H), 7.58–7.67 (m, –C_14_H_9_, 2H), 7.47–7.55 (m, –C_14_H_9_, 2H), 7.39 (d, *J*_HH_ 8.1 Hz, –C_6_H_4_–, 1H), 7.25 (m, –C_6_H_4_–, 1H).

**(2-(Anthracen-9-ylethynyl)phenyl)diphenylphosphane (*P*^1e^)**. 9-((2-bromophenyl)ethynyl)anthracene (0.92 g, 2.57 mmol) was dissolved in tetrahydrofuran (40 ml), cooled to –78 ^o^C and *n*-BuLi (1.6 M in hexanes, 1.8 cm^3^, 2.82 mmol) was added dropwise within 5 min. The reaction mixture was stirred for 1 h at a temperature below –60 ^o^C. Then neat diphenylchlorophosphane (0.62 g, 2.82 mmol) was added dropwise and clear pale solution was allowed to reach room temperature. After stirring for 1 h at room temperature, methanol (5 cm^3^) was added, and volatiles were evaporated. The yellow solid was washed with methanol (3 × 10 cm^3^), dissolved in dichloromethane (ca. 10 cm^3^) and passed through a pad of Silica gel (70–230 mesh, ø4 × 5 cm). Removal of the solvent afforded yellow microcrystalline material (0.80 g, 67%). ^31^P{^1^H} NMR (202 MHz, CDCl_3_; 298 K; δ): –8.8 (s, PPh_2_). ^1^H NMR (500 MHz, CDCl_3_, 298 K; *δ*): 8.44 (m, –C_14_H_9_, 2H), 8.37 (s, –C_14_H_9_, 1H), 7.88 (dd, *J*_HH_ 7.4, 3.7 Hz, 1H), 7.31–7.52 (m, –C_14_H_9_ + Ph + –C_6_H_4_–, 15H), 7.29 (t, *J*_HH_ 7.6 Hz, 1H), 6.92 (dd, *J*_HH_ 7.9, 3.7 Hz, 1H). ^13^C{^1^H} (126 MHz, CDCl_3_, 298 K; *δ*): 140.0 (d, *J* 12.4 Hz), 136.7 (d, *J* 11.1 Hz), 134.2 (d, *J* 19.7 Hz), 132.8–133.1 (m), 131.1 (s), 128.4–129.0 (m), 127.9 (s), 127.3 (s), 126.6 (s), 125.7 (s), 117.3 (s), 99.8 (d, *J* 6.6 Hz), 92.6 (s). Anal. Calcd for C_34_H_23_P: C, 88.29; H, 5.01. Found: C, 88.08; H, 5.14.

**9,10-bis((2-bromophenyl)ethynyl)anthracene**. The synthesis was carried out under a nitrogen atmosphere. 9,10-Diethynylanthracene (1.2 g, 5.3 mmol), 1-bromo-2-iodobenzene (3.8 g, 13.4 mmol) were dissolved in a mixture of tetrahydrofuran (60 cm^3^) and diisopropylamine (30 cm^3^), then Pd(PPh_3_)_2_Cl_2_ (200 mg, 0.29 mmol), PPh_3_ (150 mg, 0.57 mmol) and CuI (60 mg, 0.31 mmol) were added in a flow of nitrogen. The reaction mixture was stirred overnight at 50 ^o^C. Red-brown suspension was cooled to room temperature, and water (50 ml) was added, followed by chloroform (100 cm^3^). The organic layer was washed with water (3 × 50 cm^3^), dried over anhydrous sodium sulphate, and passed through a pad of Silica gel (70–230 mesh, ø 3 × 10 cm). The solvents were evaporated, red-brown microcrystalline solid was washed with dichloromethane (2 × 5 cm^3^), methanol (2 × 5 cm^3^) and dried to give 9,10-bis((2-bromophenyl)ethynyl)anthracene of sufficient purity (1.9 g, 67 %). ^1^H NMR (500 MHz, CDCl_3_, 298 K; *δ*): 8.83 (m, –C_14_H_8_–, 4H), 7.84 (dd, *J*_HH_ 7.7, 1.7 Hz, –C_6_H_4_–, 2H), 7.72 (dd, *J*_HH_ 8.1, 1.2 Hz, –C_6_H_4_–, 2H), 7.66 (m, –C_14_H_8_–, 4H), 7.40 (td, *J*_HH_ 7.7, 1.2 Hz, –C_6_H_4_–, 2H), 7.72 (td, *J*_HH_ 8.1, 1.7 Hz, –C_6_H_4_–, 2H).

**9,10-Bis((2-(diphenylphosphanyl)phenyl)ethynyl)anthracene (*P*^2e^)**. The synthesis was carried out under a nitrogen atmosphere. 9,10-Bis((2-bromophenyl)ethynyl)anthracene (0.9 g, 1.68 mmol) was dissolved in tetrahydrofuran (100 cm^3^), cooled to –78 ^o^C and treated dropwise with *n*-BuLi (1.6 M in hexanes, 2.2 cm^3^, 3.52 mmol). A brick-brown suspension was formed, which was stirred for 1.5 at a temperature below –50 ^o^C. Then neat diphenylchlorophosphane (0.8 g, 3.62 mmol) was added dropwise and resulting bright yellow suspension was allowed to reach room temperature. After stirring overnight, volatiles were evaporated. The yellow solid was washed with methanol (3 × 10 cm^3^), dissolved in chloroform (ca. 150 cm^3^) and passed through a pad of Silica gel (70–230 mesh, ø3 × 10 cm). Removal of the solvent afforded orange microcrystalline solid, which was washed with a dichloromethane-methanol 1:1 v/v mixture (2 × 10 cm^3^) and dried (0.95 g, 76%). ^31^P{^1^H} NMR (202 MHz, CD_2_Cl_2_; 298 K; δ): –9.0 (s, PPh_2_). ^1^H NMR (500 MHz, CD_2_Cl_2_, 298 K; *δ*): 8.44 (m, –C_14_H_8_–, 4H), 7.82–7.92 (m, –C_6_H_4_–, 2H), 7.51 (m, –C_14_H_8_–, 4H), 7.41–7.48 (m, –C_6_H_4_–, 2H), 7.27–7.38 (m, Ph + –C_6_H_4_–, 22H), 6.85–6.93 (m, –C_6_H_4_–, 2H). ^13^C{^1^H} (126 MHz, CD_2_Cl_2_, 298 K; *δ*): 140.2 (d, *J* 12.7 Hz), 136.5 (d, *J* 11.1 Hz), 134.1 (d, *J* 19.9 Hz), 133.2 (s), 133.1 (s), 132.1 (s), 128.2–128.9 (m), 127.4 (s), 126.9 (s), 118.4 (s), 101.6 (s), 92.3 (s). Anal. Calcd for C_54_H_36_P_2_: C, 86.85; H, 4.86. Found: C, 86.43; H, 5.02.

**[Ag(*P*^1^)(pyridine)_2_][ClO_4_] (1)**. AgClO_4_ (19 mg, 0.15 mmol) and ***P*^1^** (40 mg, 0.09 mmol) were mixed in dichloromethane (10 cm^3^) under a nitrogen atmosphere. A few drops of pyridine were added to the reaction mixture protected from light and stirring was continued for one hour. The yellowish solution was evaporated. The solid residue was recrystallized by gas-phase diffusion of diethyl ether into a dichloromethane/methanol solution of crude **1** in the presence of an excess of pyridine at + 4 °C affording nearly colorless crystalline material (61 mg, 86 %). ^31^P{^1^H} NMR (202 MHz, CD_2_Cl_2_, 298 K; *δ*): 6.8 (d, *J*_109AgP_ 752 Hz, *J*_107AgP_ 651 Hz). ^1^H NMR (500 MHz, CD_2_Cl_2_, 298 K; *δ*): 8.34 (s br, –C_14_H_9_, 1H), 8.11–8.13 (m, py, 4H), 7.77–7.82 (m, –C_14_H_9_ + py, 4H), 7.75 (t, *J*_HH_ 7.6 Hz, –C_6_H_4_–, 1H), 7.67 (t, *J*_HH_ 7.6 Hz, –C_6_H_4_–, 1H), 7.51 (t br, *J*_HH_ 8.5 Hz, –C_6_H_4_–, 1H), 7.18–7.45 (m, –C_14_H_9_ + py + –C_6_H_4_– + Ph, 21H). Anal. Calcd for C_42_H_33_AgClN_2_O_4_P: C, 62.74; H, 4.14; N, 3.48. Found: C, 62.33; H, 4.29; N, 3.14.

**AuI(*P*^1^) (2)**. Gold(I) iodide (75 mg, 0.23 mmol) and ***P*^1^** (101 mg, 0.23 mmol) were mixed in dichloromethane (10 cm^3^) under a nitrogen atmosphere and stirred for one hour. The orange solution was evaporated. The solid residue was recrystallized by a gas-phase diffusion of diethyl ether into a dichloromethane solution of crude **2** affording pale yellow crystalline material (92 mg, 53 %). ^31^P{^1^H} NMR (202 MHz, CDCl_3_, 298 K; *δ*): 31.9 (s). ^1^H NMR (500 MHz, CDCl_3_, 298 K; *δ*): 8.56 (s, –C_14_H_9_, 1H), 8.01 (d, *J*_HH_ 8.5 Hz, –C_14_H_9_, 2H), 7.70 (tm, *J*_HH_ 7.6 Hz, –C_6_H_4_–, 1H), 7.59 (tm, *J*_HH_ 7.6 Hz, –C_6_H_4_–, 1H), 7.44 (m, –C_6_H_4_–, 2H), 7.25–7.40 (m, Ph + –C_14_H_9_, 12H), 7.02–7.07 (m, –C_14_H_9_, 4H). Anal. Calcd for C_32_H_23_AuIP: C, 50.41; H, 3.04. Found: C, 50.46; H, 3.24.

**[Pt(phbipy)(*P*^1^)][CF_3_SO_3_]** **(3)**. [Pt(phbipy)Cl] (68 mg, 0.15 mmol) was dissolved in a mixture of dichloromethane (25 cm^3^) and methanol (3 cm^3^) and a solution of AgCF_3_SO_3_ (41 mg, 0.16 mmol) in acetonitrile (4 cm^3^) was added. The resulting suspension was briefly heated to reflux and stirred at room temperature for 1 hour, then filtered to remove the precipitate of AgCl. The solution was degassed and ***P*^1^** (70 mg, 0.16 mmol) was added under a nitrogen atmosphere. The reaction mixture was stirred for 1 hour at room temperature and evaporated. The solid was recrystallized by a gas-phase diffusion of diethyl ether into a dichloromethane-acetonitrile (5:1 v/v) solution of crude **3**, affording yellow crystalline material (122 mg, 82 %). ^31^P{^1^H} NMR (202 MHz, acetonitrile-*d*_3_, 298 K; *δ*): 21.0 (d, *J*_PPt_ 4095 Hz). ^1^H NMR (500 MHz, acetonitrile-*d*_3_, 298 K; *δ*): 8.50 (m, 1H), 8.07 (t, *J*_HH_ 8.0 Hz, 1H), 7.98 (d, *J*_HH_ 7.5 Hz, 1H), 7.89-7.93 (m, 3H), 7.85 (d, *J*_HH_ 8.0 Hz, 1H), 7.77 (d, *J*_HH_ 8.5 Hz, 1H), 7.69 (s, 1H), 7.45–7.66 (m br, 5H), 7.37–7.42 (m, 3H), 7.31 (d, *J*_HH_ 8.4 Hz, 2H), 7.26 (t, *J*_HH_ 7.7 Hz, 4H), 7.20–7.40 (very br, 2H), 7.03 (td, *J*_HH_ 7.6 and 1.0 Hz, 1H), 6.92 (br, 2H), 6.58–6.85 (very br, 2H), 6.64 (td, *J*_HH_ 7.5, 1.0 Hz, 1H), 6.54 (dd, *J*_HPt_ 51 Hz, *J*_HH_ 7.8 Hz, 1H), 6.43 (m, 1H), 5.56 (d, *J*_HH_ 6.0 Hz, 1H). Anal. Calcd for C_49_H_34_F_3_N_2_O_3_PPtS: C, 58.05; H, 3.38; N, 2.76; S, 3.16. Found: C, 58.02; H, 3.45; N, 2.87; S, 3.08.

**[Pt(terpy)(*P*^1^)][CF_3_SO_3_]_2_ (4)**. [Pt(terpy)(acetonitrile)](CF_3_SO_3_)_2_ (132 mg, 0.17 mmol) was suspended in a mixture of dichloromethane (15 cm^3^) and acetonitrile (2 cm^3^), which was degassed. Then ***P*^1^** (78 mg, 0.18 mmol) was added under a nitrogen atmosphere. The resulting red solution was stirred at room temperature for 2 h and then evaporated to dryness. The red solid was recrystallized by a gas-phase diffusion of diethyl ether into an acetonitrile solution of crude **4**, affording red crystalline material (180 mg, 90 %). ^31^P{^1^H} NMR (202 MHz, acetonitrile-*d*_3_, 298 K; *δ*): 8.1 (d, *J*_PPt_ 3642 Hz, 1P). ^1^H NMR (500 MHz, acetonitrile-*d*_3_, 298 K; *δ*): 8.57 (ddd, *J*_HH_ 9.5, 8.0, 1.3 Hz, –C_6_H_4_–, 1H), 8.52 (t, *J*_HH_ 8.0 Hz, terpy, 1H), 8.25 (dd, *J*_HH_ 8.0, 1.5 Hz, terpy, 2H), 8.03–8.12 (m, –C_14_H_9_ + –C_6_H_4_–, terpy, 6H), 7.91 (s, 1H), 7.80 (ddd, *J*_HP_ 12.4 Hz, *J*_HH_ 8.1, 1.0 Hz, *ortho*-H Ph, 4H), 7.75 (ddd, *J*_HH_ 7.0, 5.3, 1.3 Hz, –C_6_H_4_–, 1H), 7.56 (tm, *J*_HH_ 7.4 Hz, *para*-H Ph, 2H), 7.43 (td, *J*_HH_ 7.5, 2.9 Hz, *meta*-H Ph, 4H), 7.34 (dd, *J*_HH_ 8.5, 4.0 Hz, –C_14_H_9_ + terpy, 4H), 6.95 (ddd, *J*_HH_ 8.5, 6.3, 0.9 Hz, –C_14_H_9_, 2H), 6.82–6.85 (m, –C_14_H_9_ + terpy, 4H), 6.59 (dd, *J*_HPt_ ca. 36, *J*_HH_ 5.9, terpy, 2H). Anal. Calcd for C_49_H_34_F_6_N_3_O_6_PPtS_2_: C, 50.52; H, 2.94; N, 3.61; S, 5.50. Found: C, 50.50; H, 3.06; N, 3.99; S, 5.18.

**[Pt(phbipy)(*P*^1m^)][CF_3_SO_3_]** **(3m)**. Prepared analogously to **3** from [Pt(phbipy)Cl] (140 mg, 0.30 mmol), AgCF_3_SO_3_ (78 mg, 0.30 mmol), and ***P*^1m^** (133 mg, 0.30 mmol). Recrystallization by a gas-phase diffusion of diethyl ether into a dichloromethane solution of crude **3m** afforded yellow crystalline material (236 mg, 77 %). ^31^P{^1^H} NMR (202 MHz, acetonitrile-*d*_3_, 298 K; *δ*): 25.3 (d, *J*_PPt_ 4048 Hz). ^1^H NMR (500 MHz, acetonitrile-*d*_3_, 298 K; *δ*): 8.46 (s, –C_14_H_9_, 1H), 7.93–8.06 (m, 11H), 7.82 (d, *J*_HH_ 8.0 Hz, bipy, 1H), 7.76 (d, *J*_HH_ 8.0 Hz, bipy, 1H), 7.75 (tdd, *J*_HH_ 7.7, 2.7 and 0.5 Hz, 1H), 7.59–7.64 (m, 3H), 7.54–7.50 (m, 4H), 7.45 (dd, *J*_HH_ 7.8 and 1.4 Hz, phbipy, 1H), 7.36–7.40 (m, 4H), 7.27 (dd, *J*_HH_ 6.4 and 1.2 Hz, 1H), 7.25 (dd, *J*_HH_ 6.4 and 1.3 Hz, 1H), 7.13 (ddd, *J*_HH_ 7.3, 5.6 and 1.7 Hz, bipy, 1H), 7.00 (td, *J*_HH_ 7. 6 and 1.1 Hz, phbipy, 1H), 6.81 (dm, *J*_HH_ 5.7 Hz, bipy, 1H), 6.73 (td, *J*_HH_ 7. 6 and 1.5 Hz, phbipy, 1H), 6.57 (ddm, *J*_HPt_ ca. 53 Hz, *J*_HH_ 7.9 and 2.3 Hz, 1H). Anal. Calcd for C_49_H_34_F_3_N_2_O_3_PPtS: C, 58.04; H, 3.38; N, 2.76; S, 3.16. Found: C, 57.77; H, 3.14; N, 2.35; S, 2.89.

**[Pt(phbipy)(*P*^1e^)][CF_3_SO_3_]** **(3e)**. Prepared analogously to **3** from [Pt(phbipy)Cl] (68 mg, 0.15 mmol), AgCF_3_SO_3_ (41 mg, 0.16 mmol), and ***P*^1e^** (72 mg, 0.15 mmol). Recrystallization by a gas-phase diffusion of diethyl ether into a dichloromethane-acetonitrile solution of crude **3e** afforded yellow crystalline material (98 mg, 63 %). ^31^P{^1^H} NMR (202 MHz, CD_2_Cl_2_, 298 K; *δ*): 21.2 (d, *J*_PPt_ 4112 Hz). ^1^H NMR (500 MHz, CD_2_Cl_2_, 298 K; *δ*): 8.24 (s, –C_14_H_9_, 1H), 8.05 (d, *J*_HH_ 8.5 Hz, –C_14_H_9_, 2H), 8.00 (ddd, *J*_HH_ 7.4, 4.0, 1.0 Hz, –C_6_H_4_–, 1H), 7.91 (dd, *J*_HH_ 9.7, 8.0 Hz, –C_6_H_4_–, 1H), 7.84 (d, *J*_HH_ 8.5 Hz, –C_14_H_9_, 2H), 7.76 (t, *J*_HH_ 8.0 Hz, –C_14_H_9_, 2H), 7.71 (tt, *J*_HH_ 7.6 and 1.2 Hz, –C_6_H_4_–, 1H), 7.67–7.86 (m br, 3H), 7.60–7-65 (m, *ortho*-H Ph, 4H), 7.48–7.55 (m, *meta*-H Ph + ph, 5H), 7.29 (ddd, *J*_HH_ 8.0, 6.4, 1.1 Hz, –C_14_H_9_, 2H), 7.03–7.14 (m, phbipy + –C_6_H_4_–, 5H), 7.01 (d, *J*_HH_ 6.9 Hz, phbipy, 1H), 6.71 (td, *J*_HH_ 7.7, 1.4 Hz, phbipy, 1H), 6.59 (ddd, *J*_HPt_ ca. 52 Hz, *J*_HH_ 7.3 and 2.2 Hz, ph, 1H), 6.17 (d, *J*_HH_ 5.4 Hz, bipy, 1H), 5.63 (ddd, *J*_HH_ 7.1, 5.6 and 1.4 Hz, phbipy, 1H). Anal. Calcd for C_51_H_34_F_3_N_2_O_3_PPtS: C, 59.02; H, 3.30; N, 2.70; S, 3.09. Found: C, 59.13; H, 3.50; N, 3.01; S, 2.98.

**[{Pt(phbipy)}_2_(*P*^2^)][CF_3_SO_3_]_2_** **(5)**. Prepared analogously to **3** from [Pt(phbipy)Cl] (100 mg, 0.22 mmol), AgCF_3_SO_3_ (56 mg, 0.22 mmol), and ***P*^2^** (76 mg, 0.11 mmol). Recrystallization by a gas-phase diffusion of diethyl ether into a dichloromethane-acetonitrile solution of crude **7** afforded yellow crystalline material (144 mg, 72 %). ^31^P{^1^H} NMR (202 MHz, acetonitrile-*d*_3_, 298 K; *δ*): 18.1 (d, *J*_PPt_ 4107 Hz). ^1^H NMR (500 MHz, CD_2_Cl_2_, 298 K; *δ*): 8.32 (d, *J*_HH_ 7.9 Hz, phbipy, 2H), 8.21–8.26 (m, phbipy, 2H), 8.12 (d, *J*_HH_ 8.0 Hz, 2H), 8.07 (t, *J*_HH_ 7.9 Hz, phbipy, 2H), 7.96 (td, *J*_HH_ 7.9, 1.4 Hz, phbipy, 2H), 7.73–7.82 (m, –C_14_H_8_–, 4H), 7.57 (d, *J*_HH_ 7.9 Hz, phbipy, 2H), 7.35–7.50 (br m, *ortho*-H Ph, 8H), 7.32 (t, *J*_HH_ 7.3 Hz, *para*-H Ph, 4H), 7.13–7.20 (m, *meta*-H Ph +–C_6_H_4_–, 10H), 6.88 (t, *J*_HH_ 7.3 Hz, –C_6_H_4_–, 2H), 6.65–6.80 (br s, phbipy, 4H), 6.54 (t, *J*_HH_ 6.3 Hz, –C_6_H_4_–, 2H), 6.39 (td, *J*_HH_ 7.6, 1.1 Hz, –C_6_H_4_–, 2H), 6.21–6.31 (br m, –C_14_H_8_–, 4H), 6.08 (q, *J*_HH_ 5.1 Hz, phbipy, 2H), 5.97 (ddd, *J*_HPt_ ca. 43 Hz, *J*_HH_ 7.7 and 1.9 Hz, phbipy, 2H), 5.80 (d, *J*_HH_ 5.1 Hz, phbipy, 2H). Anal. Calcd for C_84_H_58_F_6_N_4_O_6_P_2_Pt_2_S_2_: C, 54.55; H, 3.16; N, 3.03; S, 3.47. Found: C, 54.14; H, 3.32; N, 3.31; S, 3.60.

**[Pt(phbipy)(*P*^1da^)][CF_3_SO_3_]** **(6)**. Prepared analogously to **3** from [Pt(phbipy)Cl] (80 mg, 0.17 mmol), AgCF_3_SO_3_ (46 mg, 0.18 mmol), and ***P*^1da^** (101 mg, 0.17 mmol). Recrystallization by a gas-phase diffusion of diethyl ether into an acetonitrile solution of crude **6** afforded red crystalline material (148 mg, 74 %). ^31^P{^1^H} NMR (202 MHz, acetonitrile-*d*_3_, 298 K; *δ*): 20.3 (d, *J*_PPt_ 4089 Hz). ^1^H NMR (500 MHz, acetonitrile-*d*_3_, 298 K; *δ*): 8.51 (m, 1H), 8.00 (t, *J*_HH_ 8.0 Hz, 1H), 7.20–8.00(very br, 7H), 7.83–7.97 (m, 6H), 7.73 (d, *J*_HH_ 8.0 Hz, 2H), 7.64 (dd, *J*_HH_ 9.2 and 4.7 Hz, 1H), 7.51 (dm, *J*_HH_ 9.0 Hz, 2H), 7.36–7.42 (m, 3H), 7.28 (td, *J*_HH_ 7.6 and 1.9 Hz, 4H), 6.80–7.20(very br, 3H), 7.01 (td, *J*_HH_ 7.5, 0.8 Hz, 1H), 6.83 (dm, *J*_HH_ 9.0 Hz, 2H), 6.61 (td, *J*_HH_ 7.6, 1.4 Hz, 1H), 6.48–6.50 (m, 2H), 5.57 (d, *J*_HH_ 5.4 Hz, 1H), 3.03 (s, Me, 6H). Anal. Calcd for C_59_H_43_F_3_N_3_O_3_PPtS: C, 61.24; H, 3.63; N, 3.75; S, 2.77. Found: C, 60.94; H, 4.01; N, 3.80; S, 2.36.

**[Pt(terpy)(*P*^1da^)][CF_3_SO_3_]_2_ (7)**. Prepared analogously to **4** from [Pt(terpy)(acetonitrile)](CF_3_SO_3_)_2_ (92 mg, 0.12 mmol) and ***P*^1da^** (75 mg, 0.13 mmol) . Recrystallization by a gas-phase diffusion of diethyl ether into a dichloromethane solution of crude **7** afforded brown-black crystalline material (139 mg, 89 %). ^31^P{^1^H} NMR (202 MHz, acetonitrile-*d*_3_, 298 K; *δ*): 6.9 (d, *J*_PPt_ 3626 Hz). ^1^H NMR (500 MHz, acetonitrile-*d*_3_, 298 K; *δ*): 8.56 (ddd, *J*_HH_ 9.5, 8.0, 1.0 Hz, –C_6_H_4_–, 1H), 8.29 (dd, *J*_HH_ 8.4 and 7.8 Hz, terpy, 1H), 8.07–8.18 (m, –C_14_H_8_ + –C_6_H_4_– + terpy terpy, 6H), 8.01 (dd, *J*_HH_ 7.5 and 1.5 Hz, terpy, 2H), 7.92 (d, *J*_HH_ 8.5 Hz, –C_14_H_8_, 2H), 7.84–7.90 (m, –C_14_H_9_ + *ortho*-H Ph, 5H), 7.60 (tm, *J*_HH_ 7.5 Hz, *para*-H Ph, 2H), 7.52 (dm, *J*_HH_ 8.7 Hz, –C_6_H_4_–NMe_2_, 2H), 7.43–7.49 (m, *meta*-H Ph + –C_14_H_8_, 6H), 7.05 (ddd, *J*_HH_ 8.4, 6.8, 1.0 Hz, –C_14_H_8_, 2H), 6.95 (ddd, *J*_HH_ 8.5, 6.5, 1.0 Hz, –C_14_H_8_, 2H), 6.84–6.87 (m, terpy + –C_6_H_4_–NMe_2_, 4H), 6.52 (dd, *J*_HPt_ ca. 36 Hz, *J*_HH_ 5.9 Hz, terpy, 2H), 3.08 (s, Me,6H). Anal. Calcd for C_59_H_43_F_6_N_4_O_6_PPtS_2_: C, 54.17; H, 3.31; N, 4.28; S, 4.90. Found: C, 53.86; H, 3.32; N, 4.27; S, 4.41.

**[{Pt(phbipy)}_2_(*P*^2e^)][CF_3_SO_3_]_2_** **(8)**. Prepared analogously to **3** from [Pt(phbipy)Cl] (100 mg, 0.22 mmol), AgCF_3_SO_3_ (56 mg, 0.22 mmol), and ***P*^2e^** (83 mg, 0.11 mmol). Recrystallization by a gas-phase diffusion of diethyl ether into a dichloromethane-acetonitrile-methanol solution of crude **8** afforded yellow crystalline material (167 mg, 81 %). ^31^P{^1^H} NMR (202 MHz, acetonitrile-*d*_3_, 298 K; *δ*): 21.0 (d, *J*_PPt_ 4092 Hz). ^1^H NMR (500 MHz, acetonitrile-*d*_3_, 298 K; *δ*): 8.12 (ddd, *J*_HH_ 7.6, 4.0, 1.1 Hz, –C_6_H_4_–, 2H), 7.90–7.95 (m, –C_6_H_4_–, 2H), (m, H Ph + –C_6_H_4_– + phbipy + –C_14_H_8_–, 34H), 7.04–7.10 (m, phbipy, 4H), 7.00–7.02 (dt, *J*_HH_ 7.0, 1.3 Hz, phbipy, 2H), 6.93–6.96 (m, phbipy, 2H), (m, –C_14_H_8_–, 4H), 6.66 (td, *J*_HH_ 7.6, 1.5 Hz, phbipy, 2H), 6.52 (ddd, *J*_HPt_ ca. 48 Hz, *J*_HH_ 7.8, 2.4, 0.9 Hz, phbipy, 2H), 6.00 (d, *J*_HH_ 5.3 Hz, phbipy, 2H), 5.59 (ddd, *J*_HH_ 7.0, 5.6, 1.5 Hz, phbipy, 2H). Anal. Calcd for C_88_H_58_F_6_N_4_O_6_P_2_Pt_2_S_2_: C, 55.70; H, 3.08; N, 2.95; S, 3.38. Found: C, 55.42; H, 3.21; N, 2.84; S, 3.18.

**[{Pt(terpy)}_2_(*P*^2e^)][CF_3_SO_3_]_4_ (9)**. Prepared analogously to **4** from [Pt(terpy)(acetonitrile)](CF_3_SO_3_)_2_ (100 mg, 0.13 mmol) and ***P*^2e^** (49 mg, 0.07 mmol). Recrystallization by slow evaporation of an acetonitrile-toluene solution of crude **9** at +5 ^o^C afforded red crystalline material (121 mg, 84 %). ^31^P{^1^H} NMR (202 MHz, acetonitrile-*d*_3_, 298 K; *δ*): 12.4 (d, *J*_PPt_ 3656 Hz). ^1^H NMR (500 MHz, acetonitrile-*d*_3_, 298 K; *δ*): 8.28 (ddd, *J*_HH_ 7.7, 4.3, 1.1 Hz, –C_6_H_4_–, 2H), 8.06–8.11 (m, –C_6_H_4_–, 2H), 8.02 (t, *J*_HH_ 8.1 Hz, terpy, 2H), 7.92–7.96 (m, *ortho*-H Ph + –C_6_H_4_–, 10H), 7.76–7.82 (m, *meta*-H Ph + –C_6_H_4_– + –C_14_H_8_–, 14H), 7.63–7.71 (m, *para*-H Ph + terpy, 16H), 7.17–7.20 (m, terpy, 2H), 6.89–6.93 (m, –C_14_H_8_–, 4H), 6.61–6.63 (ddd, *J* 7.7, 5.9, 1.5 Hz , terpy, 4H). Anal. Calcd for C_88_H_58_F_12_N_6_O_12_P_2_Pt_2_S_4_: C, 48.05; H, 2.66; N, 3.82; S, 5.83. Found: C, 48.29; H, 2.84; N, 3.99; S, 5.67.

**General procedure for** ***P*^1^_ox_**, ***P*^1da^_ox_**, ***P*^1e^_ox_** and ***P*^2e^_ox_**. The corresponding phosphane (0.1 mmol) was dissolved in dichloromethane (15 cm^3^) and a three-fold excess of 30 % (w/w) aqueous H_2_O_2_ (ca. 0.3 mmol) was added. The reaction mixture was stirred for 30 min under ambient conditions and then was washed with water (3×10 cm^3^). The organic phase was separated, filtered through a pad of anhydrous sodium sulphate (⌀1×2 cm), and evaporated to dryness *in vacuo*. The solid residue was purified by column chromatography (Silica gel 70-230 mesh, ⌀2.5×15 cm, eluent dichloromethane/1% methanol), except for ***P*^2e^_ox_**, which was washed with methanol (5 × 8 cm^3^) and diethyl ether (3 × 8 cm^3^) to give pure materials.

***P*^1^_ox_**. White powder (83%). ^1^H NMR (500 MHz, CDCl_3_, 298 K; *δ*): 8.14 (s, 1H), (dd, *J*_HP_ 13.1 Hz, *J*_HH_ 7.8 Hz, 1H), 7.80 (d, *J*_HH_ 8.5 Hz, 2H), 7.70 (t, *J*_HH_ 7.8 Hz, 1H), 7.62 (t, *J*_HH_ 7.8 Hz, 1H), 7.40 (d, *J*_HH_ 8.5 Hz, 2H), 7.34 (d, *J*_HH_ 8.5 Hz, 2H), 7.25 (t, *J*_HH_ 8.5 Hz, 2H), 7.14 (dd, *J*_HH_ 12.3, 8.0 Hz, 4H), 7.09 (d, *J*_HH_ 7.0 Hz, 2H), 7.14 (td, *J*_HH_ 8.0, 2.9 Hz, 4H). ^31^P{^1^H} NMR (202 MHz, CDCl_3_, 298 K; *δ*): 27.3 (s). Anal. Calcd for C_32_H_23_OP: C, 84.56; H, 5.10. Found: C, 84.39; H, 5.04.

***P*^1da^_ox_**. Orange powder (80%). ^1^H NMR (300 MHz, CDCl_3_, 298 K; *δ*): 8.51 (d, *J*_HH_ 8.7 Hz, 2H), 8.08 (dd, *J*_HP_ 12.8, *J*_HH_ 7.9 Hz, 1H), 7.65–7.76 (m, 4H), 7.27–7.49 (m, 9H), 7.09–7.16 (m, 6H), 6.81–6.93 (m, 6H). ^31^P{^1^H} NMR (202 MHz, chloroform-*d*_1_, 298 K; *δ*): 27.4 (s). Anal. Calcd for C_42_H_32_NOP: C, 84.40; H, 5.40; N, 2.34. Found: C, 84.59; H, 5.55; N, 2.27.

***P*^1e^_ox_**. Yellow powder (90%). ^1^H NMR (500 MHz, CDCl_3_, 298 K; *δ*): 8.36 (s, 1H), 8.17–8.19 (m, 2H), 7.89–7.95 (m, 3H), 7.70–7.78 (m, 5H), 7.63 (t, *J* = 7.7 Hz, 1H), 7.37–7.49 (m, 7H), 7.25–7.31 (m, 4H). ^31^P{^1^H} NMR (202 MHz, CDCl_3_, 298 K; *δ*): 31.3 (s). Anal. Calcd for C_34_H_23_OP: C, 85.34; H, 4.84. Found: C, 84.20; H, 4.99.

***P*^2e^_ox_**. Orange powder (88%). ^1^H NMR (500 MHz, CDCl_3_, 298 K; *δ*): 8.19–8.21 (m, 4H), 7.88–7.91 (m, 2H), 7.72–7.77 (m, 8H), 7.62–7.68 (m, 4H), 7.35–7.50 (m, 10H), 7.25–7.32 (m, 8H). ^31^P{^1^H} NMR (202 MHz, CDCl_3_, 298 K; *δ*): 30.7 (s). Anal. Calcd for C_54_H_36_O_2_P_2_: C, 83.28; H, 4.66. Found: C, 83.05; H, 4.57.

**X-ray diffractometric structural analysis**. The crystals of ***P*^1m^**, ***P*^1e^_ox_**, **1**–**9**, **3m**, **3e** were immersed in cryo-oil, mounted in a Nylon loop, and measured at a temperature of 150 K except **2**, measured at 170 K. The X-ray diffraction data were collected with a Bruker Kappa Apex II, a Rigaku SuperNova and a Rigaku Synergy S diffractometers using Mo K*α* (*λ* = 0.71073 Å) and Cu K*α* (*λ* = 1.54184 Å) radiations. The APEX^[8]^ and CrysAlisPro^[9]^ program packages were used for cell refinements and data reductions. A numerical absorption correction (SADABS^[10]^ or CrysAlisPro^[9]^) was applied to all data. The structures were solved by direct methods using the SHELXT-2018^[11]^ program with the WinGX^[12]^ graphical user interface. Structural refinements were carried out using SHELXL-2018.^[11]^

The CF_3_SO_3_^–^ counterions were disordered between two (**3**, **3m**, **7**, **8**) and three (**6**) orientations and refined with occupancies of components 0.71/0.29 (**3**), 0.79/0.21 (**3m** tricl.), 0.64/0.36 (**3m** monocl.), 0.46/0.30/0.24 (**6**), 0.68/0.32 (**7**), and 0.82/0.18 (**8**). A series of geometry and displacement constraints and restraints were applied to these moieties.

The crystallization solvent molecules in **5**–**7** could not be resolved unambiguously. The contribution of the missing solvent to the calculated structure factors was taken into account by using a *SQUEEZE* routine of *PLATON*.^[13]^ The missing solvent was not taken into account in the unit cell content.

All non-H atoms were anisotropically refined, and all hydrogen atoms were positioned geometrically and constrained to ride on their respective parent atoms with C−H = 0.95−0.98 A and *U*_iso_ = 1.2−1.5 *U*_equiv_ (parent atom). The crystallographic details are summarized in Table S1.

**Photophysical measurements**. The UV−vis absorption spectra in solution were measured on a Perkin Elmer Lambda 900 spectrophotometer using quartz 1 cm length cuvettes (Hellma®). Steady-state excitation and emission spectra were recorded on an Edinburgh Instruments FLS1000 fluorometer equipped with a 450 W ozone-free Xenon arc lamp (250–900 nm), a 10 W Xe flash-lamp (250–900 nm, pulse width *ca*. 1 µs) with repetition rates of 0.1 to 100 Hz, a picosecond diode laser (377 nm), double monochromators for the excitation and emission pathways, a photomultiplier (PMT-980) as a detector. The excitation and emission spectra were corrected using the standard corrections supplied by the manufacturer for the spectral power of the excitation source and the sensitivity of the detector. The emission was collected at right angles to the excitation source. The luminescence lifetimes were measured using a Xe flash-lamp in the multi-channel scaling (MCS) mode. The quality of the fit was assessed by minimizing the reduced chi squared function (χ^2^) and visual inspection of the weighted residuals and their autocorrelation. The solvents used were of spectrometric grade (Uvasol^®^). The solids were measured under an inert atmosphere of nitrogen using quartz EPR tubes. Prior to measurements, samples were dried under vacuum (10^-3^ mbar) for at least 30 minutes in order to avoid the influence of crystallization solvent.

**Step-scan pump-probe time-resolved UV–vis measurements (ns-TA).** Step-scan UV–vis spectroscopy was performed using a Vertex 80 spectrometer (Bruker) with a Si diode detector. The measurements were carried out exclusively in the crystalline state, with the crystals positioned on quartz substrates. The pump wavelength was set at the second harmonic (400 nm, pulse width 8 ns) of a Q-switched Nd: YAG pumped Ti: Sapphire laser, with the typical excitation energy adjusted to lower than 200 mJ cm^−2^. An optimized laser pulse had a signal-to-noise ratio adjusted to avoid damaging the sample. The background reduction and optimization of pumping power required various combinations of interference filters and were necessary to avoid thermal noise and sample decomposition. Moreover, the excitation source was synchronized with the spectrometer and was set at 45° with respect to the UV-vis probe beam to maximize the pump-probe overlap. In addition, the visible source (tungsten lamp, 24 V, 150 W) was employed as an external source coupled with a water-cooling unit and power supply.

**Stability test**. The observations imply that complexes **3**–**9** are prone to degradation when exposed to light and oxygen. To test their stability, we selected complex **3** for the demonstration. The sample preparation is as follows. A degassed cell with two entrances is used, the solvent is placed at one entrance, and the powder at the other. The entrance containing the solvent was degassed by three freeze-pump-thaw cycles to ensure that the oxygen is removed. After that, the powder is mixed with the degassed solvent. This procedure minimizes the possibility of sample oxidation during preparation of the solution. The results are shown in Figure S22.

**Fluorescence up-conversion.** A stable 120-femtosecond LASER oscillator performed this ultrafast fluorescence up-conversion measurement (FOG100-DX, CDP corp.). A pump beam of 410 nm was generated by the part of oscillator output traveling through the second harmonic generation (SHG, β-barium borate crystal). An iris selected the energy and the beam size of the pump after this SHG. The lens system was used to focus the pump beam on the sample, to collect the fluorescence, and to focus the fluorescence on sum-frequency BBO crystal (SHG, β-barium borate crystal), respectively. The studied crystalline compounds were uniformly distributed onto the cell. Subsequently, measurements were conducted using a rotating cell in a transmittance collection mode. The gate beam (820 nm) enters the delay line stage and crosses the fluorescence beam in the sum-frequency BBO with a collinear measurement. The polarized angle between the pump beam and the gate beam is set at the magic angle (54.7˚). A monochromator was applied and coupled with a PMT to record the sum-frequency signal. In this research, the FWHM of IRF is ~180 fs.

**Computational details**. Quantum chemical Density Functional Theory (DFT) and Time-Dependent Density Functional Theory (TD-DFT) using the Orca software (v5.0.3).^[14]^ Ground state geometries for all studied molecules were optimized with DFT, while TD-DFT was used for all excited state optimizations. Cationic models were used for the ionic species **1**, **3**–**9**, **3m**, **3e**, neglecting the counter-anions. An implicit C-PCM solvation model with dichloromethane as the solvent was used in all calculations.^[15]^ The long-range corrected hybrid density functional LRC-ωPBEh^[16]^ was used in all calculations together with a def2-TZVP basis set for all atoms and the corresponding effective core potential for metal atoms.^[17]^ To evaluate the spin-orbit coupling matrix elements between singlet and triplet states, single-point TD-DFT calculations with scalar-relativistic DKH Hamiltonian together with perturbative inclusion of spin-orbit coupling effects were performed.^[18]^ Relativistic calculations utilized recontracted variants of the def2 basis sets (DKH-def2-TZVP) as well as an all-electron SARC-DKH-TZVP basis for metal atoms. The resolution of identity as well as the chain of spheres for exchange approximation (RIJCOSX)^[19]^ was used in all calculations to reduce the computational burden.

***Table S1.*** Crystal data and structure refinement for **1**–**9**, **3m**, **3e**, ***P*^1m^** and ***P*^1e^_ox_**.

| **Identification code** | **1** | **2** | **3** | **3m (tricl.)** | **3m (monocl.)** |
| --- | --- | --- | --- | --- | --- |
| CCDC | 2370317 | 2370318 | 2370319 | 2370321 | 2370320 |
| Empirical formula | C_42_H_33_AgClN_2_O_4_P | C_32_H_23_AuIP | C_51_H_37_F_3_N_3_O_3_PPtS | C_50_H_36_Cl_2_F_3_N_2_O_3_PPtS | C_49_H_34_F_3_N_2_O_3_PPtS |
| Formula weight | 803.99 | 762.34 | 1054.95 | 1098.83 | 1013.90 |
| Temperature (K) | 150(2) | 170(2) | 150(2) | | |
| Wavelength (Å) | 0.71073 | | | | |
| Crystal system | Monoclinic | Monoclinic | Monoclinic | Triclinic | Monoclinic |
| Space group | *P*2_1_/*n* | *P*2_1_/*c* | *P*2_1_/*c* | $P\overline{1}$ | *C*2/*c* |
| Unit cell dimensions |  |  |  |  |  |
| a (Å) | 10.0575(10) | 15.4451(2) | 15.9328(6) | 10.4832(2) | 31.4298(10) |
| b (Å) | 14.3585(14) | 16.6309(2) | 18.4085(8) | 15.0021(4) | 9.1821(2) |
| c (Å) | 25.079(2) | 21.1922(3) | 15.2789(6) | 15.2504(4) | 33.7055(11) |
| a (^o^) | 90 | 90 | 90 | 67.419(2) | 90 |
| β (^o^) | 97.068(2) | 106.962(2) | 109.9950(10) | 85.724(2) | 109.712(4) |
| γ (^o^) | 90 | 90 | 90 | 79.603(2) | 90 |
| Volume (Å^3^) | 3594.2(6) | 5206.75(13) | 4211.2(3) | 2178.16(10) | 9157.1(5) |
| Z | 4 | 8 | 4 | 2 | 8 |
| ρ_calc_ (Mg/m^3^) | 1.486 | 1.945 | 1.664 | 1.675 | 1.471 |
| μ (mm^-1^) | 0.726 | 6.918 | 3.482 | 3.488 | 3.199 |
| F(000) | 1640 | 2896 | 2096 | 1088 | 4016 |
| Crystal size (mm^3^) | 0.533 × 0.238 × 0.195 | 0.201 × 0.066 × 0.029 | 0.567 × 0.151 × 0.115 | 0.340 × 0.140 × 0.080 | 0.310 x 0.150 x 0.100 |
| θ range for data collection (^o^) | 1.637 to 29.999 | 2.284 to 32.467 | 2.695 to 29.998 | 2.270 to 32.140 | 2.347 to 30.379 |
| Index ranges | -13≤h≤12,  -20≤k≤20,  -8≤l≤35 | -17≤h≤23,  -24≤k≤21,  -30≤l≤14 | -22≤h≤21,  -25≤k≤24,  -21≤l≤20 | -15≤h≤13,  -21≤k≤21,  -22≤l≤19 | -28≤h≤44,  -12≤k≤10,  -44≤l≤47 |
| Reflections collected | 15378 | 28370 | 44014 | 24626 | 21640 |
| Independent reflections | 10016 [R(int) = 0.0214] | 16873 [R(int) = 0.0309] | 12266 [R(int) = 0.0253] | 13748 [R(int) = 0.0257] | 12065 [R(int) = 0.0282] |
| Completeness to θ = 25.242^o^ | 98.0 % | 99.5 % | 99.8 % | 99.9 % | 99.9 % |
| Absorption correction | Numerical | Analytical | Numerical | | |
| Max. and min. transmission | 0.871 and 0.698 | 0.825 and 0.337 | 0.690 and 0.243 | 1.000 and 0.740 | 1.0000 and 0.793 |
| Refinement method | Full-matrix least-squares on F^2^ | | | | |
| Data/ restrains/ parameters | 10016 / 0 / 460 | 16873 / 0 / 631 | 12266 / 116 / 630 | 13748 / 118 / 629 | 12065 / 164 / 542 |
| GOOF on F^2^ | 1.014 | 1.049 | 1.013 | 1.043 | 1.347 |
| Final R indices [I>2σ(I)]^a^ | R_1_ = 0.0372, wR_2_ = 0.0823 | R_1_ = 0.0416, wR_2_ = 0.0745 | R_1_ = 0.0247, wR_2_ = 0.0495 | R_1_ = 0.0304, wR_2_ = 0.0645 | R_1_ = 0.0836, wR_2_ = 0.1403 |
| R indices (all data) | R_1_ = 0.0535, wR_2_ = 0.0897 | R_1_ = 0.0709, wR_2_ = 0.0852 | R_1_ = 0.0402, wR_2_ = 0.0545 | R_1_ = 0.0372, wR_2_ = 0.0678 | R_1_ = 0.0987, wR_2_ = 0.1454 |
| Largest diff. peak and hole (e.Å^-3^) | 1.134 and -0.418 | 1.407 and -1.247 | 1.308 and -1.023 | 1.406 and -1.326 | 2.077 and -2.656 |
| ^a^ R_1_ = Σ\|\|F_o_\| – \|F_c_\|\|/Σ\|F_o_\|; wR_2_ = [Σ [w(F_o_^2^ – Fc^2^)^2^]/ Σ[w(F_o_^2^)^2^]]^1/2^ | | | | | |

***Table S1.*** Continued.

| **Identification code** | **4** | **5** | **6** | **7** | ***P*^1m^** |
| --- | --- | --- | --- | --- | --- |
| CCDC | 2370322 | 2409949 | 2370323 | 2370324 | 2370325 |
| Empirical formula | C_100_H_71_F_12_N_7_O_12_P_2_Pt_2_S_4_ | C_88_H_64_F_6_N_6_O_6_P_2_Pt_2_S_2_ | C_59_H_43_F_3_N_3_O_3_PPtS | C_60_H_45_Cl_2_F_6_N_4_O_6_PPtS_2_ | C_32_H_23_P_1_ |
| Formula weight | 2370.99 | 1931.69 | 1157.08 | 1393.08 | 438.47 |
| Temperature (K) | 150(2) | | | | |
| Wavelength (Å) |  | 1.54184 | 0.71073 | | |
| Crystal system | Triclinic | Triclinic | Triclinic | Triclinic | Triclinic |
| Space group | $P\overline{1}$ | $P\overline{1}$ | $P\overline{1}$ | $P\overline{1}$ | $P\overline{1}$ |
| Unit cell dimensions |  |  |  |  |  |
| a (Å) | 9.1662(4) | 12.0616(2) | 15.2753(16) | 14.9079(14) | 9.1672(6) |
| b (Å) | 23.3810(11) | 16.8507(2) | 17.8669(19) | 15.3525(15) | 9.9850(9) |
| c (Å) | 23.8008(11) | 20.3552(2) | 21.535(3) | 15.716(3) | 13.7861(9) |
| a (^o^) | 62.237(2) | 82.9020(10) | 104.980(3) | 104.977(5) | 109.864(7) |
| β (^o^) | 82.723(2) | 75.9700(10) | 105.368(3) | 100.372(5) | 100.516(5) |
| γ (^o^) | 87.284(2) | 78.6030(10) | 104.324(2) | 117.981(3) | 97.534(6) |
| Volume (Å^3^) | 4476.9(4) | 3922.09(9) | 5150.1(11) | 2870.9(7) | 1141.15(16) |
| Z | 2 | 2 | 4 | 2 | 2 |
| ρ_calc_ (Mg/m^3^) | 1.759 | 1.636 | 1.492 | 1.612 | 1.276 |
| μ (mm^-1^) | 3.345 | 8.072 | 2.855 | 2.712 | 0.139 |
| F(000) | 2348 | 1908 | 2312 | 1388 | 460 |
| Crystal size (mm^3^) | 0.648 × 0.253 × 0.144 | 0.340 × 0.090 × 0.060 | 0.637 × 0.381 × 0.244 | 0.465 × 0.292 × 0.176 | 0.328 × 0.157 × 0.064 |
| θ range for data collection (^o^) | 2.335 to 33.000 | 2.244 to 80.013 | 2.233 to 27.999 | 2.086 to 34.361 | 2.205 to 30.343 |
| Index ranges | -14≤h≤14,  -35≤k≤35,  -36≤l≤36 | -15≤h≤15, -20≤k≤21,  -25≤l≤25 | -20≤h≤20,  -23≤k≤23,  -28≤l≤28 | -23≤h≤22,  -24≤k≤22,  -22≤l≤24 | -12≤h≤12,  -13≤k≤13,  -15≤l≤18 |
| Reflections collected | 133749 | 66087 | 102460 | 65968 | 10409 |
| Independent reflections | 33703 [R(int) = 0.0262] | 16672 [R(int) = 0.0449] | 24825 [R(int) = 0.0241] | 22948 [R(int) = 0.0232] | 6022 [R(int) = 0.0218] |
| Completeness to θ = 25.242^o^/67.684° | 99.9 % | 100.0 % | 99.7 % | 99.9 % | 100 % |
| Absorption correction |  | Numerical | | | Semi-empirical |
| Max. and min. transmission | 0.644 and 0.220 | 1.000 and 0.553 | 0.543 and 0.264 | 0.647 and 0.365 | 1.000 and 0.713 |
| Refinement method | Full-matrix least-squares on F^2^ | | | | |
| Data/ restrains/ parameters | 33703 / 0 / 1253 | 16672 / 12 / 1011 | 24825 / 268 / 1430 | 22948 / 67 / 813 | 6022 / 0 / 298 |
| GOOF on F^2^ | 1.029 | 1.069 | 1.027 | 1.030 | 1.051 |
| Final R indices [I>2σ(I)]^a^ | R_1_ = 0.0229, wR_2_ = 0.0493 | R_1_ = 0.0397, wR_2_ = 0.1047 | R_1_ = 0.0227, wR_2_ = 0.0527 | R_1_ = 0.0294, wR_2_ = 0.0626 | R_1_ = 0.0485, wR_2_ = 0.1004 |
| R indices (all data) | R_1_ = 0.0341, wR_2_ = 0.0543 | R_1_ = 0.0437, wR_2_ = 0.1082 | R_1_ = 0.0322, wR_2_ = 0.0579 | R_1_ = 0.0393, wR_2_ = 0.0657 | R_1_ = 0.0690, wR_2_ = 0.1114 |
| Largest diff. peak and hole (e.Å^-3^) | 1.314 and -0.877 | 1.725 and -1.419 | 1.688 and -1.239 | 1.119 and -1.464 | 0.386 and -0.347 |
| ^a^ R_1_ = Σ\|\|F_o_\| – \|F_c_\|\|/Σ\|F_o_\|; wR_2_ = [Σ [w(F_o_^2^ – Fc^2^)^2^]/ Σ[w(F_o_^2^)^2^]]^1/2^ | | | | | |

***Table S1.*** Continued.

| **Identification code** | ***P*^1e^_ox_** | **3e** | **8** | **9** |
| --- | --- | --- | --- | --- |
| CCDC | 2409950 | 2409946 | 2409947 | 2409948 |
| Empirical formula | C_34_H_23_OP | C_53_H_37_F_3_N_3_O_3_PPtS | C_96_H_70_F_6_N_8_O_6_P_2_Pt_2_S_2_ | C_92_H_64_F_12_N_8_O_12_P_2_Pt_2_S_4_ |
| Formula weight | 478.49 | 1078.97 | 2061.84 | 2281.87 |
| Temperature (K) | 150(2) | | | |
| Wavelength (Å) | 1.54184 | | | |
| Crystal system | Triclinic | Monoclinic | Triclinic | Triclinic |
| Space group | $P\overline{1}$ | *P*2_1_/*c* | $P\overline{1}$ | $P\overline{1}$ |
| Unit cell dimensions |  |  |  |  |
| a (Å) | 8.6698(2) | 16.1124(2) | 9.42980(10) | 10.0904(2) |
| b (Å) | 9.0831(2) | 18.6502(3) | 14.3934(2) | 13.1173(2) |
| c (Å) | 17.0883(3) | 14.57200(10) | 16.7582(3) | 17.4567(3) |
| a (^o^) | 91.412(2) | 90 | 111.325(2) | 96.6720(10) |
| β (^o^) | 101.635(2) | 94.6640(10) | 104.1310(10) | 95.3350(10) |
| γ (^o^) | 115.025(2) | 90 | 90.8070(10) | 108.974(2) |
| Volume (Å^3^) | 1185.10(5) | 4364.38(9) | 2041.33(6) | 2149.08(7) |
| Z | 2 | 4 | 1 | 1 |
| ρ_calc_ (Mg/m^3^) | 1.341 | 1.642 | 1.677 | 1.763 |
| μ (mm^-1^) | 1.224 | 7.329 | 7.807 | 8.085 |
| F(000) | 500 | 2144 | 1022 | 1126 |
| Crystal size (mm^3^) | 0.411 × 0.296 × 0.133 | 0.356 × 0.050 × 0.032 | 0.301 × 0.059 × 0.026 | 0.202 × 0.158 × 0.101 |
| θ range for data collection (^o^) | 2.661 to 79.807 | 2.752 to 79.981 | 2.938 to 79.859 | 2.573 to 80.169 |
| Index ranges | -10≤h≤10, -7≤k≤11,  -21≤l≤21 | -20≤h≤20,  -23≤k≤23,  -18≤l≤13 | -11≤h≤11,  -18≤k≤18,  -16≤l≤20 | -10≤h≤12,  -16≤k≤16,  -21≤l≤21 |
| Reflections collected | 16970 | 44285 | 32767 | 37109 |
| Independent reflections | 5003 [R(int) = 0.0304] | 9294 [R(int) = 0.0341] | 8619 [R(int) = 0.0469] | 9060 [R(int) = 0.0398] |
| Completeness to θ = 25.242^o^/67.684° | 99.9 % | 99.9 % | 100.0 % | 100.0 % |
| Absorption correction | Analytical | Numerical | | |
| Max. and min. transmission | 0.955 and 0.899 | 0.978 and 0.305 | 1.000 and 0.248 | 0.708 and 0.484 |
| Refinement method | Full-matrix least-squares on F^2^ | | | |
| Data/ restrains/ parameters | 5003 / 0 / 325 | 9294 / 0 / 587 | 8619 / 130 / 630 | 9060 / 0 / 596 |
| GOOF on F^2^ | 1.050 | 1.078 | 1.088 | 1.080 |
| Final R indices [I>2σ(I)]^a^ | R_1_ = 0.0369, wR_2_ = 0.0980 | R_1_ = 0.0293, wR_2_ = 0.0778 | R_1_ = 0.0305, wR_2_ = 0.0794 | R_1_ = 0.0298, wR_2_ = 0.0787 |
| R indices (all data) | R_1_ = 0.0383, wR_2_ = 0.0993 | R_1_ = 0.0315, wR_2_ = 0.0795 | R_1_ = 0.0323, wR_2_ = 0.0805 | R_1_ = 0.0309, wR_2_ = 0.0794 |
| Largest diff. peak and hole (e.Å^-3^) | 0.400 and -0.365 | 1.492 and -0.959 | 1.622 and -1.010 | 1.567 and -1.393 |
| ^a^ R_1_ = Σ\|\|F_o_\| – \|F_c_\|\|/Σ\|F_o_\|; wR_2_ = [Σ [w(F_o_^2^ – Fc^2^)^2^]/ Σ[w(F_o_^2^)^2^]]^1/2^ | | | | |

***Table S2.*** Selected bond lengths and angles for **1**.

| **compound** | **distances, Å** | | | **angles, deg.** |
| --- | --- | --- | --- | --- |
|  | **Ag–P** | **Ag–N** | **Ag–C_anthracene_** | **P–Ag–N** |
| **1** | 2.3621(6) | 2.218(2)  2.3911(19) | 3.102(2) | 144.97(6)  117.94(5) |

***Table S3.*** Selected bond lengths and angles for **2** (two sets of values correspond to two independent molecules found in the unit cell).

| **compound** | **distances, Å** | | | **angles, deg.** |
| --- | --- | --- | --- | --- |
|  | **Au–P** | **Au–I** | **Au–C_anthracene_** | **P–Au–I** |
| **2** | 2.2627(12)  2.2464(12) | 2.5638(4)  2.5500(4) | 3.149(5)  3.098(5) | 175.24(3)  176.28(3) |

***Table S4.*** Selected bond length and angles for **3**, **3m**, **5** and **6** (two sets of values for **5** and **6** correspond to two independent molecules found in the unit cell).

| **compound** | **distances, Å** | | | | **angles, deg.** |
| --- | --- | --- | --- | --- | --- |
|  | **Pt–P** | **Pt–C** | **Pt–N** | **Pt–C_anthracene_** | **P–Pt–N** |
| **3** | 2.2426(6) | 2.021(2) | 2.137(2)  2.008(2) | 3.162(3) | 174.83(6)  106.66(6) |
| **3m** (*P*–1) | 2.2523(7) | 2.026(3) | 2.147(2)  2.014(2) | 5.239(2) | 175.30(6)  107.24(6) |
| **3m** (*C*2/*c*) | 2.2514(18) | 2.032(8) | 2.147(7)  2.034(6) | 5.477(6) | 175.1(2)  106.53(19) |
| **5** | 2.2483(9)  2.2440(12) | 2.065(4)  2.051(5) | 2.108(4)  2.018(4)  2.130(4)  2.017(4) | 3.268(3)  3.288(5) | 172.94(11)  97.03(12)  175.73(11)  104.32(13) |
| **6** | 2.2480(7)  2.2434(7) | 2.027(2)  2.031(3) | 2.140(2)  2.011(2)  2.144(2)  2.002(2) | 3.178(3) | 174.79(6)  107.31(6)  174.73(6)  105.74(6) |

***Table S5.*** Selected bond length and angles for **4** and **7** (two sets of values for **4** correspond to two independent molecules found in the unit cell).

| **compound** | **distances, Å** | | | **angles, deg.** |
| --- | --- | --- | --- | --- |
|  | **Pt–P** | **Pt–N** | **Pt–C_anthracene_** | **P–Pt–N** |
| **4** | 2.3014(4)  2.2884(4) | 2.0734(14)  1.9906(14)  2.0571(14)  2.0621(14)  1.9881(14)  2.0506(14) | 3.230(2)  3.203(2) | 173.19(4)  99.34(4)  102.23(4)  171.67(4)  99.78(4)  101.32(4) |
| **7** | 2.2948(6) | 2.0511(15)  1.9858(15)  2.0507(15) | 3.223(2) | 175.23(4)  102.45(5)  98.89(5) |

***Table S6.*** Selected bond length and angles for **3e**, **8** and **9**.

| **compound** | **distances, Å** | | | | **angles, deg.** |
| --- | --- | --- | --- | --- | --- |
|  | **Pt–P** | **Pt–C** | **Pt–N** | **Pt–C_2_** | **P–Pt–N** |
| **3e** | 2.2510(7) | 2.019(3) | 2.154(2)  2.015(2) | 3.243(3) | 175.68(7)  106.61(7) |
| **8** | 2.2483(7) | 2.030(3) | 2.156(3)  2.019(3) | 3.179(3) | 174.89(8)  105.75(8) |
| **9** | 2.2917(8) | – | 2.070(3)  1.997(3)  2.054(3) | 3.180(3) | 176.21(9)  96.79(8)  104.30(8) |


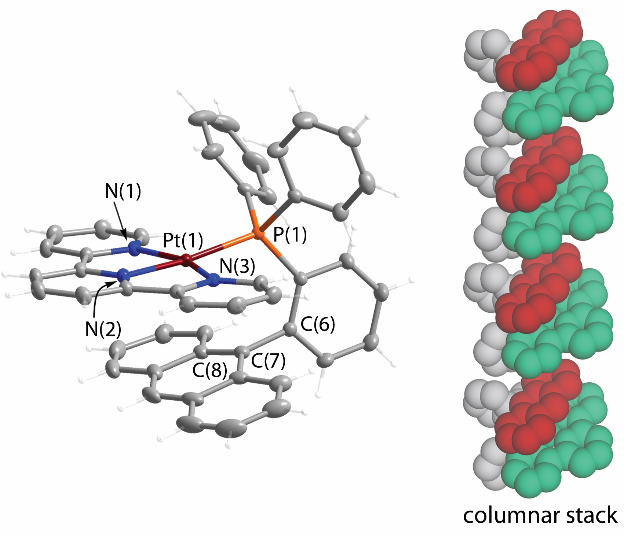


***Figure S1***. Molecular structure of complex **4** and its intermolecular stack (anthracene and platinum pincer fragments are shown in red and green, respectively; two independent molecules are found in the unit cell; displacement ellipsoids are shown at the 50% probability level; counterions are omitted for clarity).


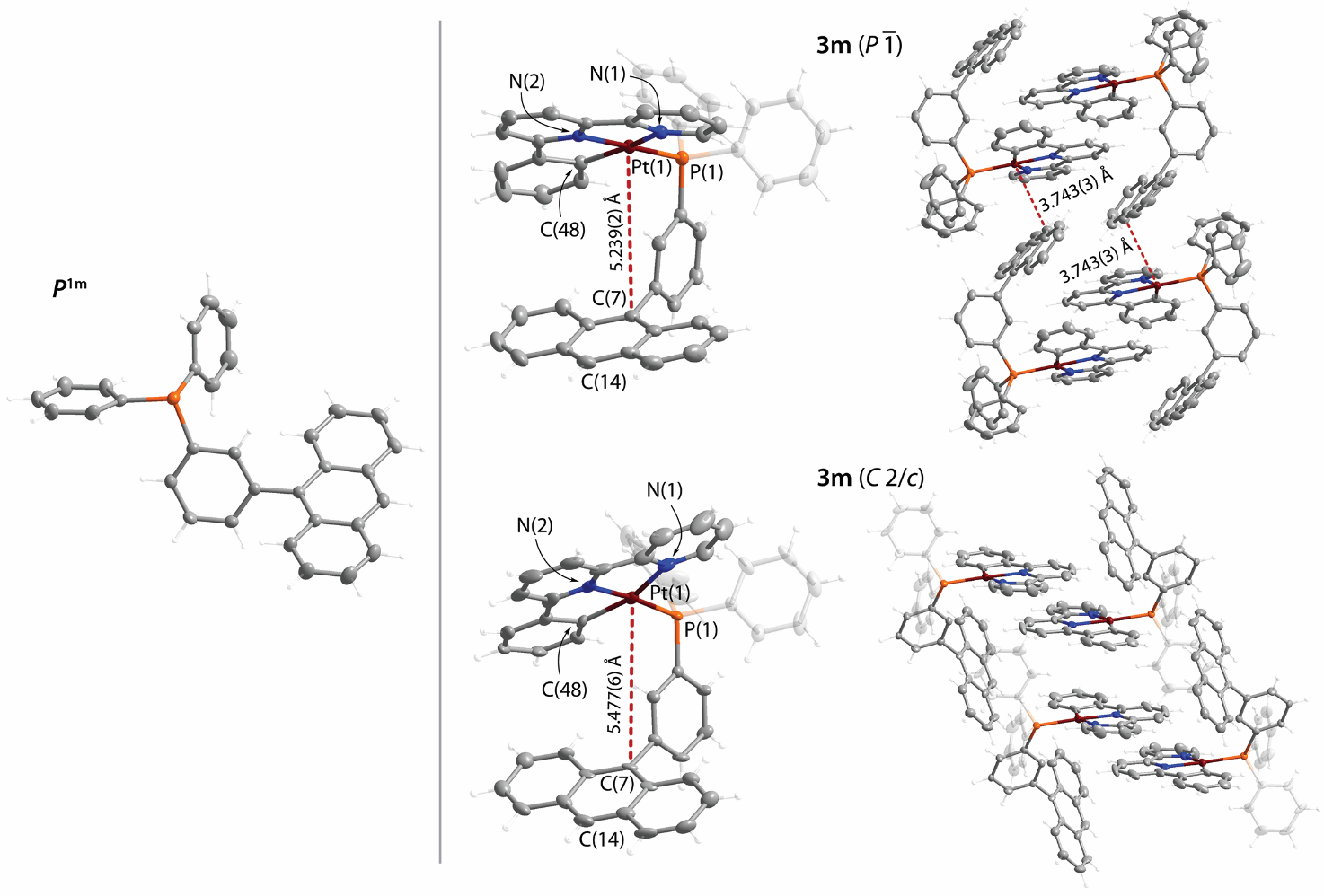


***Figure S2***. Molecular structures of ligand ***P*^1m^** and two modifications of complex **3m** (displacement ellipsoids are shown at the 50% probability level; counterions are omitted for clarity).


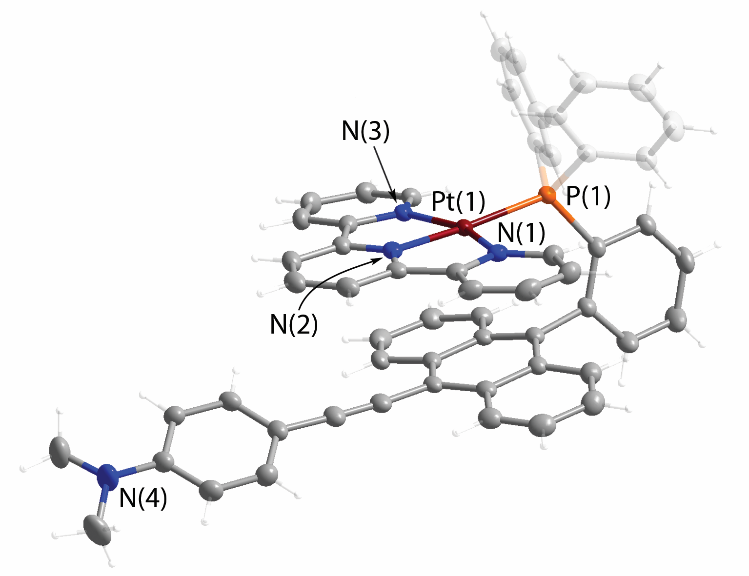


***Figure S3***. Molecular structure of complex **7** (displacement ellipsoids are shown at the 50% probability level; counterions are omitted for clarity).


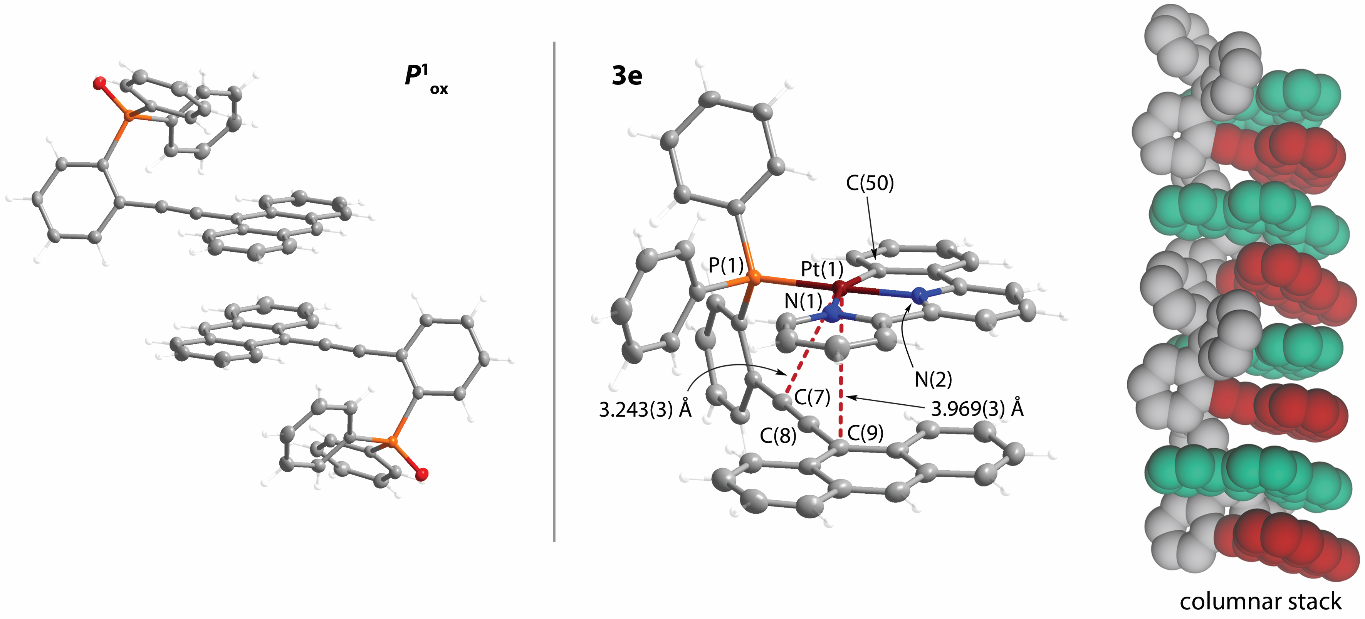


***Figure S4***. Molecular structures of phosphane oxide ***P*^1e^_ox_**, complex **3e** (displacement ellipsoids are shown at the 50% probability level; counterions are omitted for clarity) and the corresponding intermolecular stack of **3e** (right, ethynylanthracene and platinum pincer fragments are shown in red and green, respectively.


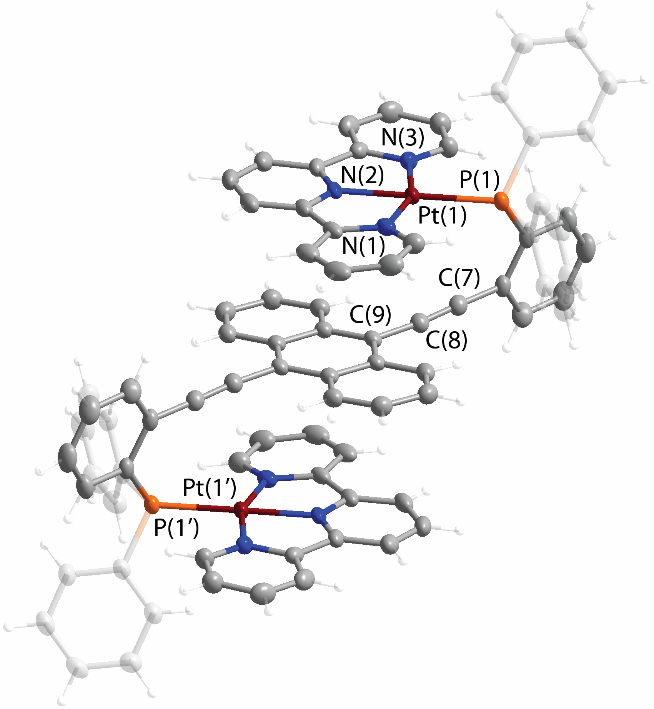


***Figure S5***. Molecular structure of complex **9** (displacement ellipsoids are shown at the 50% probability level; counterions are omitted for clarity).


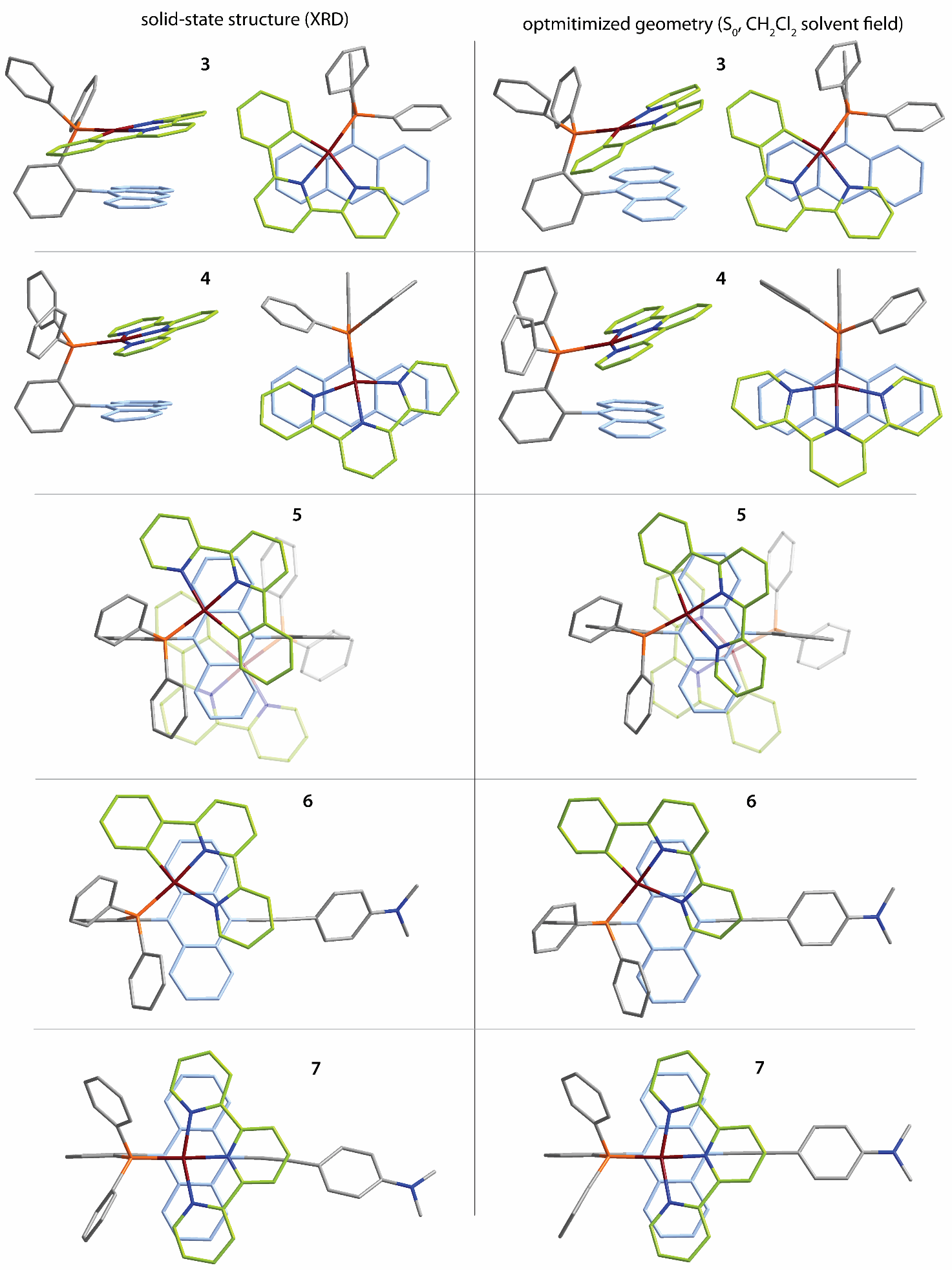


***Figure S6***. Crystallographically determined and DFT-optimized ground state geometries (CH_2_Cl_2_ solvation) of complexes **3**–**7**.


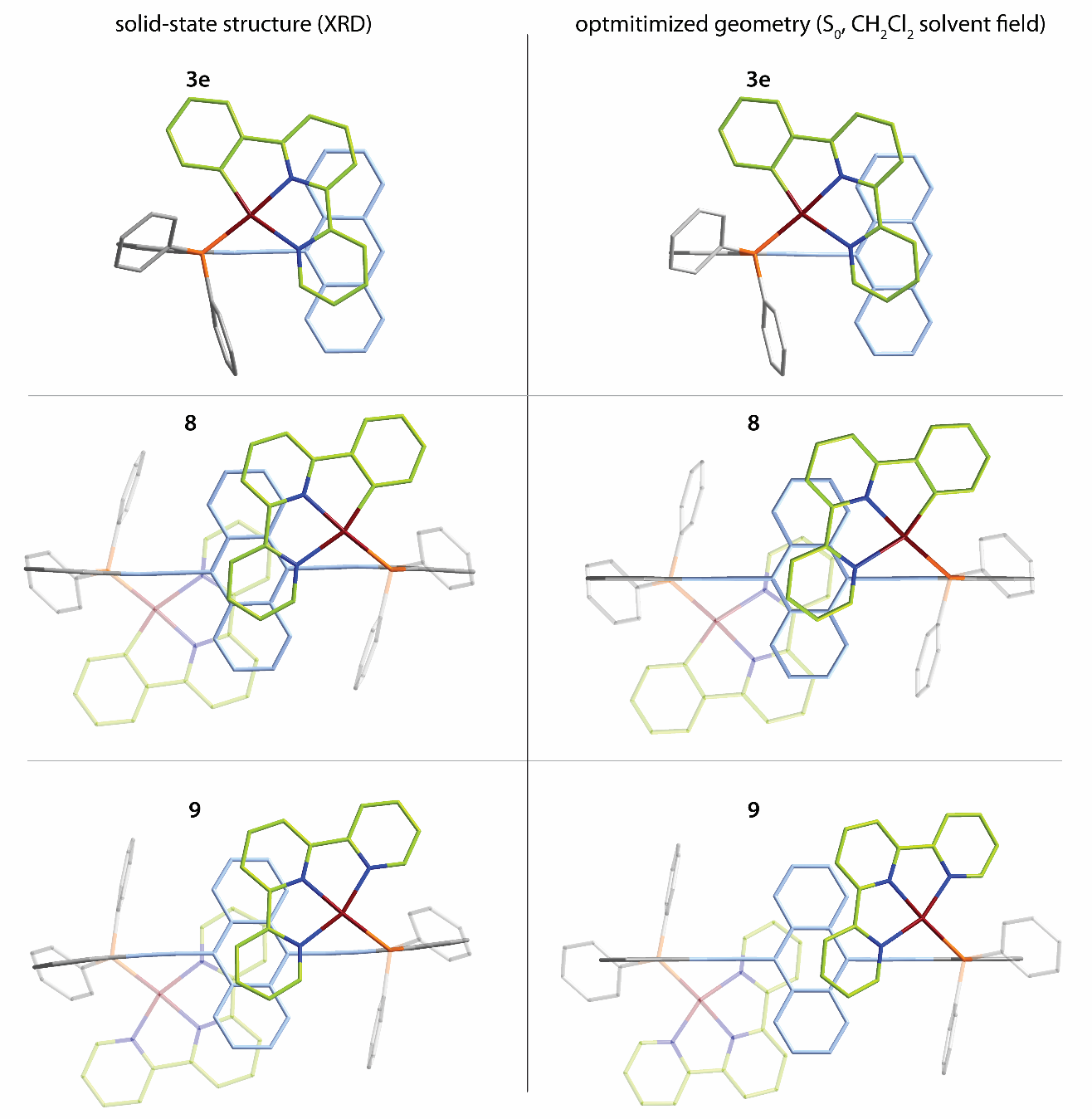


***Figure S7***. Crystallographically determined and DFT-optimized ground state geometries (CH_2_Cl_2_ solvation) of complexes **3e**, **8**, **9**.





***Figure S8***. ^31^P{^1^H} and ^1^H NMR spectra of complex **1** (CD_2_Cl_2_, 298 K).





***Figure S9***. ^31^P{^1^H} and ^1^H NMR spectra of complex **2** (CDCl_3_, 298 K).





***Figure S10***. ^31^P{^1^H} and ^1^H NMR spectra of complex **3** (acetonitrile-*d*_3_, 298 K).





***Figure S11***. ^31^P{^1^H} and ^1^H NMR spectra of complex **4** (acetonitrile-*d*_3_, 298 K).





***Figure S12***. ^31^P{^1^H} and ^1^H NMR spectra of complex **3m** (acetonitrile-*d*_3_, 298 K).





***Figure S13***. ^31^P{^1^H} and ^1^H NMR spectra of complex **5** (CD_2_Cl_2_, 298 K).





***Figure S14***. ^31^P{^1^H} and ^1^H NMR spectra of complex **6** (acetonitrile-*d*_3_, 298 K).





***Figure S15***. ^31^P{^1^H} and ^1^H NMR spectra of complex **7** (acetonitrile-*d*_3_, 298 K).





***Figure S16***. ^31^P{^1^H} and ^1^H NMR spectra of complex **3e** (CD_2_Cl_2_, 298 K).





***Figure S17***. ^31^P{^1^H} and ^1^H NMR spectra of complex **8** (acetonitrile-*d*_3_, 298 K).





***Figure S18***. ^31^P{^1^H} and ^1^H NMR spectra of complex **9** (acetonitrile-*d*_3_, 298 K, asterisks denote residual crystallization toluene).


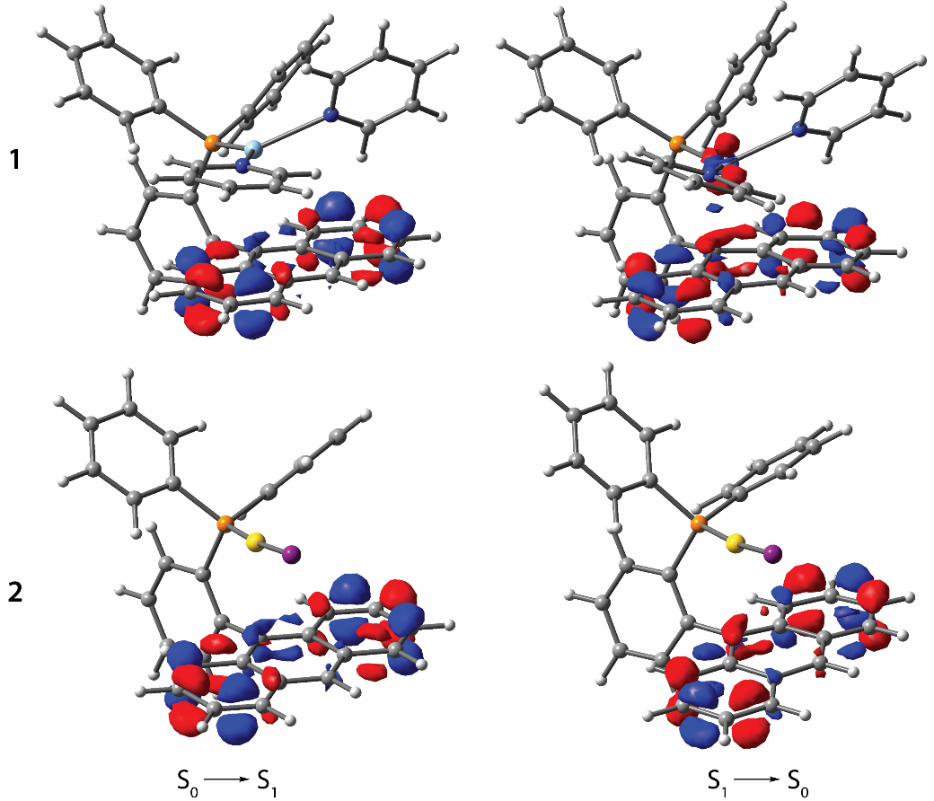


***Figure S19***. Electron density difference plots for complexes **1** and **2** (isovalue 0.001 a.u.). During the electronic transition, the electron density decreases in the blue areas and increases in the red areas.


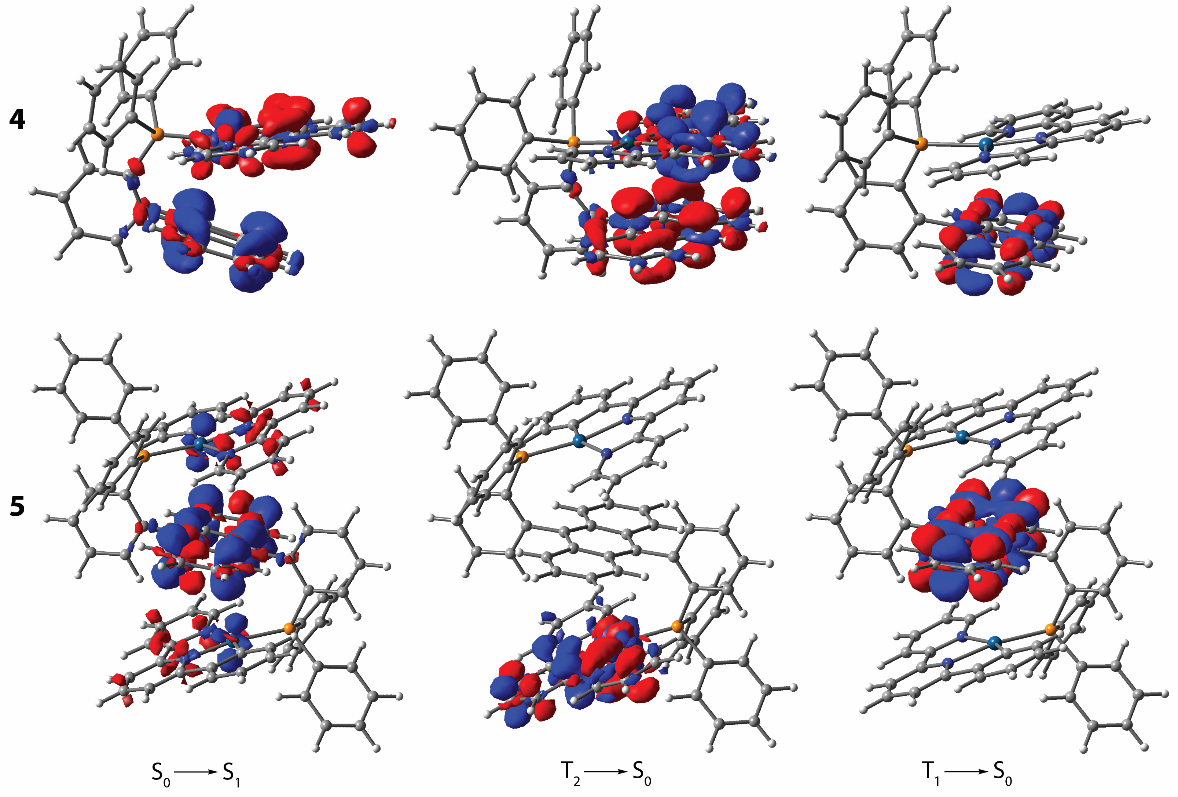


***Figure S20***. Electron density difference plots for complexes **4** and **5** (isovalue 0.001 a.u.). During the electronic transition, the electron density decreases in the blue areas and increases in the red areas. Note the calculated energy level is in the order of S_1_ ≥ T_2_ > T_1_ (Table S10).


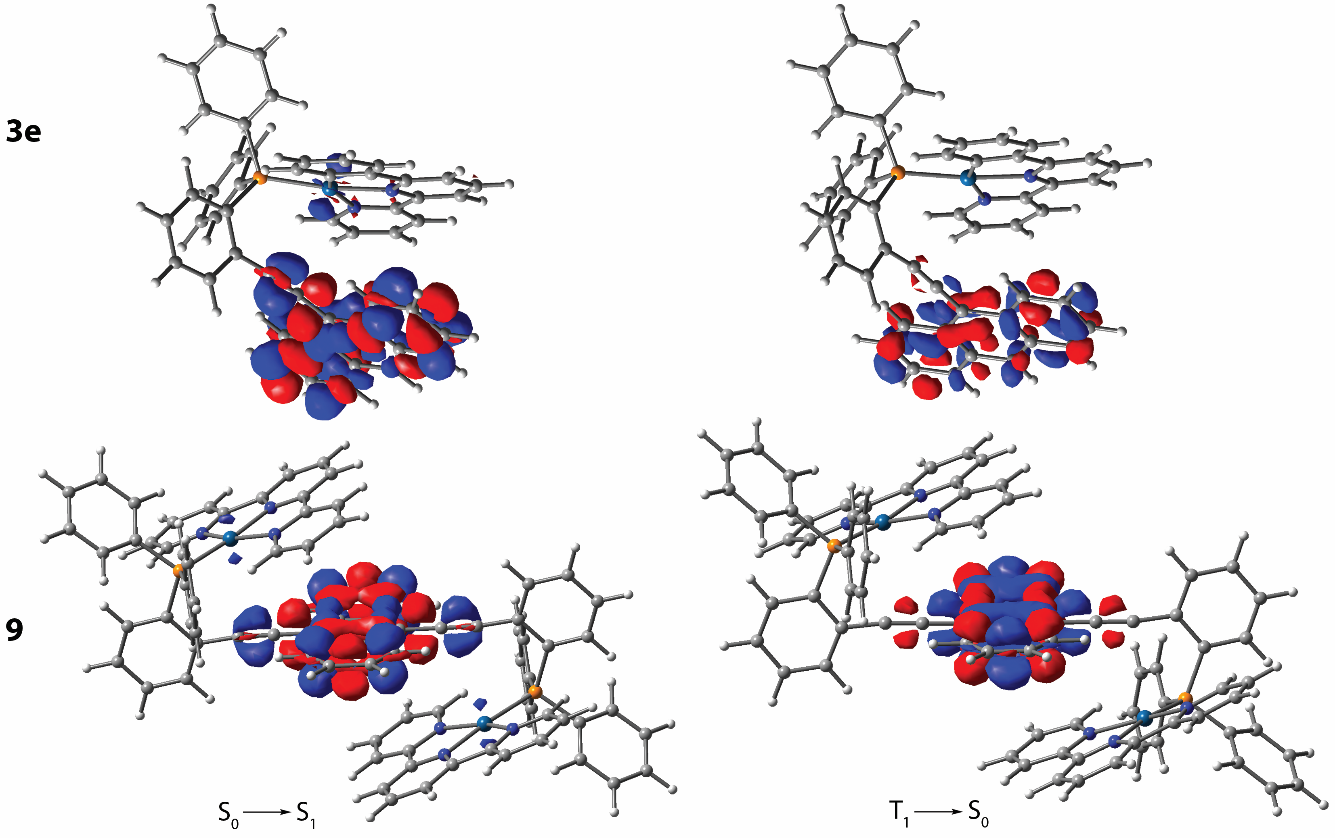


***Figure S21***. Electron density difference plots for complexes **3e** and **9** (isovalue 0.001 a.u.). During the electronic transition, the electron density decreases in the blue areas and increases in the red areas.


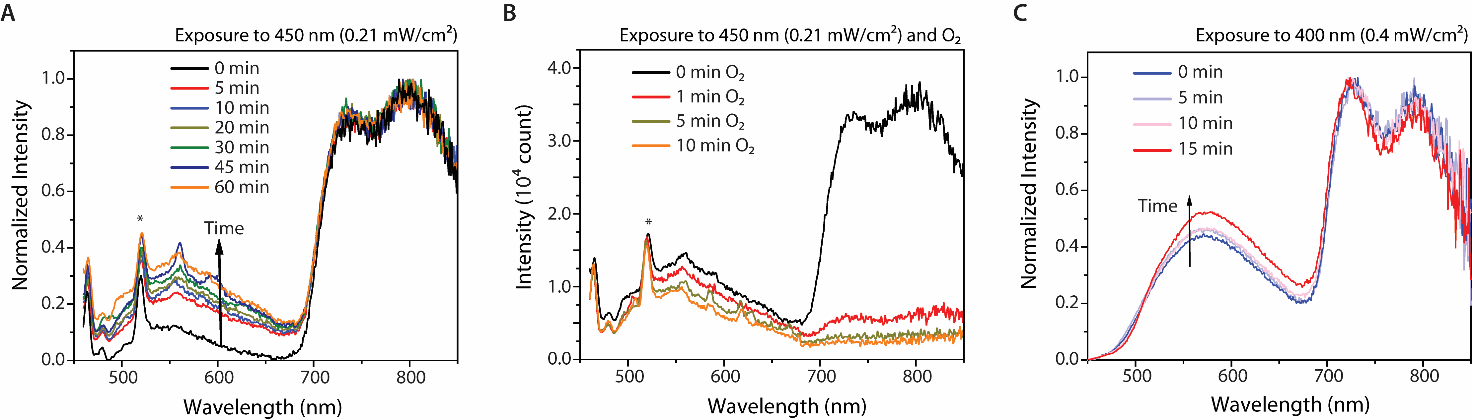


***Figure S22*.** Stability test for complex **3** in CH_2_Cl_2_ (see details of the experiment in the experimental section above). (A) Excitation at 450 nm (Intensity: 0.21 mW/cm^2^) at different times in degassed solution, followed by (B) bubbling with pure oxygen for varying durations. (C) Stability test for complex **3** in the film state (thickness: around 300 nm). The emerging high-energy band likely corresponds to a released Pt(phbipy) fragment and/or a product of intramolecular cyclization of the ligand ***P*^1^**.^[1]^


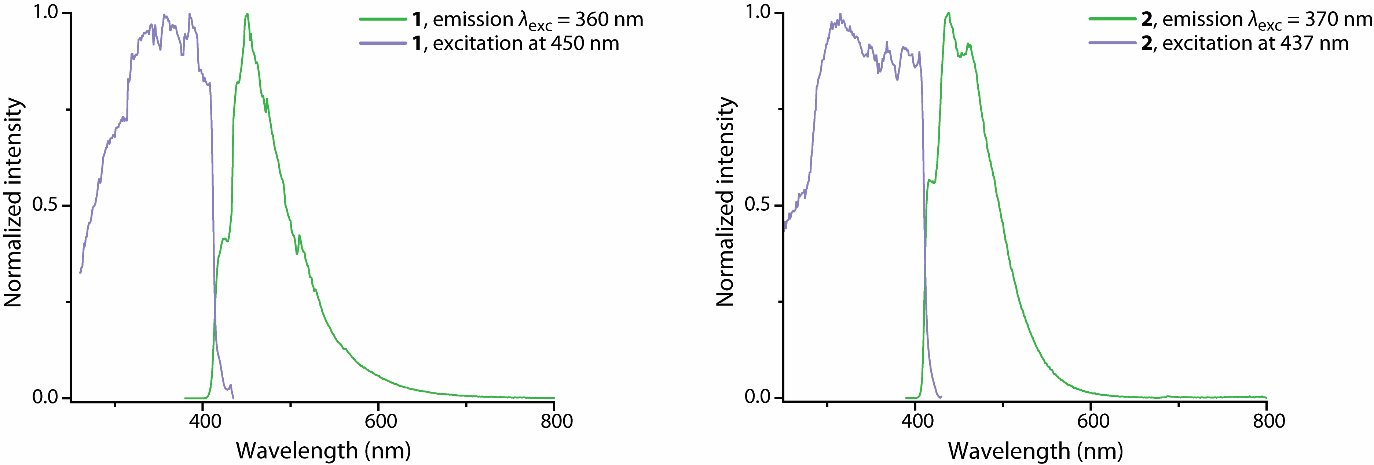


***Figure S23***. Normalized emission and excitation spectra of crystalline complexes **1** and **2** at 77 K.


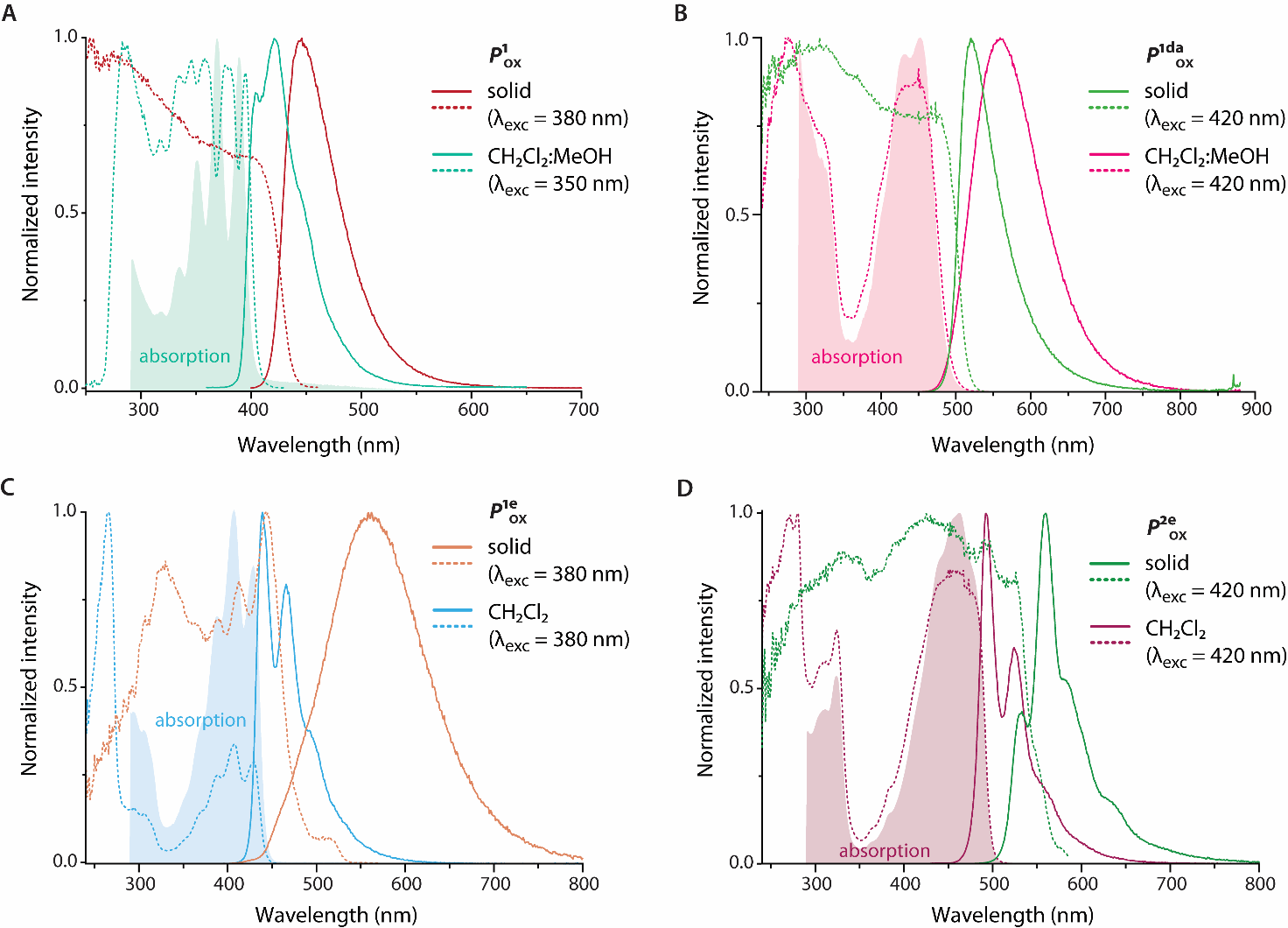


***Figure S24***. Normalized excitation (dashed lines), emission (solid lines) and absorption (filled profiles, CH_2_Cl_2_) spectra of phosphane oxides ***P*^1^_ox_**, ***P*^1da^_ox_**, ***P*^1e^_ox_**, and ***P*^2e^_ox_**.


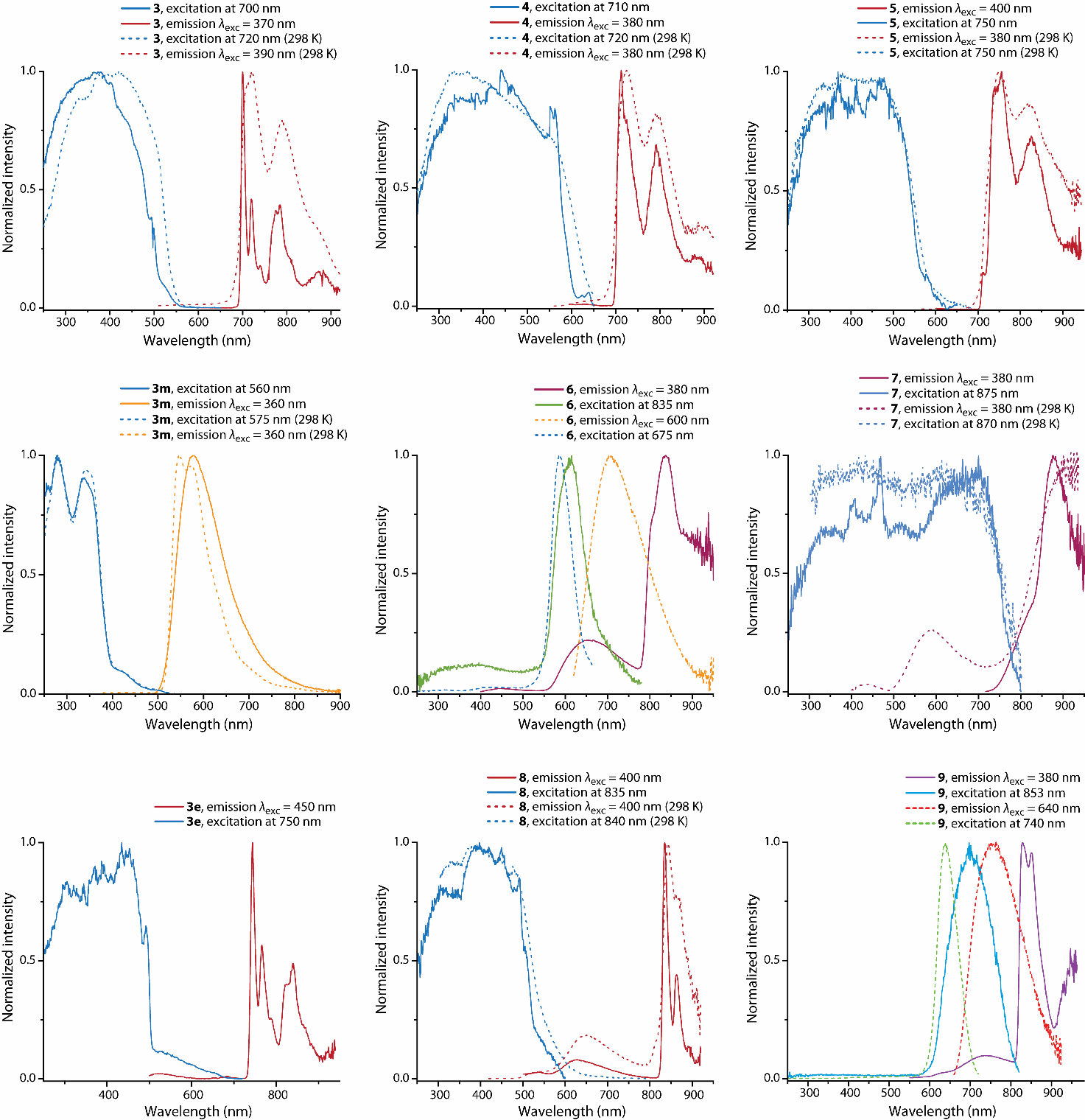


***Figure S25***. Normalized emission and excitation spectra of the crystalline complexes **3**–**9**, **3m**(*C*2/*c*) and **3e** at 77 K and at 298 K for **3**–**5**, **7**, **3m** and **8**.

***Table S7***. Photophysical properties of complexes **1**–**9**, **3m**, and **3e** in the solid state at 77 K.

|  | *λ*_exc_, nm | *λ*_em_, nm | *τ*_av_, µs*^a^* |
| --- | --- | --- | --- |
| **1** | 385 | 451 | (5.86 ± 0.04)^.^10^–3^ |
| **2** | 315 | 438 |  |
| **3** | 375 | 700, 719, 785 | 50.2 ± 0.5 |
| **3m** | 278, 341, 420sh | 547, 572 | 3.09 ± 0.03 |
| **4** | 440 | 712, 791 | 14.18 ± 0.05 |
| **5** | 369, 468 | 755, 822 | 15.3 ± 0.3 |
| **6** | 615 | 653, 838 | n.d. |
| **7** | 700 | 876 | n.d. |
| **3e** | 435 | 744 | 74.5 ± 1.1 |
| **8** | 397, 560sh | 835, 864 | 6.20 ± 0.04 |
| **9** | 697 | 735, 829, 851 | n.d. |

*^a^* Amplitude-weighted average lifetimes determined by the equation *τ*_av_ = Σ*A_i_τ_i_*, *A_i_* = weight of the *i*-th component.

***Table S8***. Photophysical properties of phosphane oxides in the solid state and solution at 298 K.

|  | **state** | ***λ*_abs_, nm**  **(*ε*, 10^‒3^ M^‒1^ cm^‒1^)*^a^*** | ***λ*_em_, nm** | ***Φ*_L_ (±0.02)** | ***τ*_av_, ns*^b^*** |
| --- | --- | --- | --- | --- | --- |
| ***P*^1^_ox_** | solid | – | 446 |  | 6.26 ± 0.01 |
|  | solution | 258 (88.6), 335 (3.7), 351 (6.6), 369 (9.9), 389 (9.4) | 404, 421*^c^* | 0.64*^c^* | 11.78 ± 0.02 |
| ***P*^1da^_ox_** | solid | – | 520 |  | 1.49 ± 0.01 |
|  | solution | 271 (36.7), 334 (12.3), 420 (19.4), 463 (20.1) | 560*^c^* | 0.82 | 4.63 ± 0.01 |
| ***P^1e^*_ox_** | solid | – | 560 | – | 0.73 ± 0.01 |
|  | solution | 266 (88.6), 295 (7.2), 366 (6.4), 386 (12.0), 406 (17.5), 429 (14.4) | 440, 466, 495, 530sh*^c^* | 0.37 | 3.78 ± 0.01 |
| ***P^2e^*_ox_** | solid | – | 530, 560, 585, 635 | – | 2.40 ± 0.01 |
|  | solution | 276 (80.1), 323 (20.1), 462 (37.2), 479 sh (29.9) | 492, 525, 565*^c^* | 0.95 | 3.50 ± 0.01 |

*^a^* CH_2_Cl_2_; *^b^* amplitude-weighted average lifetimes determined by the equation *τ*_av_ = Σ*A_i_τ_i_*, *A_i_* = weight of the *i*-th component *^c^* CH_2_Cl_2_:MeOH 1:1 v/v.

***Table S9***. TD-DFT-calculated lowest-lying singlet-singlet vertical excitations for **1**–**9**, **3m**, **3e** in CH_2_Cl_2_.

| **compound** | **transition** | ***E*, eV** | **λ, nm** | ***f****^a^* | **MLCT character, %** | **assignment** |
| --- | --- | --- | --- | --- | --- | --- |
| **1** | S_0_→S_1_ | 3.60 | 345 | 0.3274 | 1.2 | anthracene ππ* |
|  | S_0_→S_2_ | 4.08 | 304 | 0.0016 |  | anthracene ππ* |
| **2** | S_0_→S_1_ | 3.60 | 345 | 0.3182 | 1.9 | anthracene ππ* |
|  | S_0_→S_2_ | 4.07 | 305 | 0.0013 |  | anthracene ππ* |
| **3** | S_0_→S_1_ | 3.45 | 359 | 0.0897 | 13.5 | MLCT/LL’CT |
|  | S_0_→S_2_ | 3.49 | 356 | 0.0098 |  | MLCT/LL’CT |
| **3m** | S_0_→S_1_ | 3.46 | 358 | 0.0063 | 28.0 | MLCT/ILCT |
|  | S_0_→S_2_ | 3.64 | 341 | 0.3297 |  | anthracene ππ* |
| **4** | S_0_→S_1_ | 3.07 | 404 | 0.0424 | 1.2 | MLCT/LL’CT |
|  | S_0_→S_2_ | 3.58 | 347 | 0.2841 |  | anthracene ππ* |
| **5** | S_0_→S_1_ | 3.41 | 364 | 0.1280 | 1.5 | MLCT/anthracene ππ* |
|  | S_0_→S_2_ | 3.43 | 362 | 0.0000 |  | MLCT/IL |
| **6** | S_0_→S_1_ | 2.96 | 418 | 1.0992 | 1.0 | ILCT |
|  | S_0_→S_2_ | 3.39 | 365 | 0.0503 |  | MLCT/ILCT |
| **7** | S_0_→S_1_ | 2.67 | 464 | 0.1101 | 0.5 | ILCT/LL’CT |
|  | S_0_→S_2_ | 2.97 | 417 | 1.0152 |  | ILCT |
| **3e** | S_0_→S_1_ | 3.41 | 364 | 0.2895 | 8.9 | M perturbed anthracene π-π* |
|  | S_0_→S_2_ | 3.50 | 354 | 0.0209 |  | MLCT/IL |
| **8** | S_0_→S_1_ | 3.11 | 399 | 0.7139 | 2.6 | M perturbed anthracene π-π* |
|  | S_0_→S_2_ | 3.26 | 380 | 0.0001 |  | MLCT/IL |
| **9** | S_0_→S_1_ | 3.09 | 402 | 0.6792 | 6.0 | M perturbed anthracene π-π* |
|  | S_0_→S_2_ | 3.29 | 377 | 0.0000 |  | MLCT/LL’CT |

*^a^* Oscillator strength.

***Table S10***. DFT-calculated energy differences between S_1_, T_2_, and T_1_ states for **1**–**9**, **3m**, **3e** at S_1_ geometry.

| **compound** | **∆E(S_1_-T_1_), eV** | **∆E(S_1_-T_2_), eV** |
| --- | --- | --- |
| **1** | 1.48 | -0.01 |
| **2** | 1.51 | 0.02 |
| **3** | 1.21 | 0.33 |
| **3m** | 1.33 | 0.50 |
| **4** | 0.87 | -0.03 |
| **5** | 1.41 | 0.26 |
| **6** | 1.18 | -0.14 |
| **7** | 0.79 | -0.07 |
| **3e** | 0.84 | -0.17 |
| **8** | 0.94 | -0.29 |
| **9** | 1.07 | -0.29 |

***Table S11***. DFT-predicted singlet (S_1_→S_0_) and triplet (T_1_→S_0_) luminescence, and spin-orbit coupling matrix elements (SOCME) for **1**–**9**, **3m**, **3e**.

| **compound** | **fluorescence S_1_→S_0_** | | | **phosphorescence T_1_→S_0_** | | | **SOCME (S_1_–T_1_), cm^–1^ *^a^*** | **SOCME (S_1_–T_2_), cm^–1^ *^a^*** |
| --- | --- | --- | --- | --- | --- | --- | --- | --- |
|  | ***E*, eV** | **λ, nm** | **assignment** | ***E*, eV** | **λ, nm** | **assignment** |  |  |
| **1** | 2.92 | 424 | π-π* | 1.48 | 839 | ππ* | 13.4 | 11.9 |
| **2** | 3.03 | 409 | π-π* | 1.48 | 839 | ππ* | 6.3 | 11.8 |
| **3** | 2.40 | 517 | MLCT/LLCT | 1.45 | 853 | ππ* | 55.7 | 11.8 |
| **3m** | 2.78 | 446 | MLCT/ILCT | 1.50 | 827 | ππ* | 0.0 | 9.6 |
| **4** | 2.27 | 546 | MLCT/LLCT | 1.46 | 851 | ππ* | 28.9 | 38.6 |
| **5** | 2.84 | 436 | MLCT/π-π* | 1.40 | 883 | ππ* | 15.6 | 216.9 |
| **6** | 2.41 | 515 | ππ*/ILCT | 1.19 | 1040 | ππ* | 3.5 | 11.8 |
| **7** | 1.92 | 648 | MLCT/LLCT | 1.14 | 1091 | ππ* | 19.8 | 15.8 |
| **3e** | 1.78 | 698 | MLCT/π-π* | 1.35 | 916 | ππ* | 48.5 | 51.0 |
| **8** | 1.69 | 732 | MLCT/π-π* | 1.14 | 1084 | ππ* | 51.0 | 75.0 |
| **9** | 1.76 | 704 | MLCT/π-π* | 1.14 | 1088 | ππ* | 39.2 | 46.2 |

*^a^* At the S_1_ geometry.


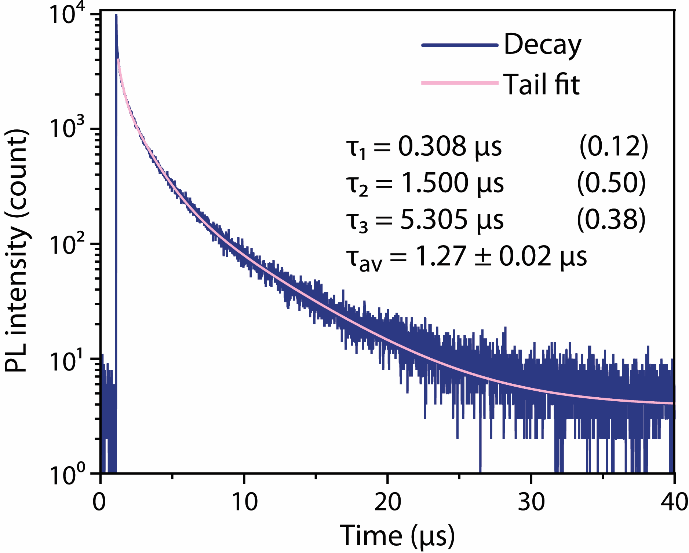


***Figure S26***. Lifetime data obtained by MCS for crystalline **3m**(*C*2/*c*) monitored at 575 nm (T_2_, ^3^MLCT emission band, λ_exc_ = 377 nm). Relative amplitudes are shown in parentheses.


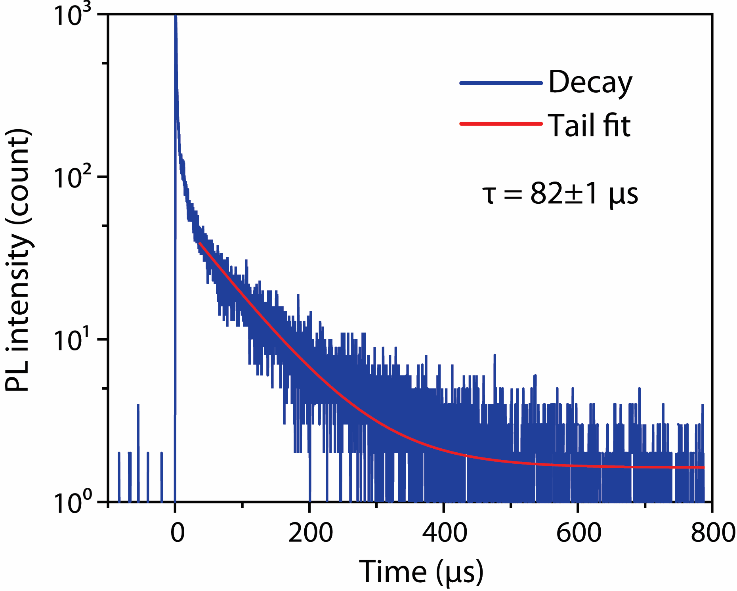


***Figure S27***. Lifetime data obtained by MCS for crystalline complex **3m**(*C*2/*c*) monitored at 780 nm (T_1_, ^3^π–π emission band, λ_exc_ = 532 nm).


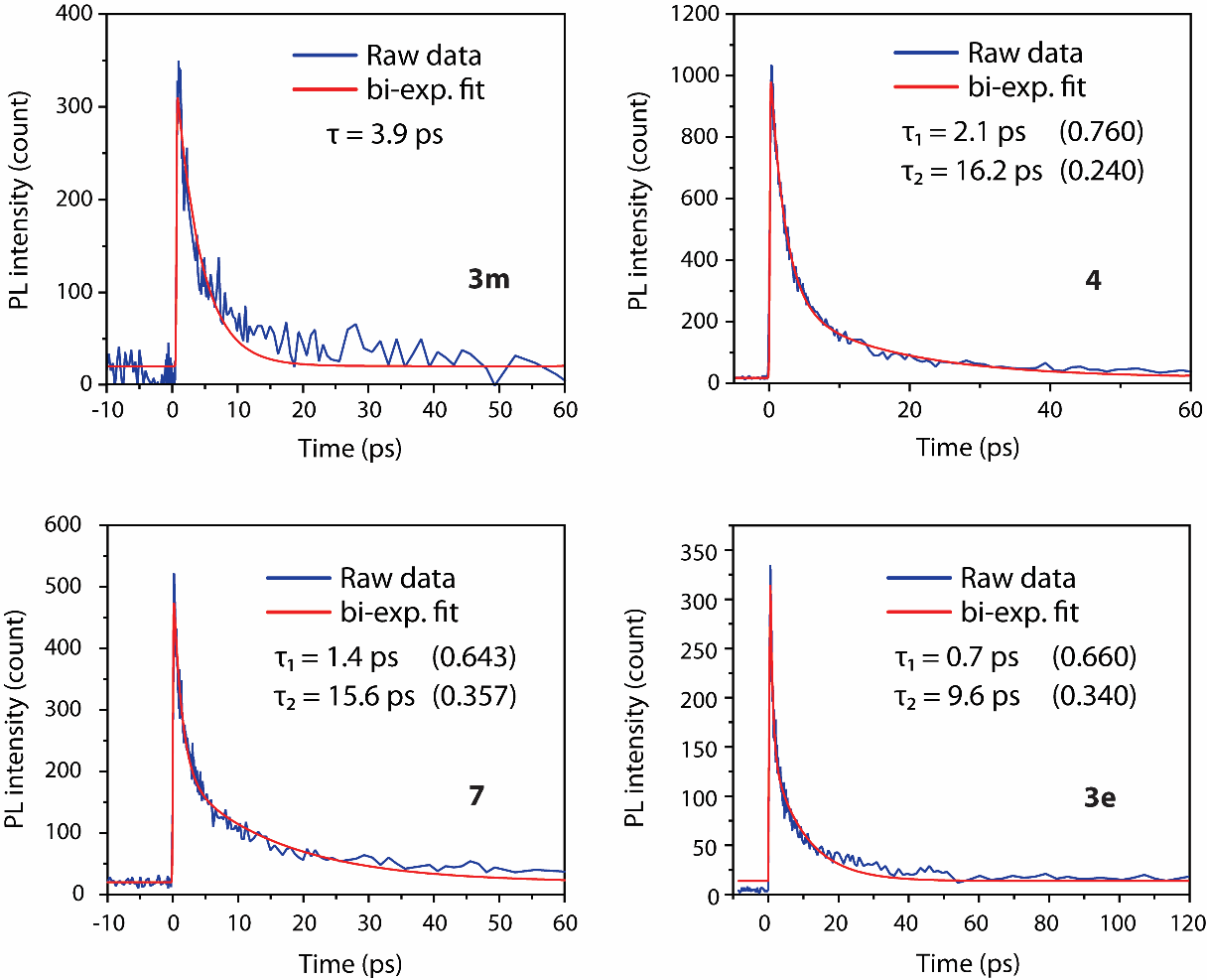


***Figure S28***. Early relaxation dynamics of crystalline complexes **3m**(*C*2/*c*), **4**, **7**, **3e** monitored at 600 nm emission (*λ*_exc_ = 410 nm). Relative amplitudes are shown in parentheses.

***Table S12***. Pertinent lifetime data for crystalline complexes **3**–**8**, **3m**, and **3e** determined using the fluorescence up-conversion technique.

| **compound** | $\boldsymbol{\tau}_{\mathbf{obs}}$ **(ps)** |
| --- | --- |
| **3** | 1.42 ± 0.01, 14.43 ± 0.03 |
| **3m** | 3.92 ± 0.01 |
| **4** | 2.14 ± 0.02, 16.22 ± 0.01 |
| **5** | 1.45 ± 0.02, 12.94 ± 0.03 |
| **6** | 0.97 ± 0.01, 10.35 ± 0.04 |
| **7** | 1.39 ± 0.01, 15.62 ± 0.02 |
| **3e** | 0.73 ± 0.01, 9.61 ± 0.01 |
| **8** | 0.81 ± 0.03, 6.14 ± 0.01 |


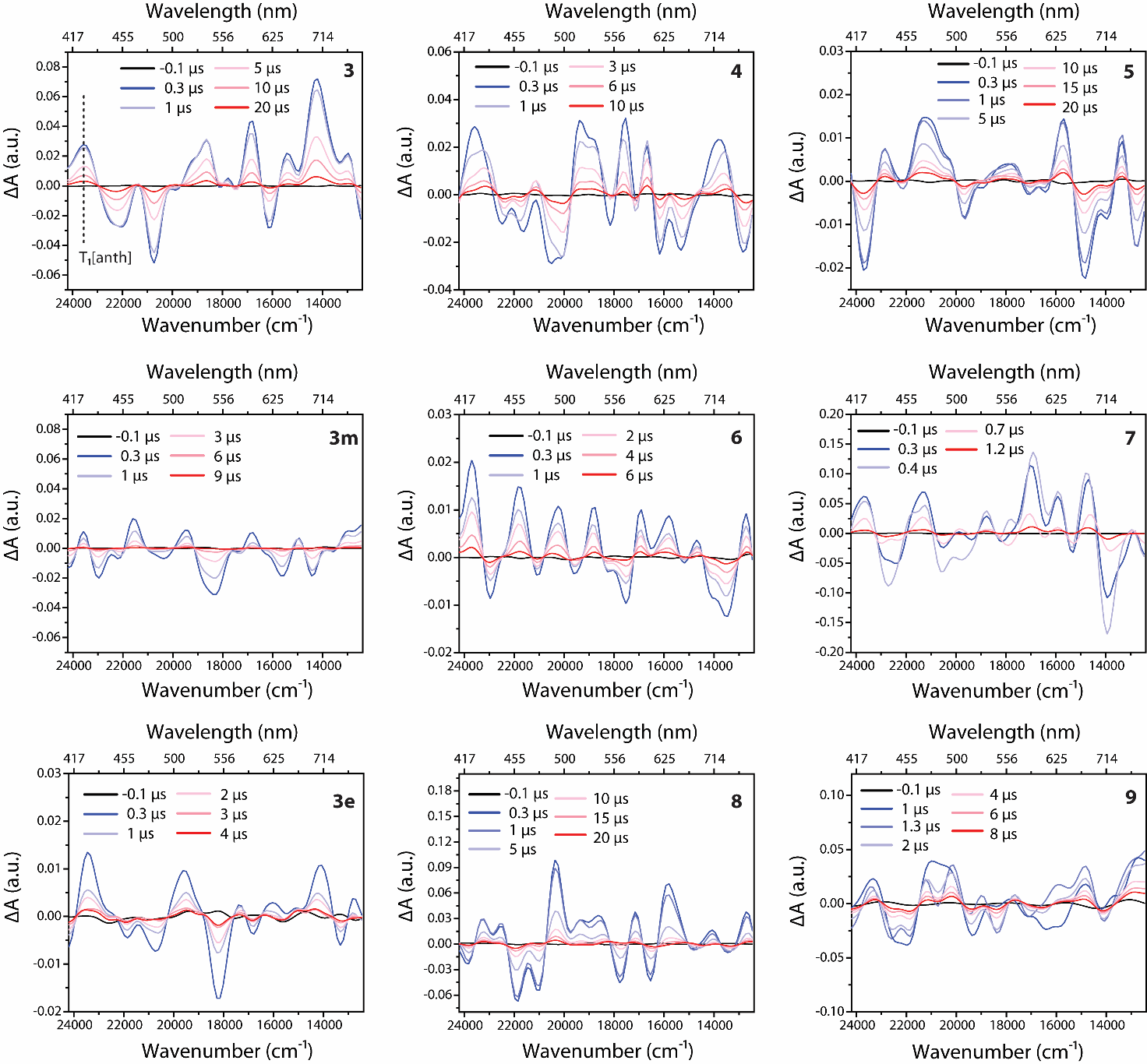


***Figure S29***. Nanosecond-transient absorption (ns-TA) data for crystalline complexes **3**–**9**, **3m** and **3e**. The anthracene triplet state absorption signal around 420 nm is indicated with a black dashed line for **3**.


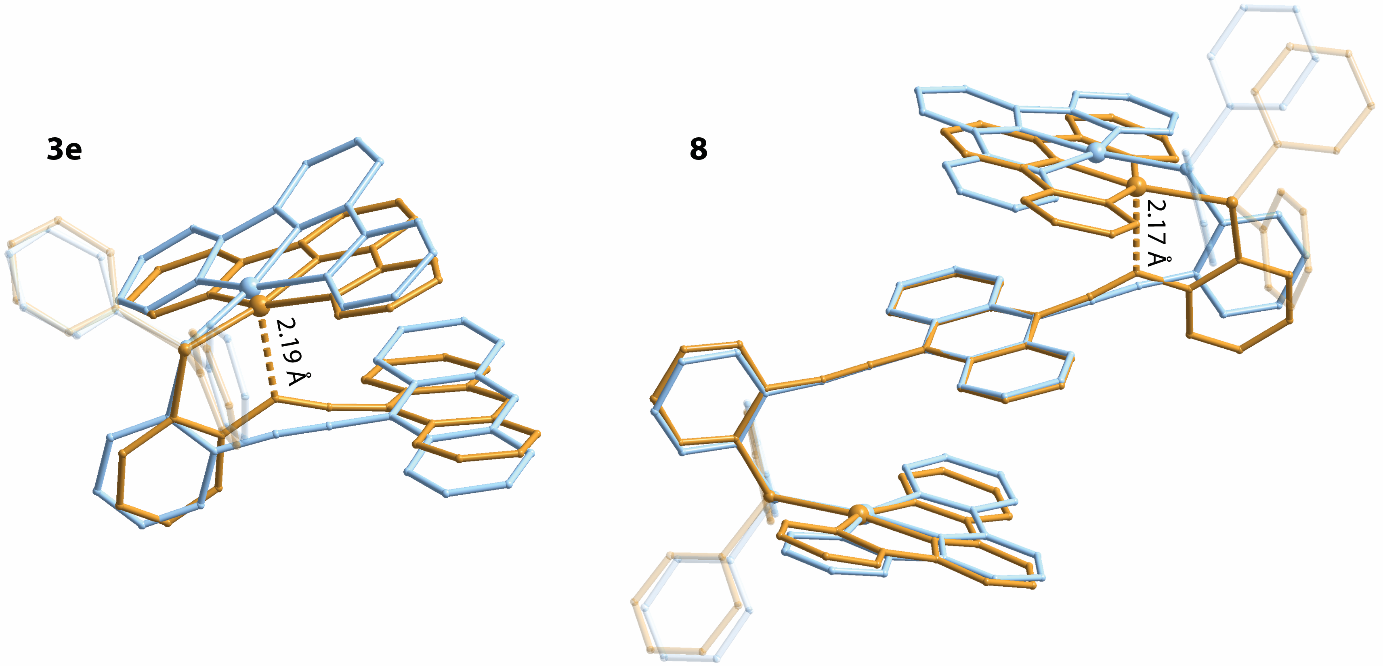


***Figure S30***. Overlay of optimized geometries of S_0_ (light blue) and S_1_ (gold) states for complexes **3e** and **8** showing the excited state Pt^…^C_2_ interaction.

**References**

[1] A. Belyaev, Y.-T. Chen, Z.-Y. Liu, P. Hindenberg, C.-H. Wu, P.-T. Chou, C. Romero-Nieto, I. O. Koshevoy, *Chem. Eur. J.* **2019**, *25*, 6332–6341.

[2] N. Ding, Z. Li, *Org. Lett.* **2020**, *22*, 4276-4282.

[3] T. Gazdag, E. Meiszter, P. J. Mayer, T. Holczbauer, H. Ottosson, A. B. Maurer, M. Abrahamsson, G. London, *ChemPhysChem* **2024**, *25*, e202300737.

[4] M. S. Khan, M. R. A. Al-Mandhary, M. K. Al-Suti, F. R. Al-Battashi, S. Al-Saadi, B. Ahrens, J. K. Bjernemose, M. F. Mahon, P. R. Raithby, M. Younus, N. Chawdhury, A. Kohler, E. A. Marseglia, E. Tedesco, N. Feederd, S. J. Teat, *Dalton Trans.* **2004**, 2377–2385.

[5] A. Belyaev, Y.-T. Chen, S.-H. Su, Y.-J. Tseng, A. J. Karttunen, S. P. Tunik, P.-T. Chou, I. O. Koshevoy, *Chem. Commun.* **2017**, *53*, 10954-10957.

[6] T.-C. Cheung, K.-K. Cheung, S.-M. Peng, C.-M. Che, *J. Chem. Soc., Dalton Trans.* **1996**, 1645-1651.

[7] T. M. Pappenfus, J. R. Burney, K. A. McGee, G. G. W. Lee, L. R. Jarvis, D. P. Ekerholm, M. Farah, L. I. Smith, L. M. Hinkle, K. R. Mann, *Inorg. Chim. Acta* **2010**, *363*, 3214-3221.

[8] *APEX2 - Software Suite for Crystallographic Programs*, Bruker AXS, Inc., Madison, WI, USA, **2010**.

[9] *CrysAlisPro*, Rigaku Oxford Diffraction, **2020**.

[10] G. M. Sheldrick, *SADABS-2008/1 - Bruker AXS Area Detector Scaling and Absorption Correction*, Bruker AXS, Madison, Wisconsin, USA, **2008**.

[11] G. M. Sheldrick, *Acta Crystallogr. C: Struct. Chem.* **2015**, *71*, 3-8.

[12] L. J. Farrugia, *J. Appl. Crystallogr.* **2012**, *45*, 849–854.

[13] A. L. Spek, *Acta Cryst.* **2015**, *C71*, 9–18.

[14] F. Neese, *WIREs Comput. Mol. Sci.* **2022**, e1606.

[15] V. Barone, M. Cossi, *J. Phys. Chem. A* **1998**, *102*, 1995–2001.

[16] M. A. Rohrdanz, K. M. Martins, J. M. Herbert, *J. Chem. Phys.* **2009**, *130*, 054112.

[17] F. Weigend, R. Ahlrichs, *Phys. Chem. Chem. Phys.* **2005**, *7*, 3297-3305.

[18] B. de Souza, G. Farias, F. Neese, R. Izsák, *J. Chem. Theory Comput.* **2019**, *15*, 1896-1904.

[19] a) F. Neese, *J. Comput. Chem.* **2003**, *24*, 1740-1747; b) F. Neese, F. Wennmohs, A. Hansen, U. Becker, *Chem. Phys.* **2009**, *356*, 98-109.
